# Correction: Contact-Inhibited Chemotaxis in De Novo and Sprouting Blood-Vessel Growth

**DOI:** 10.1371/journal.pcbi.1004163

**Published:** 2015-04-15

**Authors:** The PLOS Computational Biology Staff

There is an error within the main text (Results; 4^th^ paragraph) of this manuscript (at the end of page 3 in the PDF).

Currently, the 3^rd^ sentence reads:

“The diffusion coefficient of SDF-1/ CXCL12 is in the range of 1.7*10^(-13)m^2/s [39]”

It should read:

“The diffusion coefficient of SDF-1/ CXCL12 is in the range of 1.7*10^(-6)cm^2/s [39]”

## References

[1] Merks RMH, Perryn ED, Shirinifard A, Glazier JA(2008) Contact-Inhibited Chemotaxis in De Novo and Sprouting Blood-Vessel Growth. PLoS Comput Biol 4(9): e1000163 doi:10.1371/journal.pcbi.1000163 18802455 10.1371/journal.pcbi.1000163 PMC2528254

