# Supplementary material for: Contact-Inhibited Chemotaxis in De Novo and Sprouting Blood-Vessel Growth
Source: PLoS Comput Biol. 2008 Sep 19;4(9):e1000163. doi: 10.1371/journal.pcbi.1000163 (PMC2528254; doi:10.1371/journal.pcbi.1000163)
Supplement: Dataset S1 — Parameter files for the simulations shown in Figures 2, 4, 11, and 12, packed as a tar.gz archive To use, unpack the parameter-file archive and install the Tissue Simulation Toolkit (Protocol S1). Run the simulations from the command line using the command “vessel [parameter-file]”. Reproduce the other simulations by editing the parameter files using a standard text editor to set the values specified in the text. (4 KB ZIP) [file pcbi.1000163.s001.zip › ParameterFiles/index.html]

Supplementary information for: Contact-inhibited chemotactic motility in de novo and sprouting blood-vessel growth


# Parameter files for simulations presented in:

### Contact-inhibited chemotaxis in *de novo* and sprouting blood-vessel growth

### To be used with the Tissue Simulation Toolkit (TST 0.1.3 or higher)Roeland M.H. Merks1,2,3,\*,+, Erica D. Perryn4,++, Abbas Shirinifard3, and James A. Glazier3 1VIB Department of Plant Systems Biology, Technologiepark 927, B-9052 Ghent, Belgium 2Department of Molecular Genetics, Ghent University, B-9052 Ghent, Belgium 3The Biocomplexity Institute and Department of Physics, Indiana University Bloomington Swain Hall West 127, 727 E3rd Street, Bloomington, IN 47405-7105, USA 4The University of Kansas Medical Center, Dept. Anatomy and Cell Biology 1008 Wahl Hall West, 3901 Rainbow Boulevard, Kansas City, KS 66160 USA +Present address: Netherlands Institute for Systems Biology and CWI (Center for Mathematics and Computer Science) Kruislaan 413, 1098 SJ Amsterdam, The Netherlands ++Present address: Krumlauf Laboratory, Stowers Institute for Medical Research1000 East 50th Street, Kansas City, MO 64110, USA \*Correspondence: post@roelandmerks.nl Parameter files for use with the Tissue Simulation Toolkit (TST 0.1.3 or higher).To run these simulations, please download the Tissue Simulation Toolkit (version 0.1.3 or higher), and compile according to the instructions in the included file "INSTALL". After installation, download these parameter files to the source directory and start as: ./vessel [parameter file] from within the TST directory (*e.g* TST0.1.3). ***De novo* blood-vessel growth (vasculogenesis), as in Figure 2.** *Extension-retraction* chemotaxis - T = 50 - T = 200 - T = 50, no contact-inhibition *Extension-only* chemotaxis - T = 50 - T = 200 - T = 50, no contact-inhibition **Sprouting blood-vessel growth (angiogenesis), as in Figs. 4-13.** *Extension-retraction* chemotaxis - T = 50 - T = 200 - T = 50, no contact-inhibition *Extension-only* chemotaxis - T = 50 - T = 200 - T = 50, no contact-inhibition
