# Supplementary material for: Contact-Inhibited Chemotaxis in De Novo and Sprouting Blood-Vessel Growth
Source: PLoS Comput Biol. 2008 Sep 19;4(9):e1000163. doi: 10.1371/journal.pcbi.1000163 (PMC2528254; doi:10.1371/journal.pcbi.1000163)
Supplement: Protocol S1 — Tissue Simulation Toolkit v0.1.3. The source code for the software used for the simulations presented in this paper is also available from http://sourceforge.net/projects/tst. Installation: Unpack and compile according to the instructions given in the INSTALL file The code is written in C++ using the cross-platform (Windows, Mac, or Unix/Linux) library Qt (available from www.trolltech.com). (332 KB ZIP) [file pcbi.1000163.s002.zip › TST0.1.3/exercises.pdf]

1 The Cellular Potts Model

1.1 Introduction

In this exercise you will experiment with Glazier and Graner's Cellular Potts model [1], which they originally developed to study the differential-adhesion hypothesis (DAH) [2]. The DAH states that tissues self-organize as a result of their adhesive properties.

The CPM represents biological cells as patches of lattice sites, $\vec{x}$, with identical indices $\sigma(\vec{x})$, where each index identifies, or "labels" a single biological cell. Connections between neighboring lattice sites of unlike index $\sigma(\vec{x}) \neq \sigma(\vec{x}')$ represent membrane bonds, with a characteristic *bond energy* $J_{\tau(\sigma_{\vec{x}}),\tau(\sigma_{\vec{x}'})}$, where the cell types τ (*i.e.* endothelial, epidermal, *etc.*) determine the adhesion strength of the interacting cells. An energy penalty increasing with the cell's deviation from a designated target volume A_σ imposes a *volume constraint* on the biological cells.

We collect these effective energies in a *Hamiltonian*,

$$H = \sum_{\vec{x},\vec{x}'} J_{\tau(\sigma_{\vec{x}}),\tau(\sigma_{\vec{x}'})}(1 - \delta_{\sigma_{\vec{x}},\sigma_{\vec{x}'}}) + \lambda \sum_{\sigma} (a_\sigma - A_\sigma)^2, \tag{1}$$

where λ represents resistance to compression, a_σ is the current cell volume, and the Kronecker delta is $\delta_{x,y} = \{1, x = y; 0, x \neq y\}$. The cells reside in a "medium" which is a generalized CPM cell without a volume constraint and with $\sigma = 0$, and $\tau = 0$.

To mimic cytoskeletally-driven membrane fluctuations, we randomly choose a lattice site, $\vec{x}$, and attempt to copy its index $\sigma_{\vec{x}}$ into a randomly chosen neighboring lattice site $\vec{x}'$. We often use the eight, first and second order neighbors, but we can reduce the effects of lattice anisotropy by using the twenty, first- to fourth-order neighbors on a square lattice (in the parameter file you can change this behavior by using `neighbors = 2` or `neighbors = 3` respectively). On average, we attempt an update at each lattice site once per Monte-Carlo step (*MCS*). We calculate how much the energy would change if we performed the copy, and accept the attempt with probability:

$$P(\Delta H) = \{\exp(-(\Delta H + H_0)/T), \Delta H \geq -H_0; 1, \Delta H < -H_0\}, \tag{2}$$

where $H_0 >= 0$ is an energy threshold which models viscous dissipation and energy loss during bond breakage and formation[3].

The cells' adhesivities are expressed in terms of the *bond energies* $J_{\tau(\sigma_{\vec{x}}),\tau(\sigma_{\vec{x}'})}$, where *high* J-values imply *low* adhesivity and vice versa. It is convenient to describe the parameters in terms of the *surface tensions* $\gamma_{\tau_1,\tau_2} = J_{\tau_1,\tau_2} - (J_{\tau_1,\tau_1} + J_{\tau_2,\tau_2})/2$, which enable us to determine whether energetics favors homotypic ($\gamma_{\tau_1,\tau_2} > 0$) or heterotypic bonds ($\gamma_{\tau_1,\tau_2} < 0$) [1].

1.2 How to use the CPM software

The program `sorting` implements the basic Cellular Potts algorithm with two cell types. Used with the parameter file `sorting.par` the simulation initializes with a blob of cells to which the program assigns a random type 1 (red) or 2 (yellow).

Start the program by typing

```
sorting sorting.par
```

in a terminal or DOS-box. A window will appear with the initial cluster, which slowly disintegrates.

Now try to change the parameters in order to get the cells to sort out, to mix, and one type to engulf the other. To do so, first make a copy of the file `J.dat`, by typing:

```
copy Jnoadhesion.dat myJ.dat
```

or (for Linux)

```
cp Jnoadhesion.dat myJ.dat
```

in a terminal or DOS-box.

Now open the file `sorting.dat` in a text editor (*e.g.* notepad) and change the line:

```
Jtable = Jnoadhesion.dat
```

to

```
Jtable = myJ.dat
```

Then open the file myJ.dat. It will look something like this:

```
3
0
20 40
20 40 40
```

The file describes the diagonal matrix J; the first line is number of cell types, 3. That is two cell types plus one for the ECM.
The second line gives $J_{\mathrm{M,M}}$, the adhesion energy between “medium cells”. Since we only have one medium “cell”, this value is 0 by definition. The next line describes the adhesivities of cell type 1. The first number is its adhesion energy to the medium, $J_{\mathrm{M},1}$, the second the adhesion energy to cells of its own type, $J_{1,1}$. Similarly, the fourth line gives $J_{\mathrm{M},2}$, $J_{1,2}$ and $J_{2,2}$.

1.3 Exercises

1.3.1 Sorting it out

Change the J's to get cell sorting, cell mixing, and engulfment, respectively, and give the corresponding values of γ. Note that the values of J are integers, so make sure your value of J differ sufficiently; choosing them in the range 1..50 would be a good bet.

1.3.2 Wild cells, quiet cells; small cells, big cells

Also try experimenting with the values of the temperature T, A (`target_area`), and λ (`lambda`). How does the simulation respond to these changes? Does this meet your expectations? Can you philosophize to which biological or physical parameters these parameters would correspond?

2 Vasculogenesis modeling with the CPM

2.1 Introduction

In these exercises we will model different mechanisms of angiogenesis using the cellular Potts model. To do so, we need a partial differential equation layer to simulate the diffusion and decay of chemoattractants (*e.g.* VEGF-isoforms) in the ECM; also the CPM must be able to secrete chemicals into the ECM.

Our initial cell-centered model of vasculogenesis implements the basic assumption of the Gamba and Serini model [4, 5]: ECs migrate towards the chemoattract they themselves secrete. We use the basic *CPM*, and add a PDE layer which describes the diffusion and secretion of the chemoattractant in the uniform substrate underlying the cells:

$$\frac{\partial c}{\partial t} = \alpha\ \delta_{\sigma_{\vec{x}},0} - (1 - \delta_{\sigma_{\vec{x}},0})\epsilon\ c + D\nabla^2 c, \tag{3}$$

where $\delta_{\sigma_{\vec{x}},0} = 1$ inside the cells. α is the rate at which the cells release chemoattractant, ϵ is the decay rate of the chemoattractant, and D is the diffusion coefficient of the chemoattractant. Every site within the *CPM* cells secretes the chemoattractant, which only decays in the substrate. We solve this PDE numerically using a finite-difference scheme (forward Euler) on a lattice that matches the *CPM* lattice, using 15 diffusion steps per *MCS*. For these parameters, the chemoattractant diffuses more rapidly than the cells, enabling us to ignore advection as the cells push the substrate forward.

We implement preferential extension of filopodia in the direction of chemoattractant gradients — which drives chemotaxis — by allowing for an extra energy drop at the time of copying [6]:

$$\Delta H_{\text{chemotaxis}} = -\mu(c(\vec{x}') - c(\vec{x})), \tag{4}$$

where $\vec{x}'$ is the neighbor into which site $\vec{x}$ copies its spin, and $\mu = 500$ and $\mu = 0$ at cell-substrate and cell-cell interfaces respectively. We use a value of $\mu = 500$ to obtain sufficient chemotactic migration. In our initial simulations the cells do not adhere without chemotaxis ($J_{cc} = 2J_{cM}$).

2.2 Chemotaxis to autocrinically secreted factors

Start up a Cellular Potts simulation with the parameter file `chemotaxis.par`, by typing on the commandline:

```
vessel chemotaxis.par
```

As in the PDE model by Gamba *et al.*, the cells secrete a chemoattractant (shown in grey and with the green concentration isolines), and move towards higher concentrations of the attractant.

What patterns do you see? Does this meet your expectations, based on what you know from the Gamba-Serini continuum model?

Now experiment with the parameters, including the decay rate of the chemoattractant (`decay rate`), the diffusion coefficient (`diff_coeff`), and the number and size of the cells (`n_init_cells`, `target_area`. You can do so by editing the parameter file. What determines the size of the cell clusters?
First, copy `chemotaxis.par`, by typing:

```
cp chemotaxis.par vasculogenesis.par
```

Edit the copy of the parameter file with an editor, *e.g. notepad.*
Restart your simulation with:

```
vessel vasculogenesis.par
```

For some parameters (*e.g. high diffusion coefficients*) the diffusion algorithm will become numerically unstable; in those cases you can experiment with the number and size of PDE steps carried out after each Monte Carlo Step, using parameters `dt` and `pde_its`.

2.3 Making vascular networks

In the lectures we have discussed several mechanisms that help form vascular networks, including cell elongation and contact-inhibition of motility.

To mimic cell elongation due to cytoskeletal remodeling we add a cell- length constraint to the free energy:

$$H' = H + \lambda_L \sum_{\sigma} (l_\sigma - L_\sigma)^2, \quad (5)$$

where l_σ is the length of cell along its longest axis, L_σ its target length, and λ_L is the strength of the length constraint. Assuming that cells are ellipses, we can

derive their length from the largest eigenvalue of their inertia tensor I [7, 8]. The length constraint could cause cells to split into disconnected patches. We prevent this artifact by introducing a connectivity constraint, which reflects the physical continuity and cohesion of the actual cell [8].

2.3.1 Cell elongation

Start from a clean parameter file, by copying `chemotaxis.par` and editing the copy. Then play with the `target_length` (L); a good value to start with would be `target_length = 60` ($L = 120\ \mu m$, if `dx=2.0e-6`).

What happens? How are the cells organized? What seems to be the main driving force behind this mechanism?

Also try experimenting with small numbers of cells, say `n_init_cells = 10`. What happens to the polygonal pattern over time? If you are patient, you might also be interested in trying larger fields `sizex = 500` and `sizey = 500`. A useful parameter to change is also `subfield`; if you set this to 1.5 the cells will be distributed in a small space in the center of the field so the network has sufficient space to expand.

2.3.2 Passive cell shape changes

Again start from a clean copy of `chemotaxis.par`, and try experimenting with the cell adhesion between endothelial cells as we did in exercise 1. To do so, change the value of `Jtable` in your parameter file (`Jtable = adhesiveJ.dat`). In this parameter file $J_{cc} = 1$ and $J_{cM} = 20$.

What do you see? Is this what you expected?

Now, release the shape constraint from the endothelial cells, by setting `lambda2` = `0.0`, and run a new simulation. You might also want to use small target areas and use sm

What happens? What could be responsible for the change you see?

Go back to neutral cell adhesion settings ($J_{cc} = 40$, $J_{cM} = 20$), by setting `Jtable = J.dat`. Then experiment with the width of chemoattractant gradients (how would you do that?): do you notice any changes in the resulting patterns?

What is responsible for these (putative) vasculogenesis mechanisms? For further reading, see [9, 8].

2.3.3 Contact-inhibition of motility

Start with a clean copy of `chemotaxis.par`, in which the cell shape is constrained `lambda2 = 5.0`. Now, set parameter `vecadherinknockout` to `false`. As a result, we have turned off chemotaxis at cellular interfaces (we hypothesize that phosphorylation of VEGFR-2 receptors by vascular-endothelial cadherin causes such contact-inhibition of chemotaxis); the cells are only sensitive to the chemoattractant at cell-ECM interfaces.

What happens? Can you explain the resulting behavior?

Parameter file `contactinhibition.par` initializes the the simulation with a cell spheroid instead of dispersed cells. Run the simulation with the same parameters.

What happens? Do your observations help explain the role of contact-inhibition in vasculogenesis (see also [10]).

References

[1] J. A. Glazier and F. Graner. Simulation of the differential adhesion driven rearrangement of biological cells. *Phys. Rev. E*, 47:2128–2154, 1993.

[2] Malcolm S. Steinberg. Reconstruction of tissues by dissociated cells. *Science*, 141:401–408, 1963.

[3] P. Hogeweg. Evolving mechanisms of morphogenesis: on the interplay between differential adhesion and cell differentiation. *J. Theor. Biol.*, 203:317–333, 2000.

[4] A. Gamba, D. Ambrosi, A. Coniglio, A. De Candia, S. Di Talia, E. Giraudo, G. Serini, L. Preziosi, and F. Bussolino. Percolation morphogenesis and burgers dynamics in blood vessels formation. *Phys. Rev. Lett.*, 90:118101, 2003.

[5] G. Serini, D. Ambrosi, E. Giraudo, A. Gamba, L. Preziosi, and F. Bussolino. Modeling the early stages of vascular network assembly. *EMBO J.*, 22:1771–1779, 2003.

[6] N. J. Savill and P. Hogeweg. Modelling morphogenesis: from single cells to crawling slugs. *J. Theor. Biol.*, 184:229–235, 1997.

[7] M. Zajac, G. L. Jones, and J. A. Glazier. Simulating convergent extension by way of anisotropic differential adhesion. *J. Theor. Biol.*, 222:247–259, 2003.

[8] Roeland M.H. Merks, Segery V. Brodsky, Michael S. Goligorsky, Stuart A. Newman, and James A. Glazier. Cell elongation is key to in silico replication of in vitro vasculogenesis and subsequent remodeling. *Dev. Biol.*, 289:44–54, 2006.

[9] R. M. H. Merks, S. A. Newman, and J. A. Glazier. Cell-oriented modeling of *in vitro* capillary development. In Peter M.A. Sloot, Bastien Chopard, and Alfons G. Hoekstra, editors, *Cellular Automata. 6th international conference on Cellular Automata for Research and Industry*, volume 3305 of *Lect. Notes Comput. Sc.*, pages 425–434, Berlin, 2004. Spinger Verlag.

[10] Roeland M H Merks and James A Glazier. Contact-inhibited chemotactic motility can drive both vasculogenesis and sprouting angiogenesis. arXiv.org:q-bio/0505033, 2005.
