# Supplementary material for: Contact-Inhibited Chemotaxis in De Novo and Sprouting Blood-Vessel Growth
Source: PLoS Comput Biol. 2008 Sep 19;4(9):e1000163. doi: 10.1371/journal.pcbi.1000163 (PMC2528254; doi:10.1371/journal.pcbi.1000163)
Supplement: Protocol S1 — Tissue Simulation Toolkit v0.1.3. The source code for the software used for the simulations presented in this paper is also available from http://sourceforge.net/projects/tst. Installation: Unpack and compile according to the instructions given in the INSTALL file The code is written in C++ using the cross-platform (Windows, Mac, or Unix/Linux) library Qt (available from www.trolltech.com). (332 KB ZIP) [file pcbi.1000163.s002.zip › TST0.1.3/html/ca_8cpp.html]

Tissue Simulation Toolkit: ca.cpp File Reference

Main Page | Namespace List | Class Hierarchy | Class List | File List | Namespace Members | Class Members | File Members

# /home/romer/TST0.1.3/ca.cpp File Reference

`#include <stdio.h>`  
`#include <math.h>`  
`#include <cstdlib>`  
`#include "sticky.h"`  
`#include "random.h"`  
`#include "ca.h"`  
`#include "parameter.h"`  
`#include "dish.h"`  
`#include "sqr.h"`  
`#include "crash.h"`  
`#include "hull.h"`  
`#include "init.xpm"`  
`#include <fstream>`  

|  |
| --- |
|  |
| Namespaces | |
| namespace | std |
| Defines | |
| #define | ZYGFILE(Z)   <Z.xpm> |
| #define | XPM(Z)   Z ## \_xpm |
| #define | ZYGXPM(Z)   XPM(Z) |
| #define | ZYGOTE   init |
| Functions | |
| double | sat (double x) |
| Variables | |
| double | copyprob [BOLTZMANN] |
| Parameter | par |

---

## Define Documentation

|  |  |  |  |  |  |  |
| --- | --- | --- | --- | --- | --- | --- |
| |  |  |  |  |  |  | | --- | --- | --- | --- | --- | --- | | #define XPM | ( | Z |  | ) | Z ## \_xpm | |

|  |  |
| --- | --- |
|  |  |

|  |  |  |  |  |  |  |
| --- | --- | --- | --- | --- | --- | --- |
| |  |  |  |  |  |  | | --- | --- | --- | --- | --- | --- | | #define ZYGFILE | ( | Z |  | ) | <Z.xpm> | |

|  |  |
| --- | --- |
|  |  |

|  |  |
| --- | --- |
| |  | | --- | | #define ZYGOTE   init | |

|  |  |
| --- | --- |
|  |  |

|  |  |  |  |  |  |  |
| --- | --- | --- | --- | --- | --- | --- |
| |  |  |  |  |  |  | | --- | --- | --- | --- | --- | --- | | #define ZYGXPM | ( | Z |  | ) | XPM(Z) | |

|  |  |
| --- | --- |
|  |  |

---

## Function Documentation

|  |  |  |  |  |  |  |
| --- | --- | --- | --- | --- | --- | --- |
| |  |  |  |  |  |  | | --- | --- | --- | --- | --- | --- | | double sat | ( | double | *x* | ) |  | |

|  |  |
| --- | --- |
|  |  |

---

## Variable Documentation

|  |  |
| --- | --- |
| |  | | --- | | double copyprob[BOLTZMANN] | |

|  |  |
| --- | --- |
|  |  |

|  |  |
| --- | --- |
| |  | | --- | | Parameter par | |

|  |  |
| --- | --- |
|  |  |

---

Generated on Tue Dec 12 16:32:41 2006 for Tissue Simulation Toolkit by

1.3.5
