# Supplementary material for: Contact-Inhibited Chemotaxis in De Novo and Sprouting Blood-Vessel Growth
Source: PLoS Comput Biol. 2008 Sep 19;4(9):e1000163. doi: 10.1371/journal.pcbi.1000163 (PMC2528254; doi:10.1371/journal.pcbi.1000163)
Supplement: Protocol S1 — Tissue Simulation Toolkit v0.1.3. The source code for the software used for the simulations presented in this paper is also available from http://sourceforge.net/projects/tst. Installation: Unpack and compile according to the instructions given in the INSTALL file The code is written in C++ using the cross-platform (Windows, Mac, or Unix/Linux) library Qt (available from www.trolltech.com). (332 KB ZIP) [file pcbi.1000163.s002.zip › TST0.1.3/html/ca_8h-source.html]

Tissue Simulation Toolkit: /home/romer/TST0.1.3/ca.h Source File

Main Page | Namespace List | Class Hierarchy | Class List | File List | Namespace Members | Class Members | File Members

# /home/romer/TST0.1.3/ca.h

Go to the documentation of this file.

```
00001 /* 
00002 
00003 Copyright 1996-2006 Roeland Merks
00004 
00005 This file is part of Tissue Simulation Toolkit.
00006 
00007 Tissue Simulation Toolkit is free software; you can redistribute
00008 it and/or modify it under the terms of the GNU General Public
00009 License as published by the Free Software Foundation; either
00010 version 2 of the License, or (at your option) any later version.
00011 
00012 Tissue Simulation Toolkit is distributed in the hope that it will
00013 be useful, but WITHOUT ANY WARRANTY; without even the implied
00014 warranty of MERCHANTABILITY or FITNESS FOR A PARTICULAR PURPOSE.
00015 See the GNU General Public License for more details.
00016 
00017 You should have received a copy of the GNU General Public License
00018 along with Tissue Simulation Toolkit; if not, write to the Free
00019 Software Foundation, Inc., 51 Franklin St, Fifth Floor, Boston, MA
00020 02110-1301 USA
00021 
00022 */
00023 
00024 // mainpage.h contains no C++ code, it is for the main page of the
00025 // documentation
00026 #include "mainpage.h"
00027 
00029 #ifndef _CA_HH_
00030 #define _CA_HH_
00031 #include <vector>
00032 #include <stdio.h>
00033 #include "graph.h"
00034 #include "pde.h"
00035 //#include "dish.h"
00036 #include "cell.h"
00037 
00038 class Dish;
00039 
00040 class Dir {
00041   
00042   /* To store a celldirection matrix */
00043   friend class CellularPotts;
00044 public:
00045 
00046   Dir() {
00047     aa1=0.; aa2=0.;
00048     bb1=0.; bb2=0.;
00049     lb1=0.; lb2=0.;
00050   }
00051 
00052   double  aa1,aa2;
00053   double  bb1,bb2;
00054   double  lb1,lb2;
00055 };
00056 
00057 class CellularPotts {
00058 
00059   friend class Info;
00060   friend class Morphometry;
00061     
00062 public:
00064   CellularPotts(std::vector<Cell> *cells, const int sizex=200, 
00065                 const int sizey=200 );
00066   // empty constructor
00067   // (necessary for derivation)
00068   CellularPotts(void);
00069 
00070   // Keyword virtual means, that derived classed (cppvmCellularPotts) can override
00071   // this function and carry out the memory allocation in their preferred way
00072   // Every time AllocateSigma is called in the base class methods
00073   // the function belonging the actual type will be called
00074   virtual void AllocateSigma(int sx, int sy);
00075   
00076   // destructor must also be virtual
00077   virtual ~CellularPotts();
00078 
00086   int **SearchNandPlot(Graphics *g=0, bool get_neighbours=true);
00087   
00089   inline void Plot(Graphics *g) {
00090     SearchNandPlot(g, false);
00091   }
00092   
00094   inline int **SearchNeighbours(void) {
00095     return SearchNandPlot(0, true);
00096   }
00097 
00099   inline int Mass(void) {
00100     int mass=0;
00101     for (int i=0;i<sizex*sizey;i++) {
00102       if (sigma[0][i]>0) mass++;
00103     }
00104     return mass;
00105   }
00106 
00111   void PlotSigma(Graphics *g, int mag=2);
00112 
00113   
00115     void DivideCells(void) {
00116           std::vector<bool> tmp;
00117     DivideCells(tmp);
00118   }
00119   
00128     void DivideCells(std::vector<bool> which_cells);
00129     
00133     int AmoebaeMove(PDE *PDEfield=0);
00134   
00139     void ReadZygotePicture(void);
00140 
00141     
00142     // int BlobCounting(void); (not implemented?)
00143     
00144     // Used internally to assign a Cell object to each "blob"
00145     // "blobs" should already have different indices.
00146     //
00147     // (i.e. to start from a binary image you'd probably first want
00148     // to apply a blob counting algorithm
00149     void ConstructInitCells(Dish &beast);
00150     
00152     inline int Time() const {
00153       return thetime;
00154     }
00155   
00156 
00157     // not currently used? In Critter implementation (see Hogeweg
00158     // 2000) this was used to have cells divide at double their original area.
00159   inline int ZygoteArea() const {
00160     return zygote_area;
00161   }
00162 
00164   inline int SizeX() const {
00165     return sizex;
00166   }
00167   
00169   inline int SizeY() const {
00170     return sizey;
00171   }
00172   
00176   inline int Sigma(const int x, const int y) const {
00177     return sigma[x][y];
00178   }
00179   
00180   // Was used to make it possible to enlarge the Graphics window in
00181   // X11 and replace the contents interactively. Not currently supported.
00182   void Replace(Graphics *g);
00183 
00189   Dir *FindCellDirections(void) const;
00190 
00193   int ThrowInCells(int n, int cellsize);
00194 
00202   int GrowInCells(int n_cells, int cellsize, double subfield=1.);
00203   int GrowInCells(int n_cells, int cell_size, int sx, int sy, int offset_x, int offset_y);
00204   
00206   inline Cell &CellularPotts::AddCell(Dish &beast) {
00207     cell->push_back(Cell(beast));
00208     return cell->back();
00209   }
00215   void ShowDirections(Graphics &g, const Dir *celldir) const;
00216   
00218   double MeanCellArea(void) const;
00219   
00224   double CellDensity(void) const; 
00225   
00227   void ResetTargetLengths(void);
00228  
00229   int spins_converted;
00230   
00236   void SetRandomTypes(void);
00237 
00241   void GrowAndDivideCells(int growth_rate);
00242   
00243   inline Cell &getCell(int c) {
00244     return (*cell)[c];
00245   }
00246 
00250   double DrawConvexHull(Graphics *g, int color=1);
00251   
00256   double Compactness(double *res_compactness = 0, 
00257                      double *res_area = 0, 
00258                      double *res_cell_area = 0);
00259   
00260 private:
00261   void IndexShuffle(void);
00262   int DeltaH(int x,int y, int xp, int yp, PDE *PDEfield=0);
00263   bool Probability(int DH);
00264   void ConvertSpin(int x,int y,int xp,int yp);
00265   void SprayMedium(void);
00266   int CopyvProb(int DH,  double stiff);
00267   void FreezeAmoebae(void);
00268   void MeasureCellSizes(void);
00269   void MeasureCellSize(Cell &c);
00270   void CopyProb(double T);
00271   bool ConnectivityPreservedP(int x, int y);
00272 
00273   // little debugging function to print the site and its neighbourhood
00274   inline void PrintSite(int x,int y) {
00275           std::cerr << "--------\n";
00276           std::cerr << "[" << sigma[x-1][y-1] << " " << sigma[x][y-1] << " " << sigma[x+1][y-1] << "]\n";
00277           std::cerr << "[" << sigma[x-1][y] << " " << sigma[x][y] << " " << sigma[x+1][y] << "]\n";
00278           std::cerr << "[" << sigma[x-1][y+1] << " " << sigma[x][y+1] << " " << sigma[x+1][y+1] << "]\n";
00279   }
00280 
00281 protected:
00282         void BaseInitialisation(std::vector<Cell> *cell);
00283   
00284 protected:
00285   int **sigma;
00286   int sizex;
00287   int sizey;
00288   
00289 
00290 private:
00291   bool frozen;
00292   static const int nx[25], ny[25];
00293   static const int nbh_level[5];
00294   static int shuffleindex[9];
00295   std::vector<Cell> *cell;
00296   int zygote_area;
00297   int thetime;
00298   int n_nb;
00299 };
00300 
00301 
00302 #endif
```

---

Generated on Tue Dec 12 16:32:40 2006 for Tissue Simulation Toolkit by

1.3.5 
