# Supplementary material for: Contact-Inhibited Chemotaxis in De Novo and Sprouting Blood-Vessel Growth
Source: PLoS Comput Biol. 2008 Sep 19;4(9):e1000163. doi: 10.1371/journal.pcbi.1000163 (PMC2528254; doi:10.1371/journal.pcbi.1000163)
Supplement: Protocol S1 — Tissue Simulation Toolkit v0.1.3. The source code for the software used for the simulations presented in this paper is also available from http://sourceforge.net/projects/tst. Installation: Unpack and compile according to the instructions given in the INSTALL file The code is written in C++ using the cross-platform (Windows, Mac, or Unix/Linux) library Qt (available from www.trolltech.com). (332 KB ZIP) [file pcbi.1000163.s002.zip › TST0.1.3/html/cell_8cpp.html]

Tissue Simulation Toolkit: cell.cpp File Reference

Main Page | Namespace List | Class Hierarchy | Class List | File List | Namespace Members | Class Members | File Members

# /home/romer/TST0.1.3/cell.cpp File Reference

`#include <list>`  
`#include <vector>`  
`#include <stdio.h>`  
`#include <fstream>`  
`#include "cell.h"`  
`#include "sticky.h"`  
`#include "parameter.h"`  
`#include "dish.h"`  

|  |
| --- |
|  |
| Defines | |
| #define | HASHCOLNUM   255 |
| Variables | |
| Parameter | par |

---

## Define Documentation

|  |  |
| --- | --- |
| |  | | --- | | #define HASHCOLNUM   255 | |

|  |  |
| --- | --- |
|  |  |

---

## Variable Documentation

|  |  |
| --- | --- |
| |  | | --- | | Parameter par | |

|  |  |
| --- | --- |
|  |  |

---

Generated on Tue Dec 12 16:32:41 2006 for Tissue Simulation Toolkit by

1.3.5
