# Supplementary material for: Contact-Inhibited Chemotaxis in De Novo and Sprouting Blood-Vessel Growth
Source: PLoS Comput Biol. 2008 Sep 19;4(9):e1000163. doi: 10.1371/journal.pcbi.1000163 (PMC2528254; doi:10.1371/journal.pcbi.1000163)
Supplement: Protocol S1 — Tissue Simulation Toolkit v0.1.3. The source code for the software used for the simulations presented in this paper is also available from http://sourceforge.net/projects/tst. Installation: Unpack and compile according to the instructions given in the INSTALL file The code is written in C++ using the cross-platform (Windows, Mac, or Unix/Linux) library Qt (available from www.trolltech.com). (332 KB ZIP) [file pcbi.1000163.s002.zip › TST0.1.3/html/cell_8h-source.html]

Tissue Simulation Toolkit: /home/romer/TST0.1.3/cell.h Source File

Main Page | Namespace List | Class Hierarchy | Class List | File List | Namespace Members | Class Members | File Members

# /home/romer/TST0.1.3/cell.h

Go to the documentation of this file.

```
00001 /* 
00002 
00003 Copyright 1996-2006 Roeland Merks
00004 
00005 This file is part of Tissue Simulation Toolkit.
00006 
00007 Tissue Simulation Toolkit is free software; you can redistribute
00008 it and/or modify it under the terms of the GNU General Public
00009 License as published by the Free Software Foundation; either
00010 version 2 of the License, or (at your option) any later version.
00011 
00012 Tissue Simulation Toolkit is distributed in the hope that it will
00013 be useful, but WITHOUT ANY WARRANTY; without even the implied
00014 warranty of MERCHANTABILITY or FITNESS FOR A PARTICULAR PURPOSE.
00015 See the GNU General Public License for more details.
00016 
00017 You should have received a copy of the GNU General Public License
00018 along with Tissue Simulation Toolkit; if not, write to the Free
00019 Software Foundation, Inc., 51 Franklin St, Fifth Floor, Boston, MA
00020 02110-1301 USA
00021 
00022 */
00023 #ifndef _CELL_HH_
00024 #define _CELL_HH_
00025 
00026 #include "parameter.h"
00027 //#define EMPTY -1
00028 #include <math.h>
00029 
00030 extern Parameter par;
00031 class Dish;
00032 
00033 class Cell
00034 { 
00035 
00036   friend class Dish;
00037   friend class CellularPotts;
00038   friend class Info;
00039 
00040 public:
00046   Cell(const Dish &who, int
00047   settau=1) {
00048     
00049     owner=&who;
00050     ConstructorBody(settau);
00051   }
00052 
00053   Cell(void) {
00054     chem = new double[par.n_chem];
00055   };
00056   
00057   ~Cell(void);
00058   
00059   
00061   Cell(const Cell &src) {
00062         
00063     // make an exact copy (for internal use)
00064     sigma=src.sigma;
00065     amount++;
00066     area=src.area;
00067     target_area=src.target_area;
00068     length=src.length;
00069     target_length=src.target_length;
00070     growth_threshold=src.growth_threshold;
00071     mother=src.mother;
00072     daughter=src.daughter;
00073     times_divided=src.times_divided;
00074     date_of_birth=src.date_of_birth;
00075     colour_of_birth=src.colour_of_birth;
00076     tau=src.tau;
00077     alive=src.alive;
00078     v[0]=src.v[0];
00079     v[1]=src.v[1];
00080     n_copies=src.n_copies;
00081     sum_x=src.sum_x;
00082     sum_y=src.sum_y;
00083     sum_xx=src.sum_xx;
00084     sum_yy=src.sum_yy;
00085     sum_xy=src.sum_xy;
00086     owner=src.owner;
00087     
00088     chem = new double[par.n_chem];
00089     for (int ch=0;ch<par.n_chem;ch++)
00090       chem[ch]=src.chem[ch];
00091     
00092     
00093   }
00094   
00103   Cell(Cell &mother, int settau=1);
00104  
00111   inline Cell &operator=(const Cell &src) {
00112     colour=src.colour;
00113     alive=src.alive;
00114     sigma=src.sigma;
00115     area=src.area;
00116     tau=src.tau;
00117     target_area=src.target_area;
00118     v[0]=src.v[0];
00119     v[1]=src.v[1];
00120     n_copies=src.n_copies;
00121     
00122     sum_x=src.sum_x;
00123     sum_y=src.sum_y;
00124     sum_xx=src.sum_xx;
00125     sum_yy=src.sum_yy;
00126     sum_xy=src.sum_xy;
00127     
00128     length=src.length;
00129     target_length=src.target_length;
00130     amount++;
00131     owner=src.owner;
00132     
00133     chem = new double[par.n_chem];
00134     for (int ch=0;ch<par.n_chem;ch++)
00135       chem[ch]=src.chem[ch];
00136     
00137     return *this;
00138 
00139   }
00140 
00142   inline bool AliveP(void) const {
00143     return alive;
00144   }
00145   
00147   inline int Colour(void) const {
00148    
00149     /* if (par.dynamicJ) 
00150       return colour;
00151       else */
00152       return tau+1;
00153     
00154   };
00155 
00157   inline void setTau(int settau) {
00158     tau=settau;
00159   }
00160   
00162   inline int getTau(void) {
00163     return tau;
00164   }
00166   inline int SetColour(const int new_colour) {
00167     return colour=new_colour;
00168   }
00169 
00170   /* \brief Returns the energy between this cell and cell2. 
00171 
00172   Called from CellularPotts::DeltaH.
00173   **/
00174   int EnergyDifference(const Cell &cell2) const;
00175 
00177   inline int Area() const {
00178     return area;
00179   }
00180   
00182   inline int TargetArea() const {
00183     return target_area;
00184   }
00185   
00190   inline double TargetLength() const {
00191     return target_length;
00192   }
00193 
00195   inline double SetTargetLength(double l) {
00196     return target_length=l;
00197   }
00198   
00199 
00201   inline void PrintInertia(void) {
00202     
00203     double ixx=(double)sum_xx-(double)sum_x*sum_x/(double)area;
00204     double iyy=(double)sum_yy-(double)sum_y*sum_y/(double)area;
00205     double ixy=(double)sum_xy-(double)sum_x*sum_y/(double)area;
00206 
00207     cerr << "ixx = " << ixx << "\n";
00208     cerr << "iyy = " << iyy << "\n";
00209     cerr << "ixy = " << ixy << "\n";
00210     
00211   }
00212 
00213   // return the current length
00214   inline double Length(void) {
00215     return length;
00216   }
00217 
00225   static void ClearJ(void);
00226   double polarvec[9]; 
00227   void RenormPolarVec(void);
00228   
00233   static inline int MaxSigma() {
00234     return maxsigma;
00235   }
00236 
00238   inline int Sigma() const {
00239     return sigma;
00240   }
00241   
00243   inline int SetTargetArea(const int new_area) {
00244     return target_area=new_area;
00245   }
00246   
00248   inline void Apoptose() {
00249     alive=false;
00250   }
00251   
00253   inline int IncrementTargetArea() {
00254     return ++target_area;
00255   }
00256   
00258   inline int DecrementTargetArea() {
00259     return --target_area;
00260   }
00261 
00263   inline int Mother(void) const { return mother; }
00264   
00266   inline int Daughter(void) const { return daughter; }
00267   
00269   inline int TimesDivided(void) const { return times_divided; }
00270   
00272   inline int DateOfBirth(void) const { return date_of_birth; }
00273   
00275   inline int ColourOfBirth(void) const { return colour_of_birth; }
00276 
00278   inline int GetJ(const Cell &c2) const {
00279     return J[sigma][c2.sigma];
00280   }
00281 
00282   
00284   inline static int SetJ(int t1,int t2, int val) {
00285     return J[t2][t1]=J[t1][t2]=val;
00286   }
00287 
00288 
00289   // Deal with gradient measurements:
00290 
00292   inline double* SetGrad(double *g) {
00293     grad[0]=g[0];
00294     grad[1]=g[1];
00295     return grad;
00296   }
00297   
00299   inline const double* GetGrad(void) const {
00300     return grad;
00301   } 
00302   
00304   inline const double GradX() const {
00305     return grad[0];
00306   }
00307 
00309   inline const double GradY() const {
00310     return grad[1];
00311   }
00312 
00314   inline double* AddToGrad(double *g) {
00315     grad[0]+=g[0];
00316     grad[1]+=g[1];
00317     return grad;
00318   }
00319    
00321   inline void ClearGrad(void) {
00322     grad[0]=0.;
00323     grad[1]=0.;
00324   }  
00325   
00329   void MeasureCellSize(Cell &c);
00330 
00331 private:
00336   static void ReadStaticJTable(const char *fname);
00337 
00338   // used internally by dish in "CellGrowthAndDivision"
00339   inline int GrowthThreshold(void) const { return growth_threshold; }
00340   
00341   // used internally by class CellularPotts
00342   inline void CleanMoments(void) {
00343     sum_x = sum_y = sum_xx = sum_xy = sum_yy = area = target_area  = 0;
00344   }
00345   // used internally by class CellularPotts
00346   inline double AddSiteToMoments(int x,int y, double new_l=-1. ) {
00347     
00348     // Add a site to the raw moments, then update and return the
00349     // length of the cell
00350     
00351     // sum_x, sum_y, sum_xx, sum_xy and sum_yy are adjusted
00352     // Eventually this function may be used to carry
00353     // out all necessary adminstration at once
00354     sum_x+=x;
00355     sum_y+=y;
00356     sum_xx+=x*x;
00357     sum_yy+=y*y;
00358     sum_xy+=x*y;
00359     
00360     // update length (see appendix. A, Zajac.jtb03), if length is not given
00361     // NB. 24 NOV 2004. Found mistake in Zajac's paper. See remarks in
00362     // method "Length(..)". 
00363     if (new_l<0.) {
00364       length=Length(sum_x,sum_y,sum_xx,sum_yy,sum_xy,area);
00365     } else {
00366       length=new_l;
00367     }
00368     return length;
00369   }
00370 
00371   // used internally by class CellularPotts
00372   inline double RemoveSiteFromMoments(int x,int y, double new_l=-1.) {
00373     
00374     // Remove a site from the raw moments, then update and return the
00375     // length of the cell
00376     
00377     // sum_x, sum_y, sum_xx, sum_xy and sum_yy are adjusted
00378     // Eventually this function may be used to carry
00379     // out all necessary adminstration at once
00380     sum_x-=x;
00381     sum_y-=y;
00382     sum_xx-=x*x;
00383     sum_yy-=y*y;
00384     sum_xy-=x*y;
00385     
00386     // update length (see app. A, Zajac.jtb03), if length is not given
00387     if (new_l<0.) {
00388       length=Length(sum_x,sum_y,sum_xx,sum_yy,sum_xy,area);
00389     } else {
00390       length=new_l;
00391     }
00392     return length;
00393   }
00394 
00396   //components (used internally)
00397   inline double Length(int s_x,int s_y,int s_xx,
00398                                 int s_yy,int s_xy,int n) {
00399     
00400     // inertia tensor (constructed from the raw momenta, see notebook)
00401     double iyy=(double)s_xx-(double)s_x*s_x/(double)n;
00402     double ixx=(double)s_yy-(double)s_y*s_y/(double)n;
00403     double ixy=-(double)s_xy+(double)s_x*s_y/(double)n;
00404         
00405     double rhs1=(ixx+iyy)/2., rhs2=sqrt( (ixx-iyy)*(ixx-iyy)+4*ixy*ixy )/2.;
00406 
00407     double lambda_b=rhs1+rhs2;
00408     //double lambda_a=rhs1-rhs2;
00409     
00410     // according to Zajac et al. 2003:
00411     //return 2*sqrt(lambda_b);
00412     // Grumble, this is not right!!!
00413     // Must divide by mass!!!!!!
00414 
00415     // see: http://scienceworld.wolfram.com/physics/MomentofInertiaEllipse.html
00416     //    cerr << "n = " << n << "\n";
00417     return 4*sqrt(lambda_b/n);
00418 
00419     // 2*sqrt(lambda_b/n) give semimajor axis. We want the length.
00420 
00421   }
00422   
00423   // return the new length that the cell would have
00424   // if site (x,y) were added.
00425   // used internally by CellularPotts
00426   inline double GetNewLengthIfXYWereAdded(int x, int y) {
00427     
00428     return Length(sum_x+x,sum_y+y,
00429                            sum_xx+x*x,sum_yy+y*y,sum_xy+x*y,area+1);
00430     
00431   }
00432 
00433   // return the new length that the cell would have
00434   // if site (x,y) were removed
00435   // used internally by CellularPotts
00436   inline double GetNewLengthIfXYWereRemoved(int x, int y) {
00437 
00438         return Length(sum_x-x,sum_y-y,
00439                                sum_xx-x*x,sum_yy-y*y,sum_xy-x*y,area-1);
00440 
00441   }
00442 
00443   
00444 private:
00446   inline int IncrementArea() {
00447     return ++area;
00448   }
00449 
00451   inline int DecrementArea() {
00452     return --area;
00453   }
00454 
00455   
00460   inline int SetAreaToTarget(void) {
00461     return area=target_area;
00462   }
00463 
00465   void ConstructorBody(int settau=1);
00466   
00467   // returns the maximum cell type index
00468   // (depends on Jtable)
00469   static int MaxTau(void) {
00470     return maxtau;
00471   }
00472 
00473 protected:
00474   int colour;
00475   bool alive;
00476   int sigma; // cell identity, 0 if medium
00477   int tau; // Cell type, when dynamicJ's are not used
00478 
00479   // Two dimensional (square) array of ints, containing the J's.
00480   double length; // length of the cell;
00481   double target_length;
00482 
00483   // Dynamically increased when cells are added to the system
00484   // unless a static Jtable is used (currently this is the default situation)
00485   static int  **J;
00486   static int maxtau;
00487 
00488   // Amount: the number of Cell instantations, INCLUDING copies
00489   // For internal use only.
00490   // Reading amount is NOT the way to get the number of cells!!
00491   static int amount;
00492   static int capacity;
00493   static int maxsigma; // the last cell identity number given out
00494   
00495 
00496   // indices of mother and daughter
00497   // (Note: no pointers, cells may be relocated)
00498   int mother;
00499   int daughter;
00500   int times_divided;
00501   int date_of_birth;
00502   int colour_of_birth;
00503   
00504   int area;
00505   int target_area;
00506   int growth_threshold;
00507 
00508   double v[2];
00509   int n_copies; // number of expansions of this cell
00510   // gradient of a chemical (to be extended to the total number chemicals)
00511   double grad[2];
00512   
00513   double *chem;
00514   // Raw moments of the cells
00515   // Are used to calculate minor and major axes
00516   // and center of mass
00517   // are locally adjusted, so axes are easily
00518   // and quickly calculated!
00519   
00520   // N.B: N is area!
00521   
00522   int sum_x;
00523   int sum_y;
00524   int sum_xx;
00525   int sum_yy;
00526   int sum_xy;
00527   
00528   const Dish *owner; // pointer to owner of cell
00529 
00530 };
00531 
00532 #endif
```

---

Generated on Tue Dec 12 16:32:40 2006 for Tissue Simulation Toolkit by

1.3.5 
