# Supplementary material for: Contact-Inhibited Chemotaxis in De Novo and Sprouting Blood-Vessel Growth
Source: PLoS Comput Biol. 2008 Sep 19;4(9):e1000163. doi: 10.1371/journal.pcbi.1000163 (PMC2528254; doi:10.1371/journal.pcbi.1000163)
Supplement: Protocol S1 — Tissue Simulation Toolkit v0.1.3. The source code for the software used for the simulations presented in this paper is also available from http://sourceforge.net/projects/tst. Installation: Unpack and compile according to the instructions given in the INSTALL file The code is written in C++ using the cross-platform (Windows, Mac, or Unix/Linux) library Qt (available from www.trolltech.com). (332 KB ZIP) [file pcbi.1000163.s002.zip › TST0.1.3/html/classCell-members.html]

Tissue Simulation Toolkit: Member List

Main Page | Namespace List | Class Hierarchy | Class List | File List | Namespace Members | Class Members | File Members

# Cell Member List

This is the complete list of members for Cell, including all inherited members.

|  |  |  |
| --- | --- | --- |
| AddToGrad(double \*g) | Cell | `[inline]` |
| alive | Cell | `[protected]` |
| AliveP(void) const | Cell | `[inline]` |
| amount | Cell | `[protected, static]` |
| Apoptose() | Cell | `[inline]` |
| area | Cell | `[protected]` |
| Area() const | Cell | `[inline]` |
| capacity | Cell | `[protected, static]` |
| Cell(const Dish &who, int settau=1) | Cell | `[inline]` |
| Cell(void) | Cell | `[inline]` |
| Cell(const Cell &src) | Cell | `[inline]` |
| Cell(Cell &mother, int settau=1) | Cell |  |
| CellularPotts class | Cell | `[friend]` |
| chem | Cell | `[protected]` |
| ClearGrad(void) | Cell | `[inline]` |
| ClearJ(void) | Cell | `[static]` |
| Colour(void) const | Cell | `[inline]` |
| colour | Cell | `[protected]` |
| colour\_of\_birth | Cell | `[protected]` |
| ColourOfBirth(void) const | Cell | `[inline]` |
| date\_of\_birth | Cell | `[protected]` |
| DateOfBirth(void) const | Cell | `[inline]` |
| daughter | Cell | `[protected]` |
| Daughter(void) const | Cell | `[inline]` |
| DecrementTargetArea() | Cell | `[inline]` |
| Dish class | Cell | `[friend]` |
| EnergyDifference(const Cell &cell2) const | Cell |  |
| GetGrad(void) const | Cell | `[inline]` |
| GetJ(const Cell &c2) const | Cell | `[inline]` |
| getTau(void) | Cell | `[inline]` |
| grad | Cell | `[protected]` |
| GradX() const | Cell | `[inline]` |
| GradY() const | Cell | `[inline]` |
| growth\_threshold | Cell | `[protected]` |
| IncrementTargetArea() | Cell | `[inline]` |
| Info class | Cell | `[friend]` |
| J | Cell | `[protected, static]` |
| length | Cell | `[protected]` |
| Length(void) | Cell | `[inline]` |
| MaxSigma() | Cell | `[inline, static]` |
| maxsigma | Cell | `[protected, static]` |
| maxtau | Cell | `[protected, static]` |
| MeasureCellSize(Cell &c) | Cell |  |
| mother | Cell | `[protected]` |
| Mother(void) const | Cell | `[inline]` |
| n\_copies | Cell | `[protected]` |
| operator=(const Cell &src) | Cell | `[inline]` |
| owner | Cell | `[protected]` |
| polarvec | Cell |  |
| PrintInertia(void) | Cell | `[inline]` |
| RenormPolarVec(void) | Cell |  |
| SetColour(const int new\_colour) | Cell | `[inline]` |
| SetGrad(double \*g) | Cell | `[inline]` |
| SetJ(int t1, int t2, int val) | Cell | `[inline, static]` |
| SetTargetArea(const int new\_area) | Cell | `[inline]` |
| SetTargetLength(double l) | Cell | `[inline]` |
| setTau(int settau) | Cell | `[inline]` |
| sigma | Cell | `[protected]` |
| Sigma() const | Cell | `[inline]` |
| sum\_x | Cell | `[protected]` |
| sum\_xx | Cell | `[protected]` |
| sum\_xy | Cell | `[protected]` |
| sum\_y | Cell | `[protected]` |
| sum\_yy | Cell | `[protected]` |
| target\_area | Cell | `[protected]` |
| target\_length | Cell | `[protected]` |
| TargetArea() const | Cell | `[inline]` |
| TargetLength() const | Cell | `[inline]` |
| tau | Cell | `[protected]` |
| times\_divided | Cell | `[protected]` |
| TimesDivided(void) const | Cell | `[inline]` |
| v | Cell | `[protected]` |
| ~Cell(void) | Cell |  |

---

Generated on Tue Dec 12 16:32:41 2006 for Tissue Simulation Toolkit by

1.3.5 
