# Supplementary material for: Contact-Inhibited Chemotaxis in De Novo and Sprouting Blood-Vessel Growth
Source: PLoS Comput Biol. 2008 Sep 19;4(9):e1000163. doi: 10.1371/journal.pcbi.1000163 (PMC2528254; doi:10.1371/journal.pcbi.1000163)
Supplement: Protocol S1 — Tissue Simulation Toolkit v0.1.3. The source code for the software used for the simulations presented in this paper is also available from http://sourceforge.net/projects/tst. Installation: Unpack and compile according to the instructions given in the INSTALL file The code is written in C++ using the cross-platform (Windows, Mac, or Unix/Linux) library Qt (available from www.trolltech.com). (332 KB ZIP) [file pcbi.1000163.s002.zip › TST0.1.3/html/classCell.html]

Tissue Simulation Toolkit: Cell class Reference

Main Page | Namespace List | Class Hierarchy | Class List | File List | Namespace Members | Class Members | File Members

# Cell Class Reference

`#include <cell.h>`

List of all members.

|  |
| --- |
|  |
| Public Member Functions | |
|  | Cell (const Dish &who, int settau=1) |
|  | Constructor to insert a cell into Dish "who". |
|  | Cell (void) |
|  | ~Cell (void) |
|  | Cell (const Cell &src) |
|  | Default copy constructor. |
|  | Cell (Cell &mother, int settau=1) |
|  | Special copy constructor to add a new cell to the dish. |
| Cell & | operator= (const Cell &src) |
|  | Assignment operator. |
| bool | AliveP (void) const |
|  | Returns false if Cell has apoptosed (vanished). |
| int | Colour (void) const |
|  | Returns the cell colour. |
| void | setTau (int settau) |
|  | Set cell type of this Cell. |
| int | getTau (void) |
|  | Get cell type of this Cell. |
| int | SetColour (const int new\_colour) |
|  | Set color of this cell to new\_colour, irrespective of type. |
| int | EnergyDifference (const Cell &cell2) const |
| int | Area () const |
|  | Return Cell's actual area. |
| int | TargetArea () const |
|  | Return Cell's target area. |
| double | TargetLength () const |
|  | Return Cell's target length. |
| double | SetTargetLength (double l) |
|  | Set the Cell's target length. |
| void | PrintInertia (void) |
|  | Debugging function used to print the cell's current inertia tensor (as used for calculations of the length ). |
| double | Length (void) |
| void | RenormPolarVec (void) |
| int | Sigma () const |
|  | Returns the cell's cell identity number. |
| int | SetTargetArea (const int new\_area) |
|  | Sets the target area of the cell. |
| void | Apoptose () |
|  | Sends the current cell into apoptosis. |
| int | IncrementTargetArea () |
|  | Decrement the cell's target area by one unit. |
| int | DecrementTargetArea () |
|  | Increment the cell's target area by one unit. |
| int | Mother (void) const |
|  | Cell lineage tracking: get the cell's parent. |
| int | Daughter (void) const |
|  | Cell lineage tracking: get the cell's daughter. |
| int | TimesDivided (void) const |
|  | Returns a counter keeping track of the number of divisions. |
| int | DateOfBirth (void) const |
|  | Returns Monte Carlo Step (MCS) when this cell originated. |
| int | ColourOfBirth (void) const |
|  | Returns the cell type at the time of birth. |
| int | GetJ (const Cell &c2) const |
|  | Returns the bond energy J between this cell and cell c2. |
| double \* | SetGrad (double \*g) |
|  | Set the current gradient of the cell to g. Currently not in use. |
| const double \* | GetGrad (void) const |
|  | Returns the cell's measured gradient. Currently not in use. |
| const double | GradX () const |
|  | Returns the cell's measured gradient. Currently not in use. |
| const double | GradY () const |
|  | Returns the cell's measured gradient. Currently not in use. |
| double \* | AddToGrad (double \*g) |
|  | Currently not in use (remove?). |
| void | ClearGrad (void) |
|  | Currently not in use (remove?). |
| void | MeasureCellSize (Cell &c) |
| Static Public Member Functions | |
| void | ClearJ (void) |
|  | Clears the table of J's. |
| int | MaxSigma () |
|  | Returns the maximum cell identity number in the Dish. This would normally be the number of cells in the Dish, although the number includes apoptosed cells. |
| int | SetJ (int t1, int t2, int val) |
|  | Sets bond energy J between cell type t1 and t2 to val. |
| Public Attributes | |
| double | polarvec [9] |
| Protected Attributes | |
| int | colour |
| bool | alive |
| int | sigma |
| int | tau |
| double | length |
| double | target\_length |
| int | mother |
| int | daughter |
| int | times\_divided |
| int | date\_of\_birth |
| int | colour\_of\_birth |
| int | area |
| int | target\_area |
| int | growth\_threshold |
| double | v [2] |
| int | n\_copies |
| double | grad [2] |
| double \* | chem |
| int | sum\_x |
| int | sum\_y |
| int | sum\_xx |
| int | sum\_yy |
| int | sum\_xy |
| const Dish \* | owner |
| Static Protected Attributes | |
| int \*\* | J = 0 |
| int | maxtau = 0 |
| int | amount = 0 |
| int | capacity = 0 |
| int | maxsigma = 0 |
| Friends | |
| class | Dish |
| class | CellularPotts |
| class | Info |

---

## Constructor & Destructor Documentation

|  |  |  |  |  |  |  |  |  |  |  |  |  |
| --- | --- | --- | --- | --- | --- | --- | --- | --- | --- | --- | --- | --- |
| |  |  |  |  | | --- | --- | --- | --- | | Cell::Cell | ( | const Dish & | *who*, | |  |  | int | *settau* = 1 | |  | ) | `[inline]` | | |

|  |  |
| --- | --- |
|  | Constructor to insert a cell into Dish "who". Used to add a new Cell to the dish: new Cell(dish, celtype). |

|  |  |  |  |  |  |  |
| --- | --- | --- | --- | --- | --- | --- |
| |  |  |  |  |  |  | | --- | --- | --- | --- | --- | --- | | Cell::Cell | ( | void |  | ) | `[inline]` | |

|  |  |
| --- | --- |
|  |  |

|  |  |  |  |  |  |  |
| --- | --- | --- | --- | --- | --- | --- |
| |  |  |  |  |  |  | | --- | --- | --- | --- | --- | --- | | Cell::~Cell | ( | void |  | ) |  | |

|  |  |
| --- | --- |
|  |  |

|  |  |  |  |  |  |  |
| --- | --- | --- | --- | --- | --- | --- |
| |  |  |  |  |  |  | | --- | --- | --- | --- | --- | --- | | Cell::Cell | ( | const Cell & | *src* | ) | `[inline]` | |

|  |  |
| --- | --- |
|  | Default copy constructor. |

|  |  |  |  |  |  |  |  |  |  |  |  |  |
| --- | --- | --- | --- | --- | --- | --- | --- | --- | --- | --- | --- | --- |
| |  |  |  |  | | --- | --- | --- | --- | | Cell::Cell | ( | Cell & | *mother*, | |  |  | int | *settau* = 1 | |  | ) |  | | |

|  |  |  |  |
| --- | --- | --- | --- |
|  | Special copy constructor to add a new cell to the dish. Call it as: new Cell(parent, true); mother will be modified for ancestry administration! **Parameters:**  |  |  | | --- | --- | | *settau* | Cell type of daughter cell. | |

---

## Member Function Documentation

|  |  |  |  |  |  |  |
| --- | --- | --- | --- | --- | --- | --- |
| |  |  |  |  |  |  | | --- | --- | --- | --- | --- | --- | | double\* Cell::AddToGrad | ( | double \* | *g* | ) | `[inline]` | |

|  |  |
| --- | --- |
|  | Currently not in use (remove?). |

|  |  |  |  |  |  |  |
| --- | --- | --- | --- | --- | --- | --- |
| |  |  |  |  |  |  | | --- | --- | --- | --- | --- | --- | | bool Cell::AliveP | ( | void |  | ) | const `[inline]` | |

|  |  |
| --- | --- |
|  | Returns false if Cell has apoptosed (vanished). |

|  |  |  |  |  |  |
| --- | --- | --- | --- | --- | --- |
| |  |  |  |  |  | | --- | --- | --- | --- | --- | | void Cell::Apoptose | ( |  | ) | `[inline]` | |

|  |  |
| --- | --- |
|  | Sends the current cell into apoptosis. |

|  |  |  |  |  |  |
| --- | --- | --- | --- | --- | --- |
| |  |  |  |  |  | | --- | --- | --- | --- | --- | | int Cell::Area | ( |  | ) | const `[inline]` | |

|  |  |
| --- | --- |
|  | Return Cell's actual area. |

|  |  |  |  |  |  |  |
| --- | --- | --- | --- | --- | --- | --- |
| |  |  |  |  |  |  | | --- | --- | --- | --- | --- | --- | | void Cell::ClearGrad | ( | void |  | ) | `[inline]` | |

|  |  |
| --- | --- |
|  | Currently not in use (remove?). |

|  |  |  |  |  |  |  |
| --- | --- | --- | --- | --- | --- | --- |
| |  |  |  |  |  |  | | --- | --- | --- | --- | --- | --- | | void Cell::ClearJ | ( | void |  | ) | `[static]` | |

|  |  |
| --- | --- |
|  | Clears the table of J's. This is only important for a feature called "DynamicJ's", where J-values depend on internal states of the cells (such as a genetic network; see e.g. Hogeweg et al. 2000). The current version of TST does not include such functionality. |

|  |  |  |  |  |  |  |
| --- | --- | --- | --- | --- | --- | --- |
| |  |  |  |  |  |  | | --- | --- | --- | --- | --- | --- | | int Cell::Colour | ( | void |  | ) | const `[inline]` | |

|  |  |
| --- | --- |
|  | Returns the cell colour. |

|  |  |  |  |  |  |  |
| --- | --- | --- | --- | --- | --- | --- |
| |  |  |  |  |  |  | | --- | --- | --- | --- | --- | --- | | int Cell::ColourOfBirth | ( | void |  | ) | const `[inline]` | |

|  |  |
| --- | --- |
|  | Returns the cell type at the time of birth. |

|  |  |  |  |  |  |  |
| --- | --- | --- | --- | --- | --- | --- |
| |  |  |  |  |  |  | | --- | --- | --- | --- | --- | --- | | int Cell::DateOfBirth | ( | void |  | ) | const `[inline]` | |

|  |  |
| --- | --- |
|  | Returns Monte Carlo Step (MCS) when this cell originated. |

|  |  |  |  |  |  |  |
| --- | --- | --- | --- | --- | --- | --- |
| |  |  |  |  |  |  | | --- | --- | --- | --- | --- | --- | | int Cell::Daughter | ( | void |  | ) | const `[inline]` | |

|  |  |
| --- | --- |
|  | Cell lineage tracking: get the cell's daughter. |

|  |  |  |  |  |  |
| --- | --- | --- | --- | --- | --- |
| |  |  |  |  |  | | --- | --- | --- | --- | --- | | int Cell::DecrementTargetArea | ( |  | ) | `[inline]` | |

|  |  |
| --- | --- |
|  | Increment the cell's target area by one unit. |

|  |  |  |  |  |  |  |
| --- | --- | --- | --- | --- | --- | --- |
| |  |  |  |  |  |  | | --- | --- | --- | --- | --- | --- | | int Cell::EnergyDifference | ( | const Cell & | *cell2* | ) | const | |

|  |  |
| --- | --- |
|  |  |

|  |  |  |  |  |  |  |
| --- | --- | --- | --- | --- | --- | --- |
| |  |  |  |  |  |  | | --- | --- | --- | --- | --- | --- | | const double\* Cell::GetGrad | ( | void |  | ) | const `[inline]` | |

|  |  |
| --- | --- |
|  | Returns the cell's measured gradient. Currently not in use. |

|  |  |  |  |  |  |  |
| --- | --- | --- | --- | --- | --- | --- |
| |  |  |  |  |  |  | | --- | --- | --- | --- | --- | --- | | int Cell::GetJ | ( | const Cell & | *c2* | ) | const `[inline]` | |

|  |  |
| --- | --- |
|  | Returns the bond energy J between this cell and cell c2. |

|  |  |  |  |  |  |  |
| --- | --- | --- | --- | --- | --- | --- |
| |  |  |  |  |  |  | | --- | --- | --- | --- | --- | --- | | int Cell::getTau | ( | void |  | ) | `[inline]` | |

|  |  |
| --- | --- |
|  | Get cell type of this Cell. |

|  |  |  |  |  |  |
| --- | --- | --- | --- | --- | --- |
| |  |  |  |  |  | | --- | --- | --- | --- | --- | | const double Cell::GradX | ( |  | ) | const `[inline]` | |

|  |  |
| --- | --- |
|  | Returns the cell's measured gradient. Currently not in use. |

|  |  |  |  |  |  |
| --- | --- | --- | --- | --- | --- |
| |  |  |  |  |  | | --- | --- | --- | --- | --- | | const double Cell::GradY | ( |  | ) | const `[inline]` | |

|  |  |
| --- | --- |
|  | Returns the cell's measured gradient. Currently not in use. |

|  |  |  |  |  |  |
| --- | --- | --- | --- | --- | --- |
| |  |  |  |  |  | | --- | --- | --- | --- | --- | | int Cell::IncrementTargetArea | ( |  | ) | `[inline]` | |

|  |  |
| --- | --- |
|  | Decrement the cell's target area by one unit. |

|  |  |  |  |  |  |  |
| --- | --- | --- | --- | --- | --- | --- |
| |  |  |  |  |  |  | | --- | --- | --- | --- | --- | --- | | double Cell::Length | ( | void |  | ) | `[inline]` | |

|  |  |
| --- | --- |
|  |  |

|  |  |  |  |  |  |
| --- | --- | --- | --- | --- | --- |
| |  |  |  |  |  | | --- | --- | --- | --- | --- | | int Cell::MaxSigma | ( |  | ) | `[inline, static]` | |

|  |  |
| --- | --- |
|  | Returns the maximum cell identity number in the Dish. This would normally be the number of cells in the Dish, although the number includes apoptosed cells. |

|  |  |  |  |  |  |  |
| --- | --- | --- | --- | --- | --- | --- |
| |  |  |  |  |  |  | | --- | --- | --- | --- | --- | --- | | void Cell::MeasureCellSize | ( | Cell & | *c* | ) |  | |

|  |  |
| --- | --- |
|  | After introducing a new Cell (e.g. with GrowInCell) call this function to set the moments and areas right. |

|  |  |  |  |  |  |  |
| --- | --- | --- | --- | --- | --- | --- |
| |  |  |  |  |  |  | | --- | --- | --- | --- | --- | --- | | int Cell::Mother | ( | void |  | ) | const `[inline]` | |

|  |  |
| --- | --- |
|  | Cell lineage tracking: get the cell's parent. |

|  |  |  |  |  |  |  |
| --- | --- | --- | --- | --- | --- | --- |
| |  |  |  |  |  |  | | --- | --- | --- | --- | --- | --- | | Cell& Cell::operator= | ( | const Cell & | *src* | ) | `[inline]` | |

|  |  |
| --- | --- |
|  | Assignment operator. Called if one cell is assigned to another. Remember to change both assignment operator and copy constructor when adding new attributes to Cell. |

|  |  |  |  |  |  |  |
| --- | --- | --- | --- | --- | --- | --- |
| |  |  |  |  |  |  | | --- | --- | --- | --- | --- | --- | | void Cell::PrintInertia | ( | void |  | ) | `[inline]` | |

|  |  |
| --- | --- |
|  | Debugging function used to print the cell's current inertia tensor (as used for calculations of the length ). |

|  |  |  |  |  |  |  |
| --- | --- | --- | --- | --- | --- | --- |
| |  |  |  |  |  |  | | --- | --- | --- | --- | --- | --- | | void Cell::RenormPolarVec | ( | void |  | ) |  | |

|  |  |
| --- | --- |
|  |  |

|  |  |  |  |  |  |  |
| --- | --- | --- | --- | --- | --- | --- |
| |  |  |  |  |  |  | | --- | --- | --- | --- | --- | --- | | int Cell::SetColour | ( | const int | *new\_colour* | ) | `[inline]` | |

|  |  |
| --- | --- |
|  | Set color of this cell to new\_colour, irrespective of type. |

|  |  |  |  |  |  |  |
| --- | --- | --- | --- | --- | --- | --- |
| |  |  |  |  |  |  | | --- | --- | --- | --- | --- | --- | | double\* Cell::SetGrad | ( | double \* | *g* | ) | `[inline]` | |

|  |  |
| --- | --- |
|  | Set the current gradient of the cell to g. Currently not in use. |

|  |  |  |  |  |  |  |  |  |  |  |  |  |  |  |  |  |
| --- | --- | --- | --- | --- | --- | --- | --- | --- | --- | --- | --- | --- | --- | --- | --- | --- |
| |  |  |  |  | | --- | --- | --- | --- | | int Cell::SetJ | ( | int | *t1*, | |  |  | int | *t2*, | |  |  | int | *val* | |  | ) | `[inline, static]` | | |

|  |  |
| --- | --- |
|  | Sets bond energy J between cell type t1 and t2 to val. |

|  |  |  |  |  |  |  |
| --- | --- | --- | --- | --- | --- | --- |
| |  |  |  |  |  |  | | --- | --- | --- | --- | --- | --- | | int Cell::SetTargetArea | ( | const int | *new\_area* | ) | `[inline]` | |

|  |  |
| --- | --- |
|  | Sets the target area of the cell. |

|  |  |  |  |  |  |  |
| --- | --- | --- | --- | --- | --- | --- |
| |  |  |  |  |  |  | | --- | --- | --- | --- | --- | --- | | double Cell::SetTargetLength | ( | double | *l* | ) | `[inline]` | |

|  |  |
| --- | --- |
|  | Set the Cell's target length. |

|  |  |  |  |  |  |  |
| --- | --- | --- | --- | --- | --- | --- |
| |  |  |  |  |  |  | | --- | --- | --- | --- | --- | --- | | void Cell::setTau | ( | int | *settau* | ) | `[inline]` | |

|  |  |
| --- | --- |
|  | Set cell type of this Cell. |

|  |  |  |  |  |  |
| --- | --- | --- | --- | --- | --- |
| |  |  |  |  |  | | --- | --- | --- | --- | --- | | int Cell::Sigma | ( |  | ) | const `[inline]` | |

|  |  |
| --- | --- |
|  | Returns the cell's cell identity number. |

|  |  |  |  |  |  |
| --- | --- | --- | --- | --- | --- |
| |  |  |  |  |  | | --- | --- | --- | --- | --- | | int Cell::TargetArea | ( |  | ) | const `[inline]` | |

|  |  |
| --- | --- |
|  | Return Cell's target area. |

|  |  |  |  |  |  |
| --- | --- | --- | --- | --- | --- |
| |  |  |  |  |  | | --- | --- | --- | --- | --- | | double Cell::TargetLength | ( |  | ) | const `[inline]` | |

|  |  |
| --- | --- |
|  | Return Cell's target length. Length constraint is documented in Merks et al. 2006, Dev. Biol. |

|  |  |  |  |  |  |  |
| --- | --- | --- | --- | --- | --- | --- |
| |  |  |  |  |  |  | | --- | --- | --- | --- | --- | --- | | int Cell::TimesDivided | ( | void |  | ) | const `[inline]` | |

|  |  |
| --- | --- |
|  | Returns a counter keeping track of the number of divisions. |

---

## Friends And Related Function Documentation

|  |  |
| --- | --- |
| |  | | --- | | friend class CellularPotts `[friend]` | |

|  |  |
| --- | --- |
|  |  |

|  |  |
| --- | --- |
| |  | | --- | | friend class Dish `[friend]` | |

|  |  |
| --- | --- |
|  |  |

|  |  |
| --- | --- |
| |  | | --- | | friend class Info `[friend]` | |

|  |  |
| --- | --- |
|  |  |

---

## Member Data Documentation

|  |  |
| --- | --- |
| |  | | --- | | bool Cell::alive `[protected]` | |

|  |  |
| --- | --- |
|  |  |

|  |  |
| --- | --- |
| |  | | --- | | int Cell::amount = 0 `[static, protected]` | |

|  |  |
| --- | --- |
|  |  |

|  |  |
| --- | --- |
| |  | | --- | | int Cell::area `[protected]` | |

|  |  |
| --- | --- |
|  |  |

|  |  |
| --- | --- |
| |  | | --- | | int Cell::capacity = 0 `[static, protected]` | |

|  |  |
| --- | --- |
|  |  |

|  |  |
| --- | --- |
| |  | | --- | | double\* Cell::chem `[protected]` | |

|  |  |
| --- | --- |
|  |  |

|  |  |
| --- | --- |
| |  | | --- | | int Cell::colour `[protected]` | |

|  |  |
| --- | --- |
|  |  |

|  |  |
| --- | --- |
| |  | | --- | | int Cell::colour\_of\_birth `[protected]` | |

|  |  |
| --- | --- |
|  |  |

|  |  |
| --- | --- |
| |  | | --- | | int Cell::date\_of\_birth `[protected]` | |

|  |  |
| --- | --- |
|  |  |

|  |  |
| --- | --- |
| |  | | --- | | int Cell::daughter `[protected]` | |

|  |  |
| --- | --- |
|  |  |

|  |  |
| --- | --- |
| |  | | --- | | double Cell::grad[2] `[protected]` | |

|  |  |
| --- | --- |
|  |  |

|  |  |
| --- | --- |
| |  | | --- | | int Cell::growth\_threshold `[protected]` | |

|  |  |
| --- | --- |
|  |  |

|  |  |
| --- | --- |
| |  | | --- | | int \*\* Cell::J = 0 `[static, protected]` | |

|  |  |
| --- | --- |
|  |  |

|  |  |
| --- | --- |
| |  | | --- | | double Cell::length `[protected]` | |

|  |  |
| --- | --- |
|  |  |

|  |  |
| --- | --- |
| |  | | --- | | int Cell::maxsigma = 0 `[static, protected]` | |

|  |  |
| --- | --- |
|  |  |

|  |  |
| --- | --- |
| |  | | --- | | int Cell::maxtau = 0 `[static, protected]` | |

|  |  |
| --- | --- |
|  |  |

|  |  |
| --- | --- |
| |  | | --- | | int Cell::mother `[protected]` | |

|  |  |
| --- | --- |
|  |  |

|  |  |
| --- | --- |
| |  | | --- | | int Cell::n\_copies `[protected]` | |

|  |  |
| --- | --- |
|  |  |

|  |  |
| --- | --- |
| |  | | --- | | const Dish\* Cell::owner `[protected]` | |

|  |  |
| --- | --- |
|  |  |

|  |  |
| --- | --- |
| |  | | --- | | double Cell::polarvec[9] | |

|  |  |
| --- | --- |
|  |  |

|  |  |
| --- | --- |
| |  | | --- | | int Cell::sigma `[protected]` | |

|  |  |
| --- | --- |
|  |  |

|  |  |
| --- | --- |
| |  | | --- | | int Cell::sum\_x `[protected]` | |

|  |  |
| --- | --- |
|  |  |

|  |  |
| --- | --- |
| |  | | --- | | int Cell::sum\_xx `[protected]` | |

|  |  |
| --- | --- |
|  |  |

|  |  |
| --- | --- |
| |  | | --- | | int Cell::sum\_xy `[protected]` | |

|  |  |
| --- | --- |
|  |  |

|  |  |
| --- | --- |
| |  | | --- | | int Cell::sum\_y `[protected]` | |

|  |  |
| --- | --- |
|  |  |

|  |  |
| --- | --- |
| |  | | --- | | int Cell::sum\_yy `[protected]` | |

|  |  |
| --- | --- |
|  |  |

|  |  |
| --- | --- |
| |  | | --- | | int Cell::target\_area `[protected]` | |

|  |  |
| --- | --- |
|  |  |

|  |  |
| --- | --- |
| |  | | --- | | double Cell::target\_length `[protected]` | |

|  |  |
| --- | --- |
|  |  |

|  |  |
| --- | --- |
| |  | | --- | | int Cell::tau `[protected]` | |

|  |  |
| --- | --- |
|  |  |

|  |  |
| --- | --- |
| |  | | --- | | int Cell::times\_divided `[protected]` | |

|  |  |
| --- | --- |
|  |  |

|  |  |
| --- | --- |
| |  | | --- | | double Cell::v[2] `[protected]` | |

|  |  |
| --- | --- |
|  |  |

---

The documentation for this class was generated from the following files:

- /home/romer/TST0.1.3/cell.h- /home/romer/TST0.1.3/cell.cpp

---

Generated on Tue Dec 12 16:32:41 2006 for Tissue Simulation Toolkit by

1.3.5
