# Supplementary material for: Contact-Inhibited Chemotaxis in De Novo and Sprouting Blood-Vessel Growth
Source: PLoS Comput Biol. 2008 Sep 19;4(9):e1000163. doi: 10.1371/journal.pcbi.1000163 (PMC2528254; doi:10.1371/journal.pcbi.1000163)
Supplement: Protocol S1 — Tissue Simulation Toolkit v0.1.3. The source code for the software used for the simulations presented in this paper is also available from http://sourceforge.net/projects/tst. Installation: Unpack and compile according to the instructions given in the INSTALL file The code is written in C++ using the cross-platform (Windows, Mac, or Unix/Linux) library Qt (available from www.trolltech.com). (332 KB ZIP) [file pcbi.1000163.s002.zip › TST0.1.3/html/classCellularPotts-members.html]

Tissue Simulation Toolkit: Member List

Main Page | Namespace List | Class Hierarchy | Class List | File List | Namespace Members | Class Members | File Members

# CellularPotts Member List

This is the complete list of members for CellularPotts, including all inherited members.

|  |  |  |
| --- | --- | --- |
| AddCell(Dish &beast) | CellularPotts | `[inline]` |
| AllocateSigma(int sx, int sy) | CellularPotts | `[virtual]` |
| AmoebaeMove(PDE \*PDEfield=0) | CellularPotts |  |
| BaseInitialisation(std::vector< Cell > \*cell) | CellularPotts | `[protected]` |
| CellDensity(void) const | CellularPotts |  |
| CellularPotts(std::vector< Cell > \*cells, const int sizex=200, const int sizey=200) | CellularPotts |  |
| CellularPotts(void) | CellularPotts |  |
| Compactness(double \*res\_compactness=0, double \*res\_area=0, double \*res\_cell\_area=0) | CellularPotts |  |
| ConstructInitCells(Dish &beast) | CellularPotts |  |
| DivideCells(void) | CellularPotts | `[inline]` |
| DivideCells(std::vector< bool > which\_cells) | CellularPotts |  |
| DrawConvexHull(Graphics \*g, int color=1) | CellularPotts |  |
| FindCellDirections(void) const | CellularPotts |  |
| getCell(int c) | CellularPotts | `[inline]` |
| GrowAndDivideCells(int growth\_rate) | CellularPotts |  |
| GrowInCells(int n\_cells, int cellsize, double subfield=1.) | CellularPotts |  |
| GrowInCells(int n\_cells, int cell\_size, int sx, int sy, int offset\_x, int offset\_y) | CellularPotts |  |
| Info class | CellularPotts | `[friend]` |
| Mass(void) | CellularPotts | `[inline]` |
| MeanCellArea(void) const | CellularPotts |  |
| Morphometry class | CellularPotts | `[friend]` |
| Plot(Graphics \*g) | CellularPotts | `[inline]` |
| PlotSigma(Graphics \*g, int mag=2) | CellularPotts |  |
| ReadZygotePicture(void) | CellularPotts |  |
| Replace(Graphics \*g) | CellularPotts |  |
| ResetTargetLengths(void) | CellularPotts |  |
| SearchNandPlot(Graphics \*g=0, bool get\_neighbours=true) | CellularPotts |  |
| SearchNeighbours(void) | CellularPotts | `[inline]` |
| SetRandomTypes(void) | CellularPotts |  |
| ShowDirections(Graphics &g, const Dir \*celldir) const | CellularPotts |  |
| sigma | CellularPotts | `[protected]` |
| Sigma(const int x, const int y) const | CellularPotts | `[inline]` |
| sizex | CellularPotts | `[protected]` |
| SizeX() const | CellularPotts | `[inline]` |
| sizey | CellularPotts | `[protected]` |
| SizeY() const | CellularPotts | `[inline]` |
| spins\_converted | CellularPotts |  |
| ThrowInCells(int n, int cellsize) | CellularPotts |  |
| Time() const | CellularPotts | `[inline]` |
| ZygoteArea() const | CellularPotts | `[inline]` |
| ~CellularPotts() | CellularPotts | `[virtual]` |

---

Generated on Tue Dec 12 16:32:41 2006 for Tissue Simulation Toolkit by

1.3.5 
