# Supplementary material for: Contact-Inhibited Chemotaxis in De Novo and Sprouting Blood-Vessel Growth
Source: PLoS Comput Biol. 2008 Sep 19;4(9):e1000163. doi: 10.1371/journal.pcbi.1000163 (PMC2528254; doi:10.1371/journal.pcbi.1000163)
Supplement: Protocol S1 — Tissue Simulation Toolkit v0.1.3. The source code for the software used for the simulations presented in this paper is also available from http://sourceforge.net/projects/tst. Installation: Unpack and compile according to the instructions given in the INSTALL file The code is written in C++ using the cross-platform (Windows, Mac, or Unix/Linux) library Qt (available from www.trolltech.com). (332 KB ZIP) [file pcbi.1000163.s002.zip › TST0.1.3/html/classCellularPotts.html]

Tissue Simulation Toolkit: CellularPotts class Reference

Main Page | Namespace List | Class Hierarchy | Class List | File List | Namespace Members | Class Members | File Members

# CellularPotts Class Reference

`#include <ca.h>`

List of all members.

|  |
| --- |
|  |
| Public Member Functions | |
|  | CellularPotts (std::vector< Cell > \*cells, const int sizex=200, const int sizey=200) |
|  | Constructs a CA field. This should be done in "Dish". |
|  | CellularPotts (void) |
| virtual void | AllocateSigma (int sx, int sy) |
| virtual | ~CellularPotts () |
| int \*\* | SearchNandPlot (Graphics \*g=0, bool get\_neighbours=true) |
|  | Plots the dish to the screen or to a movie and searches the neighbours. |
| void | Plot (Graphics \*g) |
|  | Plot the dish to Graphics window g. |
| int \*\* | SearchNeighbours (void) |
|  | Searches the cells' neighbors without plotting. |
| int | Mass (void) |
|  | Return the total area occupied by the cells. |
| void | PlotSigma (Graphics \*g, int mag=2) |
| void | DivideCells (void) |
|  | Divide all cells. |
| void | DivideCells (std::vector< bool > which\_cells) |
| int | AmoebaeMove (PDE \*PDEfield=0) |
|  | Monte Carlo Step. Returns summed energy change. |
| void | ReadZygotePicture (void) |
|  | Read initial cell shape from XPM file. Reads the initial cell shape from an include xpm picture called "ZYGXPM(ZYGOTE)", and it allocates enough cells for it to the Dish. |
| void | ConstructInitCells (Dish &beast) |
| int | Time () const |
|  | Returns the number of completed Monte Carlo steps. |
| int | ZygoteArea () const |
| int | SizeX () const |
|  | Return the horizontal size of the CA plane. |
| int | SizeY () const |
|  | Return the vertical size of the CA plane. |
| int | Sigma (const int x, const int y) const |
|  | Return the value of lattice site (x,y). |
| void | Replace (Graphics \*g) |
| Dir \* | FindCellDirections (void) const |
| int | ThrowInCells (int n, int cellsize) |
|  | Initialize the CA plane with n circular cells fitting in a cellsize^2 square. |
| int | GrowInCells (int n\_cells, int cellsize, double subfield=1.) |
|  | Initialize the CA plane with n cells using an Eden growth algorithm. |
| int | GrowInCells (int n\_cells, int cell\_size, int sx, int sy, int offset\_x, int offset\_y) |
| Cell & | AddCell (Dish &beast) |
|  | Adds a new Cell and returns a reference to it. |
| void | ShowDirections (Graphics &g, const Dir \*celldir) const |
|  | Display the division planes returned by FindCellDirections. |
| double | MeanCellArea (void) const |
|  | Returns the mean area of the cells. |
| double | CellDensity (void) const |
|  | Returns the cell density. |
| void | ResetTargetLengths (void) |
|  | Set target lengths of all cells to the value given in parameter file. |
| void | SetRandomTypes (void) |
|  | Give each cell a random cell type. |
| void | GrowAndDivideCells (int growth\_rate) |
| Cell & | getCell (int c) |
| double | DrawConvexHull (Graphics \*g, int color=1) |
| double | Compactness (double \*res\_compactness=0, double \*res\_area=0, double \*res\_cell\_area=0) |
| Public Attributes | |
| int | spins\_converted |
| Protected Member Functions | |
| void | BaseInitialisation (std::vector< Cell > \*cell) |
| Protected Attributes | |
| int \*\* | sigma |
| int | sizex |
| int | sizey |
| Friends | |
| class | Info |
| class | Morphometry |

---

## Constructor & Destructor Documentation

|  |  |  |  |  |  |  |  |  |  |  |  |  |  |  |  |  |
| --- | --- | --- | --- | --- | --- | --- | --- | --- | --- | --- | --- | --- | --- | --- | --- | --- |
| |  |  |  |  | | --- | --- | --- | --- | | CellularPotts::CellularPotts | ( | std::vector< Cell > \* | *cells*, | |  |  | const int | *sizex* = 200, | |  |  | const int | *sizey* = 200 | |  | ) |  | | |

|  |  |
| --- | --- |
|  | Constructs a CA field. This should be done in "Dish". |

|  |  |  |  |  |  |  |
| --- | --- | --- | --- | --- | --- | --- |
| |  |  |  |  |  |  | | --- | --- | --- | --- | --- | --- | | CellularPotts::CellularPotts | ( | void |  | ) |  | |

|  |  |
| --- | --- |
|  |  |

|  |  |  |  |  |  |
| --- | --- | --- | --- | --- | --- |
| |  |  |  |  |  | | --- | --- | --- | --- | --- | | CellularPotts::~CellularPotts | ( |  | ) | `[virtual]` | |

|  |  |
| --- | --- |
|  |  |

---

## Member Function Documentation

|  |  |  |  |  |  |  |
| --- | --- | --- | --- | --- | --- | --- |
| |  |  |  |  |  |  | | --- | --- | --- | --- | --- | --- | | Cell& CellularPotts::AddCell | ( | Dish & | *beast* | ) | `[inline]` | |

|  |  |
| --- | --- |
|  | Adds a new Cell and returns a reference to it. |

|  |  |  |  |  |  |  |  |  |  |  |  |  |
| --- | --- | --- | --- | --- | --- | --- | --- | --- | --- | --- | --- | --- |
| |  |  |  |  | | --- | --- | --- | --- | | void CellularPotts::AllocateSigma | ( | int | *sx*, | |  |  | int | *sy* | |  | ) | `[virtual]` | | |

|  |  |
| --- | --- |
|  |  |

|  |  |  |  |  |  |  |
| --- | --- | --- | --- | --- | --- | --- |
| |  |  |  |  |  |  | | --- | --- | --- | --- | --- | --- | | int CellularPotts::AmoebaeMove | ( | PDE \* | *PDEfield* = 0 | ) |  | |

|  |  |
| --- | --- |
|  | Monte Carlo Step. Returns summed energy change. Implements the core CPM algorithm. Carries out one MCS. **Returns:**  Total energy change during MCS. |

|  |  |  |  |  |  |  |
| --- | --- | --- | --- | --- | --- | --- |
| |  |  |  |  |  |  | | --- | --- | --- | --- | --- | --- | | void CellularPotts::BaseInitialisation | ( | std::vector< Cell > \* | *cell* | ) | `[protected]` | |

|  |  |
| --- | --- |
|  |  |

|  |  |  |  |  |  |  |
| --- | --- | --- | --- | --- | --- | --- |
| |  |  |  |  |  |  | | --- | --- | --- | --- | --- | --- | | double CellularPotts::CellDensity | ( | void |  | ) | const | |

|  |  |
| --- | --- |
|  | Returns the cell density. Cell density is defined as the area occupied by cells divided by the size of the field. |

|  |  |  |  |  |  |  |  |  |  |  |  |  |  |  |  |  |
| --- | --- | --- | --- | --- | --- | --- | --- | --- | --- | --- | --- | --- | --- | --- | --- | --- |
| |  |  |  |  | | --- | --- | --- | --- | | double CellularPotts::Compactness | ( | double \* | *res\_compactness* = 0, | |  |  | double \* | *res\_area* = 0, | |  |  | double \* | *res\_cell\_area* = 0 | |  | ) |  | | |

|  |  |
| --- | --- |
|  | Calculate compactness (summed\_area/hull\_area) of all cells. This is a good measure for the density. **Returns:**  Compactness. |

|  |  |  |  |  |  |  |
| --- | --- | --- | --- | --- | --- | --- |
| |  |  |  |  |  |  | | --- | --- | --- | --- | --- | --- | | void CellularPotts::ConstructInitCells | ( | Dish & | *beast* | ) |  | |

|  |  |
| --- | --- |
|  |  |

|  |  |  |  |  |  |  |
| --- | --- | --- | --- | --- | --- | --- |
| |  |  |  |  |  |  | | --- | --- | --- | --- | --- | --- | | void CellularPotts::DivideCells | ( | std::vector< bool > | *which\_cells* | ) |  | |

|  |  |  |  |
| --- | --- | --- | --- |
|  | Divide all cells marked "true" in which\_cells. **Parameters:**  |  |  | | --- | --- | | *which\_cells* | is a vector<bool> with the same number of elements as the number of cells. It is a mask indicating which cells should be divided; each cell marked true will be divided. | If which\_cells is empty, this method divides all cells. |

|  |  |  |  |  |  |  |
| --- | --- | --- | --- | --- | --- | --- |
| |  |  |  |  |  |  | | --- | --- | --- | --- | --- | --- | | void CellularPotts::DivideCells | ( | void |  | ) | `[inline]` | |

|  |  |
| --- | --- |
|  | Divide all cells. |

|  |  |  |  |  |  |  |  |  |  |  |  |  |
| --- | --- | --- | --- | --- | --- | --- | --- | --- | --- | --- | --- | --- |
| |  |  |  |  | | --- | --- | --- | --- | | double CellularPotts::DrawConvexHull | ( | Graphics \* | *g*, | |  |  | int | *color* = 1 | |  | ) |  | | |

|  |  |
| --- | --- |
|  | Draw convex hull around all cells. **Returns:**  The area of the convex hull in lattice sites. |

|  |  |  |  |  |  |  |
| --- | --- | --- | --- | --- | --- | --- |
| |  |  |  |  |  |  | | --- | --- | --- | --- | --- | --- | | Dir \* CellularPotts::FindCellDirections | ( | void |  | ) | const | |

|  |  |
| --- | --- |
|  | In this method the principal axes of the cells are computed using the method described in "Biometry", box 15.5 **Returns:**  a pointer to a "new[]"ed array containing the directions. The memory has to be freed afterwards using the delete[] operator |

|  |  |  |  |  |  |  |
| --- | --- | --- | --- | --- | --- | --- |
| |  |  |  |  |  |  | | --- | --- | --- | --- | --- | --- | | Cell& CellularPotts::getCell | ( | int | *c* | ) | `[inline]` | |

|  |  |
| --- | --- |
|  |  |

|  |  |  |  |  |  |  |
| --- | --- | --- | --- | --- | --- | --- |
| |  |  |  |  |  |  | | --- | --- | --- | --- | --- | --- | | void CellularPotts::GrowAndDivideCells | ( | int | *growth\_rate* | ) |  | |

|  |  |
| --- | --- |
|  | Cells grow until twice their original target\_length, then divide, with rate "growth\_rate" |

|  |  |  |  |  |  |  |  |  |  |  |  |  |  |  |  |  |  |  |  |  |  |  |  |  |  |  |  |  |
| --- | --- | --- | --- | --- | --- | --- | --- | --- | --- | --- | --- | --- | --- | --- | --- | --- | --- | --- | --- | --- | --- | --- | --- | --- | --- | --- | --- | --- |
| |  |  |  |  | | --- | --- | --- | --- | | int CellularPotts::GrowInCells | ( | int | *n\_cells*, | |  |  | int | *cell\_size*, | |  |  | int | *sx*, | |  |  | int | *sy*, | |  |  | int | *offset\_x*, | |  |  | int | *offset\_y* | |  | ) |  | | |

|  |  |
| --- | --- |
|  |  |

|  |  |  |  |  |  |  |  |  |  |  |  |  |  |  |  |  |
| --- | --- | --- | --- | --- | --- | --- | --- | --- | --- | --- | --- | --- | --- | --- | --- | --- |
| |  |  |  |  | | --- | --- | --- | --- | | int CellularPotts::GrowInCells | ( | int | *n\_cells*, | |  |  | int | *cellsize*, | |  |  | double | *subfield* = 1. | |  | ) |  | | |

|  |  |  |  |  |  |  |  |
| --- | --- | --- | --- | --- | --- | --- | --- |
|  | Initialize the CA plane with n cells using an Eden growth algorithm. **Parameters:**  |  |  | | --- | --- | | *n:* | Number of cells. | | *cellsize:* | Number of Eden growth iterations. | | *subfield:* | Defines a centered frame of size (size/subfield)^2 in which all cell will be positioned. |  **Returns:**  Index of last cell inserted. |

|  |  |  |  |  |  |  |
| --- | --- | --- | --- | --- | --- | --- |
| |  |  |  |  |  |  | | --- | --- | --- | --- | --- | --- | | int CellularPotts::Mass | ( | void |  | ) | `[inline]` | |

|  |  |
| --- | --- |
|  | Return the total area occupied by the cells. |

|  |  |  |  |  |  |  |
| --- | --- | --- | --- | --- | --- | --- |
| |  |  |  |  |  |  | | --- | --- | --- | --- | --- | --- | | double CellularPotts::MeanCellArea | ( | void |  | ) | const | |

|  |  |
| --- | --- |
|  | Returns the mean area of the cells. |

|  |  |  |  |  |  |  |
| --- | --- | --- | --- | --- | --- | --- |
| |  |  |  |  |  |  | | --- | --- | --- | --- | --- | --- | | void CellularPotts::Plot | ( | Graphics \* | *g* | ) | `[inline]` | |

|  |  |
| --- | --- |
|  | Plot the dish to Graphics window g. |

|  |  |  |  |  |  |  |  |  |  |  |  |  |
| --- | --- | --- | --- | --- | --- | --- | --- | --- | --- | --- | --- | --- |
| |  |  |  |  | | --- | --- | --- | --- | | void CellularPotts::PlotSigma | ( | Graphics \* | *g*, | |  |  | int | *mag* = 2 | |  | ) |  | | |

|  |  |
| --- | --- |
|  | Plot the cells according to their cell identity, not their type. The black lines are omitted. |

|  |  |  |  |  |  |  |
| --- | --- | --- | --- | --- | --- | --- |
| |  |  |  |  |  |  | | --- | --- | --- | --- | --- | --- | | void CellularPotts::ReadZygotePicture | ( | void |  | ) |  | |

|  |  |
| --- | --- |
|  | Read initial cell shape from XPM file. Reads the initial cell shape from an include xpm picture called "ZYGXPM(ZYGOTE)", and it allocates enough cells for it to the Dish. |

|  |  |  |  |  |  |  |
| --- | --- | --- | --- | --- | --- | --- |
| |  |  |  |  |  |  | | --- | --- | --- | --- | --- | --- | | void CellularPotts::Replace | ( | Graphics \* | *g* | ) |  | |

|  |  |
| --- | --- |
|  |  |

|  |  |  |  |  |  |  |
| --- | --- | --- | --- | --- | --- | --- |
| |  |  |  |  |  |  | | --- | --- | --- | --- | --- | --- | | void CellularPotts::ResetTargetLengths | ( | void |  | ) |  | |

|  |  |
| --- | --- |
|  | Set target lengths of all cells to the value given in parameter file. |

|  |  |  |  |  |  |  |  |  |  |  |  |  |
| --- | --- | --- | --- | --- | --- | --- | --- | --- | --- | --- | --- | --- |
| |  |  |  |  | | --- | --- | --- | --- | | int \*\* CellularPotts::SearchNandPlot | ( | Graphics \* | *g* = 0, | |  |  | bool | *get\_neighbours* = true | |  | ) |  | | |

|  |  |
| --- | --- |
|  | Plots the dish to the screen or to a movie and searches the neighbours. These distinct tasks have been lumped together in the same method because both for drawing the black lines between the cells and for searching the neighbours the cell borders have to be determined. |

|  |  |  |  |  |  |  |
| --- | --- | --- | --- | --- | --- | --- |
| |  |  |  |  |  |  | | --- | --- | --- | --- | --- | --- | | int\*\* CellularPotts::SearchNeighbours | ( | void |  | ) | `[inline]` | |

|  |  |
| --- | --- |
|  | Searches the cells' neighbors without plotting. |

|  |  |  |  |  |  |  |
| --- | --- | --- | --- | --- | --- | --- |
| |  |  |  |  |  |  | | --- | --- | --- | --- | --- | --- | | void CellularPotts::SetRandomTypes | ( | void |  | ) |  | |

|  |  |
| --- | --- |
|  | Give each cell a random cell type. The number of cell types is defined by the J parameter file. (See Jtable in parameter file). |

|  |  |  |  |  |  |  |  |  |  |  |  |  |
| --- | --- | --- | --- | --- | --- | --- | --- | --- | --- | --- | --- | --- |
| |  |  |  |  | | --- | --- | --- | --- | | void CellularPotts::ShowDirections | ( | Graphics & | *g*, | |  |  | const Dir \* | *celldir* | |  | ) | const | | |

|  |  |  |  |  |  |
| --- | --- | --- | --- | --- | --- |
|  | Display the division planes returned by FindCellDirections. **Parameters:**  |  |  | | --- | --- | | *g:* | Graphics window | | *celldir:* | cell axes as returned by FindCellDirections. | |

|  |  |  |  |  |  |  |  |  |  |  |  |  |
| --- | --- | --- | --- | --- | --- | --- | --- | --- | --- | --- | --- | --- |
| |  |  |  |  | | --- | --- | --- | --- | | int CellularPotts::Sigma | ( | const int | *x*, | |  |  | const int | *y* | |  | ) | const `[inline]` | | |

|  |  |
| --- | --- |
|  | Return the value of lattice site (x,y). i.e. This will return the index of the cell which occupies site (x,y). |

|  |  |  |  |  |  |
| --- | --- | --- | --- | --- | --- |
| |  |  |  |  |  | | --- | --- | --- | --- | --- | | int CellularPotts::SizeX | ( |  | ) | const `[inline]` | |

|  |  |
| --- | --- |
|  | Return the horizontal size of the CA plane. |

|  |  |  |  |  |  |
| --- | --- | --- | --- | --- | --- |
| |  |  |  |  |  | | --- | --- | --- | --- | --- | | int CellularPotts::SizeY | ( |  | ) | const `[inline]` | |

|  |  |
| --- | --- |
|  | Return the vertical size of the CA plane. |

|  |  |  |  |  |  |  |  |  |  |  |  |  |
| --- | --- | --- | --- | --- | --- | --- | --- | --- | --- | --- | --- | --- |
| |  |  |  |  | | --- | --- | --- | --- | | int CellularPotts::ThrowInCells | ( | int | *n*, | |  |  | int | *cellsize* | |  | ) |  | | |

|  |  |
| --- | --- |
|  | Initialize the CA plane with n circular cells fitting in a cellsize^2 square. ! Fill the plane with initial cells **Returns:**  actual amount of cells (some are not draw due to overlap) |

|  |  |  |  |  |  |
| --- | --- | --- | --- | --- | --- |
| |  |  |  |  |  | | --- | --- | --- | --- | --- | | int CellularPotts::Time | ( |  | ) | const `[inline]` | |

|  |  |
| --- | --- |
|  | Returns the number of completed Monte Carlo steps. |

|  |  |  |  |  |  |
| --- | --- | --- | --- | --- | --- |
| |  |  |  |  |  | | --- | --- | --- | --- | --- | | int CellularPotts::ZygoteArea | ( |  | ) | const `[inline]` | |

|  |  |
| --- | --- |
|  |  |

---

## Friends And Related Function Documentation

|  |  |
| --- | --- |
| |  | | --- | | friend class Info `[friend]` | |

|  |  |
| --- | --- |
|  |  |

|  |  |
| --- | --- |
| |  | | --- | | friend class Morphometry `[friend]` | |

|  |  |
| --- | --- |
|  |  |

---

## Member Data Documentation

|  |  |
| --- | --- |
| |  | | --- | | int\*\* CellularPotts::sigma `[protected]` | |

|  |  |
| --- | --- |
|  |  |

|  |  |
| --- | --- |
| |  | | --- | | int CellularPotts::sizex `[protected]` | |

|  |  |
| --- | --- |
|  |  |

|  |  |
| --- | --- |
| |  | | --- | | int CellularPotts::sizey `[protected]` | |

|  |  |
| --- | --- |
|  |  |

|  |  |
| --- | --- |
| |  | | --- | | int CellularPotts::spins\_converted | |

|  |  |
| --- | --- |
|  |  |

---

The documentation for this class was generated from the following files:

- /home/romer/TST0.1.3/ca.h- /home/romer/TST0.1.3/ca.cpp

---

Generated on Tue Dec 12 16:32:41 2006 for Tissue Simulation Toolkit by

1.3.5
