# Supplementary material for: Contact-Inhibited Chemotaxis in De Novo and Sprouting Blood-Vessel Growth
Source: PLoS Comput Biol. 2008 Sep 19;4(9):e1000163. doi: 10.1371/journal.pcbi.1000163 (PMC2528254; doi:10.1371/journal.pcbi.1000163)
Supplement: Protocol S1 — Tissue Simulation Toolkit v0.1.3. The source code for the software used for the simulations presented in this paper is also available from http://sourceforge.net/projects/tst. Installation: Unpack and compile according to the instructions given in the INSTALL file The code is written in C++ using the cross-platform (Windows, Mac, or Unix/Linux) library Qt (available from www.trolltech.com). (332 KB ZIP) [file pcbi.1000163.s002.zip › TST0.1.3/html/classDir.html]

Tissue Simulation Toolkit: Dir class Reference

Main Page | Namespace List | Class Hierarchy | Class List | File List | Namespace Members | Class Members | File Members

# Dir Class Reference

`#include <ca.h>`

List of all members.

|  |
| --- |
|  |
| Public Member Functions | |
|  | Dir () |
| Public Attributes | |
| double | aa1 |
| double | aa2 |
| double | bb1 |
| double | bb2 |
| double | lb1 |
| double | lb2 |
| Friends | |
| class | CellularPotts |

---

## Constructor & Destructor Documentation

|  |  |  |  |  |  |
| --- | --- | --- | --- | --- | --- |
| |  |  |  |  |  | | --- | --- | --- | --- | --- | | Dir::Dir | ( |  | ) | `[inline]` | |

|  |  |
| --- | --- |
|  |  |

---

## Friends And Related Function Documentation

|  |  |
| --- | --- |
| |  | | --- | | friend class CellularPotts `[friend]` | |

|  |  |
| --- | --- |
|  |  |

---

## Member Data Documentation

|  |  |
| --- | --- |
| |  | | --- | | double Dir::aa1 | |

|  |  |
| --- | --- |
|  |  |

|  |  |
| --- | --- |
| |  | | --- | | double Dir::aa2 | |

|  |  |
| --- | --- |
|  |  |

|  |  |
| --- | --- |
| |  | | --- | | double Dir::bb1 | |

|  |  |
| --- | --- |
|  |  |

|  |  |
| --- | --- |
| |  | | --- | | double Dir::bb2 | |

|  |  |
| --- | --- |
|  |  |

|  |  |
| --- | --- |
| |  | | --- | | double Dir::lb1 | |

|  |  |
| --- | --- |
|  |  |

|  |  |
| --- | --- |
| |  | | --- | | double Dir::lb2 | |

|  |  |
| --- | --- |
|  |  |

---

The documentation for this class was generated from the following file:

- /home/romer/TST0.1.3/ca.h

---

Generated on Tue Dec 12 16:32:41 2006 for Tissue Simulation Toolkit by

1.3.5
