# Supplementary material for: Contact-Inhibited Chemotaxis in De Novo and Sprouting Blood-Vessel Growth
Source: PLoS Comput Biol. 2008 Sep 19;4(9):e1000163. doi: 10.1371/journal.pcbi.1000163 (PMC2528254; doi:10.1371/journal.pcbi.1000163)
Supplement: Protocol S1 — Tissue Simulation Toolkit v0.1.3. The source code for the software used for the simulations presented in this paper is also available from http://sourceforge.net/projects/tst. Installation: Unpack and compile according to the instructions given in the INSTALL file The code is written in C++ using the cross-platform (Windows, Mac, or Unix/Linux) library Qt (available from www.trolltech.com). (332 KB ZIP) [file pcbi.1000163.s002.zip › TST0.1.3/html/classDish-members.html]

Tissue Simulation Toolkit: Member List

Main Page | Namespace List | Class Hierarchy | Class List | File List | Namespace Members | Class Members | File Members

# Dish Member List

This is the complete list of members for Dish, including all inherited members.

|  |  |  |
| --- | --- | --- |
| Area(void) const | Dish |  |
| cell | Dish | `[protected]` |
| CellGrowthAndDivision(void) | Dish |  |
| ClearGrads(void) | Dish |  |
| ConstructorBody(void) | Dish |  |
| CountCells(void) const | Dish |  |
| CPM | Dish |  |
| Dish(void) | Dish |  |
| getCell(int c) | Dish | `[inline]` |
| Info class | Dish | `[friend]` |
| Init(void) | Dish |  |
| MeasureChemConcentrations(void) | Dish |  |
| PDEfield | Dish |  |
| Plot(Graphics \*g) | Dish |  |
| SetCellOwner(Cell &which\_cell) | Dish | `[protected]` |
| SizeX(void) | Dish |  |
| SizeY(void) | Dish |  |
| TargetArea(void) const | Dish |  |
| Time(void) const | Dish |  |
| ZygoteArea(void) const | Dish |  |
| ~Dish() | Dish | `[virtual]` |

---

Generated on Tue Dec 12 16:32:41 2006 for Tissue Simulation Toolkit by

1.3.5 
