# Supplementary material for: Contact-Inhibited Chemotaxis in De Novo and Sprouting Blood-Vessel Growth
Source: PLoS Comput Biol. 2008 Sep 19;4(9):e1000163. doi: 10.1371/journal.pcbi.1000163 (PMC2528254; doi:10.1371/journal.pcbi.1000163)
Supplement: Protocol S1 — Tissue Simulation Toolkit v0.1.3. The source code for the software used for the simulations presented in this paper is also available from http://sourceforge.net/projects/tst. Installation: Unpack and compile according to the instructions given in the INSTALL file The code is written in C++ using the cross-platform (Windows, Mac, or Unix/Linux) library Qt (available from www.trolltech.com). (332 KB ZIP) [file pcbi.1000163.s002.zip › TST0.1.3/html/classDish.html]

Tissue Simulation Toolkit: Dish class Reference

Main Page | Namespace List | Class Hierarchy | Class List | File List | Namespace Members | Class Members | File Members

# Dish Class Reference

The virtual Petri dish.
More...

`#include <dish.h>`

List of all members.

|  |
| --- |
|  |
| Public Member Functions | |
|  | Dish (void) |
| void | Init (void) |
|  | Init defines the initial state of the virtual cell culture. |
| void | ConstructorBody (void) |
| virtual | ~Dish () |
| void | Plot (Graphics \*g) |
|  | Plot the Dish to graphics window g. |
| int | ZygoteArea (void) const |
| int | Time (void) const |
|  | Returns the number of completed Monte Carlo Steps. |
| int | CountCells (void) const |
|  | Returns the number of cells in the dish, excluding apoptosed cells. |
| void | CellGrowthAndDivision (void) |
|  | Stretched induced cell growth and division. |
| int | Area (void) const |
|  | . Returns the summed area of all cells in the dish |
| int | TargetArea (void) const |
|  | Returns the summed of all cells target area in the dish. |
| int | SizeX (void) |
|  | Returns the horizontal size of the dish. |
| int | SizeY (void) |
|  | Returns the horizontal size of the dish. |
| Cell & | getCell (int c) |
|  | Returns a reference to cell number "c". |
| void | ClearGrads (void) |
| void | MeasureChemConcentrations (void) |
| Public Attributes | |
| PDE \* | PDEfield |
| CellularPotts \* | CPM |
| Protected Member Functions | |
| void | SetCellOwner (Cell &which\_cell) |
|  | Assign a the cell to the current Dish. |
| Protected Attributes | |
| std::vector< Cell > | cell |
|  | The cells in the Petri dish; accessible to derived classes. |
| Friends | |
| class | Info |

---

## Detailed Description

The virtual Petri dish.

Hosts the cells with states and the CA-plane.

---

## Constructor & Destructor Documentation

|  |  |  |  |  |  |  |
| --- | --- | --- | --- | --- | --- | --- |
| |  |  |  |  |  |  | | --- | --- | --- | --- | --- | --- | | Dish::Dish | ( | void |  | ) |  | |

|  |  |
| --- | --- |
|  |  |

|  |  |  |  |  |  |
| --- | --- | --- | --- | --- | --- |
| |  |  |  |  |  | | --- | --- | --- | --- | --- | | Dish::~Dish | ( |  | ) | `[virtual]` | |

|  |  |
| --- | --- |
|  |  |

---

## Member Function Documentation

|  |  |  |  |  |  |  |
| --- | --- | --- | --- | --- | --- | --- |
| |  |  |  |  |  |  | | --- | --- | --- | --- | --- | --- | | int Dish::Area | ( | void |  | ) | const | |

|  |  |
| --- | --- |
|  | . Returns the summed area of all cells in the dish |

|  |  |  |  |  |  |  |
| --- | --- | --- | --- | --- | --- | --- |
| |  |  |  |  |  |  | | --- | --- | --- | --- | --- | --- | | void Dish::CellGrowthAndDivision | ( | void |  | ) |  | |

|  |  |
| --- | --- |
|  | Stretched induced cell growth and division. See Hogeweg (2000), Journal of Theoretical Biology. Find stretched cells, and increase their target area. Find enlarged cells, and divide them. |

|  |  |  |  |  |  |  |
| --- | --- | --- | --- | --- | --- | --- |
| |  |  |  |  |  |  | | --- | --- | --- | --- | --- | --- | | void Dish::ClearGrads | ( | void |  | ) |  | |

|  |  |
| --- | --- |
|  |  |

|  |  |  |  |  |  |  |
| --- | --- | --- | --- | --- | --- | --- |
| |  |  |  |  |  |  | | --- | --- | --- | --- | --- | --- | | void Dish::ConstructorBody | ( | void |  | ) |  | |

|  |  |
| --- | --- |
|  |  |

|  |  |  |  |  |  |  |
| --- | --- | --- | --- | --- | --- | --- |
| |  |  |  |  |  |  | | --- | --- | --- | --- | --- | --- | | int Dish::CountCells | ( | void |  | ) | const | |

|  |  |
| --- | --- |
|  | Returns the number of cells in the dish, excluding apoptosed cells. |

|  |  |  |  |  |  |  |
| --- | --- | --- | --- | --- | --- | --- |
| |  |  |  |  |  |  | | --- | --- | --- | --- | --- | --- | | Cell& Dish::getCell | ( | int | *c* | ) | `[inline]` | |

|  |  |
| --- | --- |
|  | Returns a reference to cell number "c". |

|  |  |  |  |  |  |  |
| --- | --- | --- | --- | --- | --- | --- |
| |  |  |  |  |  |  | | --- | --- | --- | --- | --- | --- | | void Dish::Init | ( | void |  | ) |  | |

|  |  |
| --- | --- |
|  | Init defines the initial state of the virtual cell culture. Define Init() in your main file describing the simulation set up, within the block INIT { }. See for examples vessel.cpp and sorting.cpp. |

|  |  |  |  |  |  |  |
| --- | --- | --- | --- | --- | --- | --- |
| |  |  |  |  |  |  | | --- | --- | --- | --- | --- | --- | | void Dish::MeasureChemConcentrations | ( | void |  | ) |  | |

|  |  |
| --- | --- |
|  |  |

|  |  |  |  |  |  |  |
| --- | --- | --- | --- | --- | --- | --- |
| |  |  |  |  |  |  | | --- | --- | --- | --- | --- | --- | | void Dish::Plot | ( | Graphics \* | *g* | ) |  | |

|  |  |
| --- | --- |
|  | Plot the Dish to graphics window g. Simply calls CPM->Plot. |

|  |  |  |  |  |  |  |
| --- | --- | --- | --- | --- | --- | --- |
| |  |  |  |  |  |  | | --- | --- | --- | --- | --- | --- | | void Dish::SetCellOwner | ( | Cell & | *which\_cell* | ) | `[protected]` | |

|  |  |
| --- | --- |
|  | Assign a the cell to the current Dish. |

|  |  |  |  |  |  |  |
| --- | --- | --- | --- | --- | --- | --- |
| |  |  |  |  |  |  | | --- | --- | --- | --- | --- | --- | | int Dish::SizeX | ( | void |  | ) |  | |

|  |  |
| --- | --- |
|  | Returns the horizontal size of the dish. |

|  |  |  |  |  |  |  |
| --- | --- | --- | --- | --- | --- | --- |
| |  |  |  |  |  |  | | --- | --- | --- | --- | --- | --- | | int Dish::SizeY | ( | void |  | ) |  | |

|  |  |
| --- | --- |
|  | Returns the horizontal size of the dish. |

|  |  |  |  |  |  |  |
| --- | --- | --- | --- | --- | --- | --- |
| |  |  |  |  |  |  | | --- | --- | --- | --- | --- | --- | | int Dish::TargetArea | ( | void |  | ) | const | |

|  |  |
| --- | --- |
|  | Returns the summed of all cells target area in the dish. |

|  |  |  |  |  |  |  |
| --- | --- | --- | --- | --- | --- | --- |
| |  |  |  |  |  |  | | --- | --- | --- | --- | --- | --- | | int Dish::Time | ( | void |  | ) | const | |

|  |  |
| --- | --- |
|  | Returns the number of completed Monte Carlo Steps. |

|  |  |  |  |  |  |  |
| --- | --- | --- | --- | --- | --- | --- |
| |  |  |  |  |  |  | | --- | --- | --- | --- | --- | --- | | int Dish::ZygoteArea | ( | void |  | ) | const | |

|  |  |
| --- | --- |
|  |  |

---

## Friends And Related Function Documentation

|  |  |
| --- | --- |
| |  | | --- | | friend class Info `[friend]` | |

|  |  |
| --- | --- |
|  |  |

---

## Member Data Documentation

|  |  |
| --- | --- |
| |  | | --- | | std::vector<Cell> Dish::cell `[protected]` | |

|  |  |
| --- | --- |
|  | The cells in the Petri dish; accessible to derived classes. |

|  |  |
| --- | --- |
| |  | | --- | | CellularPotts\* Dish::CPM | |

|  |  |
| --- | --- |
|  |  |

|  |  |
| --- | --- |
| |  | | --- | | PDE\* Dish::PDEfield | |

|  |  |
| --- | --- |
|  |  |

---

The documentation for this class was generated from the following files:

- /home/romer/TST0.1.3/dish.h- /home/romer/TST0.1.3/dish.cpp

---

Generated on Tue Dec 12 16:32:41 2006 for Tissue Simulation Toolkit by

1.3.5
