# Supplementary material for: Contact-Inhibited Chemotaxis in De Novo and Sprouting Blood-Vessel Growth
Source: PLoS Comput Biol. 2008 Sep 19;4(9):e1000163. doi: 10.1371/journal.pcbi.1000163 (PMC2528254; doi:10.1371/journal.pcbi.1000163)
Supplement: Protocol S1 — Tissue Simulation Toolkit v0.1.3. The source code for the software used for the simulations presented in this paper is also available from http://sourceforge.net/projects/tst. Installation: Unpack and compile according to the instructions given in the INSTALL file The code is written in C++ using the cross-platform (Windows, Mac, or Unix/Linux) library Qt (available from www.trolltech.com). (332 KB ZIP) [file pcbi.1000163.s002.zip › TST0.1.3/html/classGraphics.html]

Tissue Simulation Toolkit: Graphics class Reference

Main Page | Namespace List | Class Hierarchy | Class List | File List | Namespace Members | Class Members | File Members

# Graphics Class Reference

API for Graphics windows.
More...

`#include <graph.h>`

Inheritance diagram for Graphics:

List of all members.

|  |
| --- |
|  |
| Public Member Functions | |
| virtual | ~Graphics (void) |
| virtual void | BeginScene (void) |
|  | BeginScene() must be called before calling drawing functions. |
| virtual void | EndScene (void) |
|  | EndScene() must be called to flush the drawing buffer and display the scene. |
| virtual void | Point (int color, int x, int y)=0 |
|  | Plot a point in the Graphics window. |
| virtual void | Line (int x1, int y1, int x2, int y2, int colour)=0 |
|  | Draws a line (obviously... :-). |
| virtual int | GetXYCoo (int \*X, int \*Y)=0 |
|  | Probes the Window for user interaction, with mouse or keyboard. |
| virtual int | XField (void) const |
|  | Returns the width of the Graphics window, in pixels. |
| virtual int | YField (void) const |
|  | Returns the height of the Graphics window, in pixels. |
| virtual void | Write (char \*fname, int quality=-1)=0 |
|  | Writes the Image to a file. |
| virtual void | TimeStep (void) |
|  | Implement this member function in your simulation code. |
| virtual void | Field (const int \*\*f, int mag=1) |
|  | Plots a field of values given by \*\*f, using color coding given by colormap file. |

---

## Detailed Description

API for Graphics windows.

No implementation here. Implemented by X11Graphics and QtGraphics.

---

## Constructor & Destructor Documentation

|  |  |  |  |  |  |  |
| --- | --- | --- | --- | --- | --- | --- |
| |  |  |  |  |  |  | | --- | --- | --- | --- | --- | --- | | virtual Graphics::~Graphics | ( | void |  | ) | `[inline, virtual]` | |

|  |  |
| --- | --- |
|  |  |

---

## Member Function Documentation

|  |  |  |  |  |  |  |
| --- | --- | --- | --- | --- | --- | --- |
| |  |  |  |  |  |  | | --- | --- | --- | --- | --- | --- | | virtual void Graphics::BeginScene | ( | void |  | ) | `[inline, virtual]` | |

|  |  |
| --- | --- |
|  | BeginScene() must be called before calling drawing functions. Reimplemented in QtGraphics, and X11Graphics. |

|  |  |  |  |  |  |  |
| --- | --- | --- | --- | --- | --- | --- |
| |  |  |  |  |  |  | | --- | --- | --- | --- | --- | --- | | virtual void Graphics::EndScene | ( | void |  | ) | `[inline, virtual]` | |

|  |  |
| --- | --- |
|  | EndScene() must be called to flush the drawing buffer and display the scene. Reimplemented in QtGraphics, and X11Graphics. |

|  |  |  |  |  |  |  |  |  |  |  |  |  |
| --- | --- | --- | --- | --- | --- | --- | --- | --- | --- | --- | --- | --- |
| |  |  |  |  | | --- | --- | --- | --- | | virtual void Graphics::Field | ( | const int \*\* | *f*, | |  |  | int | *mag* = 1 | |  | ) | `[inline, virtual]` | | |

|  |  |  |  |  |  |
| --- | --- | --- | --- | --- | --- |
|  | Plots a field of values given by \*\*f, using color coding given by colormap file. Only implemented in X11Graphics. No checks. Usage not recommended. **Parameters:**  |  |  | | --- | --- | | *f:* | Double pointer to array of integers, giving color indices using standard colormap ('default.ctb'). | | *mag:* | magnification factor. |  Reimplemented in X11Graphics. |

|  |  |  |  |  |  |  |  |  |  |  |  |  |
| --- | --- | --- | --- | --- | --- | --- | --- | --- | --- | --- | --- | --- |
| |  |  |  |  | | --- | --- | --- | --- | | virtual int Graphics::GetXYCoo | ( | int \* | *X*, | |  |  | int \* | *Y* | |  | ) | `[pure virtual]` | | |

|  |  |  |  |
| --- | --- | --- | --- |
|  | Probes the Window for user interaction, with mouse or keyboard. This function should return immediately, and return 0 if there was no user interaction. **Parameters:**  |  |  | | --- | --- | | *\*X,\*Y:* | Pointer where the clicked coordinate will be stored. |  Implemented in QtGraphics, and X11Graphics. |

|  |  |  |  |  |  |  |  |  |  |  |  |  |  |  |  |  |  |  |  |  |  |  |  |  |
| --- | --- | --- | --- | --- | --- | --- | --- | --- | --- | --- | --- | --- | --- | --- | --- | --- | --- | --- | --- | --- | --- | --- | --- | --- |
| |  |  |  |  | | --- | --- | --- | --- | | virtual void Graphics::Line | ( | int | *x1*, | |  |  | int | *y1*, | |  |  | int | *x2*, | |  |  | int | *y2*, | |  |  | int | *colour* | |  | ) | `[pure virtual]` | | |

|  |  |  |  |  |  |  |  |
| --- | --- | --- | --- | --- | --- | --- | --- |
|  | Draws a line (obviously... :-). **Parameters:**  |  |  | | --- | --- | | *x1,y1:* | First coordinate pair. | | *x2,y2:* | Second coordinate pair. | | *color:* | Color of the line, as given in the colormap file "default.ctb". |  Implemented in QtGraphics, and X11Graphics. |

|  |  |  |  |  |  |  |  |  |  |  |  |  |  |  |  |  |
| --- | --- | --- | --- | --- | --- | --- | --- | --- | --- | --- | --- | --- | --- | --- | --- | --- |
| |  |  |  |  | | --- | --- | --- | --- | | virtual void Graphics::Point | ( | int | *color*, | |  |  | int | *x*, | |  |  | int | *y* | |  | ) | `[pure virtual]` | | |

|  |  |  |  |  |  |
| --- | --- | --- | --- | --- | --- |
|  | Plot a point in the Graphics window. **Parameters:**  |  |  | | --- | --- | | *color:* | Color index, as defined in colormap file "default.ctb", which should be in the same directory as the executable. | | *x,y:* | Coordinate of point, in Graphics coordinates (typically twice as large as the cellular automata coordinates). |  Implemented in QtGraphics, and X11Graphics. |

|  |  |  |  |  |  |  |
| --- | --- | --- | --- | --- | --- | --- |
| |  |  |  |  |  |  | | --- | --- | --- | --- | --- | --- | | virtual void Graphics::TimeStep | ( | void |  | ) | `[inline, virtual]` | |

|  |  |
| --- | --- |
|  | Implement this member function in your simulation code. Include all actions that should be carried out during a simulation step, including PDE and CPM simulation steps. See the included examples (vessel.cpp, sorting.cpp) for more information. Reimplemented in QtGraphics, and X11Graphics. |

|  |  |  |  |  |  |  |  |  |  |  |  |  |
| --- | --- | --- | --- | --- | --- | --- | --- | --- | --- | --- | --- | --- |
| |  |  |  |  | | --- | --- | --- | --- | | virtual void Graphics::Write | ( | char \* | *fname*, | |  |  | int | *quality* = -1 | |  | ) | `[pure virtual]` | | |

|  |  |  |  |  |  |
| --- | --- | --- | --- | --- | --- |
|  | Writes the Image to a file. File format is inferred from file extension. Currently only PNG is supported by the X-Windows implementation; the Qt-implentation supports all formats supported by Qt. **Parameters:**  |  |  | | --- | --- | | *fname:* | File name with standard image file extension (e.g. png). | | *quality:* | Quality of JPEG images, defaults to -1 (no value provided). |  Implemented in QtGraphics, and X11Graphics. |

|  |  |  |  |  |  |  |
| --- | --- | --- | --- | --- | --- | --- |
| |  |  |  |  |  |  | | --- | --- | --- | --- | --- | --- | | virtual int Graphics::XField | ( | void |  | ) | const `[inline, virtual]` | |

|  |  |
| --- | --- |
|  | Returns the width of the Graphics window, in pixels. Reimplemented in QtGraphics, and X11Graphics. |

|  |  |  |  |  |  |  |
| --- | --- | --- | --- | --- | --- | --- |
| |  |  |  |  |  |  | | --- | --- | --- | --- | --- | --- | | virtual int Graphics::YField | ( | void |  | ) | const `[inline, virtual]` | |

|  |  |
| --- | --- |
|  | Returns the height of the Graphics window, in pixels. Reimplemented in QtGraphics, and X11Graphics. |

---

The documentation for this class was generated from the following file:

- /home/romer/TST0.1.3/graph.h

---

Generated on Tue Dec 12 16:32:41 2006 for Tissue Simulation Toolkit by

1.3.5
