# Supplementary material for: Contact-Inhibited Chemotaxis in De Novo and Sprouting Blood-Vessel Growth
Source: PLoS Comput Biol. 2008 Sep 19;4(9):e1000163. doi: 10.1371/journal.pcbi.1000163 (PMC2528254; doi:10.1371/journal.pcbi.1000163)
Supplement: Protocol S1 — Tissue Simulation Toolkit v0.1.3. The source code for the software used for the simulations presented in this paper is also available from http://sourceforge.net/projects/tst. Installation: Unpack and compile according to the instructions given in the INSTALL file The code is written in C++ using the cross-platform (Windows, Mac, or Unix/Linux) library Qt (available from www.trolltech.com). (332 KB ZIP) [file pcbi.1000163.s002.zip › TST0.1.3/html/classInfo-members.html]

Tissue Simulation Toolkit: Member List

Main Page | Namespace List | Class Hierarchy | Class List | File List | Namespace Members | Class Members | File Members

# Info Member List

This is the complete list of members for Info, including all inherited members.

|  |  |  |
| --- | --- | --- |
| ClickCell(Graphics \*g) | Info |  |
| Info(Dish &dish, Graphics &graphics, std::ostream &out=std::cout) | Info |  |
| Menu(void) | Info |  |
| WriteCOM(int cell\_id, std::ostream &out=std::cout) | Info |  |

---

Generated on Tue Dec 12 16:32:41 2006 for Tissue Simulation Toolkit by

1.3.5 
