# Supplementary material for: Contact-Inhibited Chemotaxis in De Novo and Sprouting Blood-Vessel Growth
Source: PLoS Comput Biol. 2008 Sep 19;4(9):e1000163. doi: 10.1371/journal.pcbi.1000163 (PMC2528254; doi:10.1371/journal.pcbi.1000163)
Supplement: Protocol S1 — Tissue Simulation Toolkit v0.1.3. The source code for the software used for the simulations presented in this paper is also available from http://sourceforge.net/projects/tst. Installation: Unpack and compile according to the instructions given in the INSTALL file The code is written in C++ using the cross-platform (Windows, Mac, or Unix/Linux) library Qt (available from www.trolltech.com). (332 KB ZIP) [file pcbi.1000163.s002.zip › TST0.1.3/html/classInfo.html]

Tissue Simulation Toolkit: Info class Reference

Main Page | Namespace List | Class Hierarchy | Class List | File List | Namespace Members | Class Members | File Members

# Info Class Reference

Enables interactive querying of the simulation.
More...

`#include <info.h>`

List of all members.

|  |
| --- |
|  |
| Public Member Functions | |
|  | Info (Dish &dish, Graphics &graphics, std::ostream &out=std::cout) |
|  | Constructs and Info class dish with specified graphics window. |
| void | Menu (void) |
|  | Reads out key presses in the Graphics window and interprets them. |
| void | WriteCOM (int cell\_id, std::ostream &out=std::cout) |
|  | Writes center of mass of cell "cell\_id" to stream out. |
| Cell & | ClickCell (Graphics \*g) |
|  | Waits until the user clicks a cell and returns a reference to it. |

---

## Detailed Description

Enables interactive querying of the simulation.

Only using key presses, to be defined in Info::Menu().

Right-click menu in future Qt-linked versions?

---

## Constructor & Destructor Documentation

|  |  |  |  |  |  |  |  |  |  |  |  |  |  |  |  |  |
| --- | --- | --- | --- | --- | --- | --- | --- | --- | --- | --- | --- | --- | --- | --- | --- | --- |
| |  |  |  |  | | --- | --- | --- | --- | | Info::Info | ( | Dish & | *dish*, | |  |  | Graphics & | *graphics*, | |  |  | std::ostream & | *out* = std::cout | |  | ) |  | | |

|  |  |  |  |  |  |  |  |
| --- | --- | --- | --- | --- | --- | --- | --- |
|  | Constructs and Info class dish with specified graphics window. **Parameters:**  |  |  | | --- | --- | | *dish:* | The virtual Petri dish to query. | | *graphics:* | The Graphics window displaying the dish. | | *out:* | (optional) Stream into which info is written. Default: console. | |

---

## Member Function Documentation

|  |  |  |  |  |  |  |
| --- | --- | --- | --- | --- | --- | --- |
| |  |  |  |  |  |  | | --- | --- | --- | --- | --- | --- | | Cell & Info::ClickCell | ( | Graphics \* | *g* | ) |  | |

|  |  |
| --- | --- |
|  | Waits until the user clicks a cell and returns a reference to it. |

|  |  |  |  |  |  |  |
| --- | --- | --- | --- | --- | --- | --- |
| |  |  |  |  |  |  | | --- | --- | --- | --- | --- | --- | | void Info::Menu | ( | void |  | ) |  | |

|  |  |
| --- | --- |
|  | Reads out key presses in the Graphics window and interprets them. If you want to define extra interactive queries, redefine this method. If you want a nice GUI menu, reimplement this method. |

|  |  |  |  |  |  |  |  |  |  |  |  |  |
| --- | --- | --- | --- | --- | --- | --- | --- | --- | --- | --- | --- | --- |
| |  |  |  |  | | --- | --- | --- | --- | | void Info::WriteCOM | ( | int | *cell\_id*, | |  |  | std::ostream & | *out* = std::cout | |  | ) |  | | |

|  |  |
| --- | --- |
|  | Writes center of mass of cell "cell\_id" to stream out. |

---

The documentation for this class was generated from the following files:

- /home/romer/TST0.1.3/info.h- /home/romer/TST0.1.3/info.cpp

---

Generated on Tue Dec 12 16:32:41 2006 for Tissue Simulation Toolkit by

1.3.5
