# Supplementary material for: Contact-Inhibited Chemotaxis in De Novo and Sprouting Blood-Vessel Growth
Source: PLoS Comput Biol. 2008 Sep 19;4(9):e1000163. doi: 10.1371/journal.pcbi.1000163 (PMC2528254; doi:10.1371/journal.pcbi.1000163)
Supplement: Protocol S1 — Tissue Simulation Toolkit v0.1.3. The source code for the software used for the simulations presented in this paper is also available from http://sourceforge.net/projects/tst. Installation: Unpack and compile according to the instructions given in the INSTALL file The code is written in C++ using the cross-platform (Windows, Mac, or Unix/Linux) library Qt (available from www.trolltech.com). (332 KB ZIP) [file pcbi.1000163.s002.zip › TST0.1.3/html/classParameter-members.html]

Tissue Simulation Toolkit: Member List

Main Page | Namespace List | Class Hierarchy | Class List | File List | Namespace Members | Class Members | File Members

# Parameter Member List

This is the complete list of members for Parameter, including all inherited members.

|  |  |  |
| --- | --- | --- |
| border\_energy | Parameter |  |
| chemotaxis | Parameter |  |
| CleanUp(void) | Parameter |  |
| conn\_diss | Parameter |  |
| datadir | Parameter |  |
| decay\_rate | Parameter |  |
| diff\_coeff | Parameter |  |
| divisions | Parameter |  |
| dt | Parameter |  |
| dx | Parameter |  |
| extensiononly | Parameter |  |
| graphics | Parameter |  |
| Jtable | Parameter |  |
| lambda | Parameter |  |
| lambda2 | Parameter |  |
| mcs | Parameter |  |
| n\_chem | Parameter |  |
| n\_init\_cells | Parameter |  |
| neighbours | Parameter |  |
| Parameter() | Parameter |  |
| pde\_its | Parameter |  |
| periodic\_boundaries | Parameter |  |
| Read(const char \*filename) | Parameter |  |
| relaxation | Parameter |  |
| rseed | Parameter |  |
| saturation | Parameter |  |
| secr\_rate | Parameter |  |
| size\_init\_cells | Parameter |  |
| sizex | Parameter |  |
| sizey | Parameter |  |
| storage\_stride | Parameter |  |
| store | Parameter |  |
| subfield | Parameter |  |
| T | Parameter |  |
| target\_area | Parameter |  |
| target\_length | Parameter |  |
| vecadherinknockout | Parameter |  |
| Write(ostream &os) const | Parameter |  |
| ~Parameter() | Parameter |  |

---

Generated on Tue Dec 12 16:32:41 2006 for Tissue Simulation Toolkit by

1.3.5 
