# Supplementary material for: Contact-Inhibited Chemotaxis in De Novo and Sprouting Blood-Vessel Growth
Source: PLoS Comput Biol. 2008 Sep 19;4(9):e1000163. doi: 10.1371/journal.pcbi.1000163 (PMC2528254; doi:10.1371/journal.pcbi.1000163)
Supplement: Protocol S1 — Tissue Simulation Toolkit v0.1.3. The source code for the software used for the simulations presented in this paper is also available from http://sourceforge.net/projects/tst. Installation: Unpack and compile according to the instructions given in the INSTALL file The code is written in C++ using the cross-platform (Windows, Mac, or Unix/Linux) library Qt (available from www.trolltech.com). (332 KB ZIP) [file pcbi.1000163.s002.zip › TST0.1.3/html/classParameter.html]

Tissue Simulation Toolkit: Parameter class Reference

Main Page | Namespace List | Class Hierarchy | Class List | File List | Namespace Members | Class Members | File Members

# Parameter Class Reference

`#include <parameter.h>`

List of all members.

|  |
| --- |
|  |
| Public Member Functions | |
|  | Parameter () |
|  | ~Parameter () |
| void | CleanUp (void) |
| void | Read (const char \*filename) |
| void | Write (ostream &os) const |
| Public Attributes | |
| double | T |
| int | target\_area |
| int | target\_length |
| double | lambda |
| double | lambda2 |
| char \* | Jtable |
| int | conn\_diss |
| bool | vecadherinknockout |
| bool | extensiononly |
| int | chemotaxis |
| int | border\_energy |
| int | neighbours |
| bool | periodic\_boundaries |
| int | n\_chem |
| double \* | diff\_coeff |
| double \* | decay\_rate |
| double \* | secr\_rate |
| double | saturation |
| double | dt |
| double | dx |
| int | pde\_its |
| int | n\_init\_cells |
| int | size\_init\_cells |
| int | sizex |
| int | sizey |
| int | divisions |
| int | mcs |
| int | rseed |
| double | subfield |
| int | relaxation |
| int | storage\_stride |
| bool | graphics |
| bool | store |
| char \* | datadir |

---

## Constructor & Destructor Documentation

|  |  |  |  |  |  |
| --- | --- | --- | --- | --- | --- |
| |  |  |  |  |  | | --- | --- | --- | --- | --- | | Parameter::Parameter | ( |  | ) |  | |

|  |  |
| --- | --- |
|  |  |

|  |  |  |  |  |  |
| --- | --- | --- | --- | --- | --- |
| |  |  |  |  |  | | --- | --- | --- | --- | --- | | Parameter::~Parameter | ( |  | ) |  | |

|  |  |
| --- | --- |
|  |  |

---

## Member Function Documentation

|  |  |  |  |  |  |  |
| --- | --- | --- | --- | --- | --- | --- |
| |  |  |  |  |  |  | | --- | --- | --- | --- | --- | --- | | void Parameter::CleanUp | ( | void |  | ) |  | |

|  |  |
| --- | --- |
|  |  |

|  |  |  |  |  |  |  |
| --- | --- | --- | --- | --- | --- | --- |
| |  |  |  |  |  |  | | --- | --- | --- | --- | --- | --- | | void Parameter::Read | ( | const char \* | *filename* | ) |  | |

|  |  |
| --- | --- |
|  |  |

|  |  |  |  |  |  |  |
| --- | --- | --- | --- | --- | --- | --- |
| |  |  |  |  |  |  | | --- | --- | --- | --- | --- | --- | | void Parameter::Write | ( | ostream & | *os* | ) | const | |

|  |  |
| --- | --- |
|  |  |

---

## Member Data Documentation

|  |  |
| --- | --- |
| |  | | --- | | int Parameter::border\_energy | |

|  |  |
| --- | --- |
|  |  |

|  |  |
| --- | --- |
| |  | | --- | | int Parameter::chemotaxis | |

|  |  |
| --- | --- |
|  |  |

|  |  |
| --- | --- |
| |  | | --- | | int Parameter::conn\_diss | |

|  |  |
| --- | --- |
|  |  |

|  |  |
| --- | --- |
| |  | | --- | | char\* Parameter::datadir | |

|  |  |
| --- | --- |
|  |  |

|  |  |
| --- | --- |
| |  | | --- | | double\* Parameter::decay\_rate | |

|  |  |
| --- | --- |
|  |  |

|  |  |
| --- | --- |
| |  | | --- | | double\* Parameter::diff\_coeff | |

|  |  |
| --- | --- |
|  |  |

|  |  |
| --- | --- |
| |  | | --- | | int Parameter::divisions | |

|  |  |
| --- | --- |
|  |  |

|  |  |
| --- | --- |
| |  | | --- | | double Parameter::dt | |

|  |  |
| --- | --- |
|  |  |

|  |  |
| --- | --- |
| |  | | --- | | double Parameter::dx | |

|  |  |
| --- | --- |
|  |  |

|  |  |
| --- | --- |
| |  | | --- | | bool Parameter::extensiononly | |

|  |  |
| --- | --- |
|  |  |

|  |  |
| --- | --- |
| |  | | --- | | bool Parameter::graphics | |

|  |  |
| --- | --- |
|  |  |

|  |  |
| --- | --- |
| |  | | --- | | char\* Parameter::Jtable | |

|  |  |
| --- | --- |
|  |  |

|  |  |
| --- | --- |
| |  | | --- | | double Parameter::lambda | |

|  |  |
| --- | --- |
|  |  |

|  |  |
| --- | --- |
| |  | | --- | | double Parameter::lambda2 | |

|  |  |
| --- | --- |
|  |  |

|  |  |
| --- | --- |
| |  | | --- | | int Parameter::mcs | |

|  |  |
| --- | --- |
|  |  |

|  |  |
| --- | --- |
| |  | | --- | | int Parameter::n\_chem | |

|  |  |
| --- | --- |
|  |  |

|  |  |
| --- | --- |
| |  | | --- | | int Parameter::n\_init\_cells | |

|  |  |
| --- | --- |
|  |  |

|  |  |
| --- | --- |
| |  | | --- | | int Parameter::neighbours | |

|  |  |
| --- | --- |
|  |  |

|  |  |
| --- | --- |
| |  | | --- | | int Parameter::pde\_its | |

|  |  |
| --- | --- |
|  |  |

|  |  |
| --- | --- |
| |  | | --- | | bool Parameter::periodic\_boundaries | |

|  |  |
| --- | --- |
|  |  |

|  |  |
| --- | --- |
| |  | | --- | | int Parameter::relaxation | |

|  |  |
| --- | --- |
|  |  |

|  |  |
| --- | --- |
| |  | | --- | | int Parameter::rseed | |

|  |  |
| --- | --- |
|  |  |

|  |  |
| --- | --- |
| |  | | --- | | double Parameter::saturation | |

|  |  |
| --- | --- |
|  |  |

|  |  |
| --- | --- |
| |  | | --- | | double\* Parameter::secr\_rate | |

|  |  |
| --- | --- |
|  |  |

|  |  |
| --- | --- |
| |  | | --- | | int Parameter::size\_init\_cells | |

|  |  |
| --- | --- |
|  |  |

|  |  |
| --- | --- |
| |  | | --- | | int Parameter::sizex | |

|  |  |
| --- | --- |
|  |  |

|  |  |
| --- | --- |
| |  | | --- | | int Parameter::sizey | |

|  |  |
| --- | --- |
|  |  |

|  |  |
| --- | --- |
| |  | | --- | | int Parameter::storage\_stride | |

|  |  |
| --- | --- |
|  |  |

|  |  |
| --- | --- |
| |  | | --- | | bool Parameter::store | |

|  |  |
| --- | --- |
|  |  |

|  |  |
| --- | --- |
| |  | | --- | | double Parameter::subfield | |

|  |  |
| --- | --- |
|  |  |

|  |  |
| --- | --- |
| |  | | --- | | double Parameter::T | |

|  |  |
| --- | --- |
|  |  |

|  |  |
| --- | --- |
| |  | | --- | | int Parameter::target\_area | |

|  |  |
| --- | --- |
|  |  |

|  |  |
| --- | --- |
| |  | | --- | | int Parameter::target\_length | |

|  |  |
| --- | --- |
|  |  |

|  |  |
| --- | --- |
| |  | | --- | | bool Parameter::vecadherinknockout | |

|  |  |
| --- | --- |
|  |  |

---

The documentation for this class was generated from the following files:

- /home/romer/TST0.1.3/parameter.h- /home/romer/TST0.1.3/parameter.cpp

---

Generated on Tue Dec 12 16:32:41 2006 for Tissue Simulation Toolkit by

1.3.5
