# Supplementary material for: Contact-Inhibited Chemotaxis in De Novo and Sprouting Blood-Vessel Growth
Source: PLoS Comput Biol. 2008 Sep 19;4(9):e1000163. doi: 10.1371/journal.pcbi.1000163 (PMC2528254; doi:10.1371/journal.pcbi.1000163)
Supplement: Protocol S1 — Tissue Simulation Toolkit v0.1.3. The source code for the software used for the simulations presented in this paper is also available from http://sourceforge.net/projects/tst. Installation: Unpack and compile according to the instructions given in the INSTALL file The code is written in C++ using the cross-platform (Windows, Mac, or Unix/Linux) library Qt (available from www.trolltech.com). (332 KB ZIP) [file pcbi.1000163.s002.zip › TST0.1.3/html/classPDE-members.html]

Tissue Simulation Toolkit: Member List

Main Page | Namespace List | Class Hierarchy | Class List | File List | Namespace Members | Class Members | File Members

# PDE Member List

This is the complete list of members for PDE, including all inherited members.

|  |  |  |
| --- | --- | --- |
| AbsorbingBoundaries(void) | PDE |  |
| addtoValue(const int layer, const int x, const int y, const double value) | PDE | `[inline]` |
| AllocateSigma(const int layers, const int sx, const int sy) | PDE | `[protected, virtual]` |
| alt\_sigma | PDE | `[protected]` |
| ContourPlot(Graphics \*g, int layer=0, int colour=1) | PDE |  |
| Diffuse(int repeat) | PDE |  |
| GetChemAmount(const int layer=-1) | PDE |  |
| GradC(int layer=0, int first\_grad\_layer=1) | PDE |  |
| Info class | PDE | `[friend]` |
| Layers() const | PDE | `[inline]` |
| layers | PDE | `[protected]` |
| MapColour(double val) | PDE | `[protected, virtual]` |
| Max(int l) | PDE | `[inline]` |
| Min(int l) | PDE | `[inline]` |
| NoFluxBoundaries(void) | PDE |  |
| PDE(const int layers, const int sizex, const int sizey) | PDE |  |
| PDE(void) | PDE | `[protected]` |
| PeriodicBoundaries(void) | PDE |  |
| Plot(Graphics \*g, const int layer=0) | PDE |  |
| Plot(Graphics \*g, CellularPotts \*cpm, const int layer=0) | PDE |  |
| PlotVectorField(Graphics &g, int stride, int linelength, int first\_grad\_layer=1) | PDE |  |
| Secrete(CellularPotts \*cpm) | PDE |  |
| setValue(const int layer, const int x, const int y, const double value) | PDE | `[inline]` |
| sigma | PDE | `[protected]` |
| Sigma(const int layer, const int x, const int y) const | PDE | `[inline]` |
| SizeX() const | PDE | `[inline]` |
| sizex | PDE | `[protected]` |
| sizey | PDE | `[protected]` |
| SizeY() const | PDE | `[inline]` |
| TheTime(void) const | PDE | `[inline]` |
| ~PDE() | PDE | `[virtual]` |

---

Generated on Tue Dec 12 16:32:41 2006 for Tissue Simulation Toolkit by

1.3.5 
