# Supplementary material for: Contact-Inhibited Chemotaxis in De Novo and Sprouting Blood-Vessel Growth
Source: PLoS Comput Biol. 2008 Sep 19;4(9):e1000163. doi: 10.1371/journal.pcbi.1000163 (PMC2528254; doi:10.1371/journal.pcbi.1000163)
Supplement: Protocol S1 — Tissue Simulation Toolkit v0.1.3. The source code for the software used for the simulations presented in this paper is also available from http://sourceforge.net/projects/tst. Installation: Unpack and compile according to the instructions given in the INSTALL file The code is written in C++ using the cross-platform (Windows, Mac, or Unix/Linux) library Qt (available from www.trolltech.com). (332 KB ZIP) [file pcbi.1000163.s002.zip › TST0.1.3/html/classPDE.html]

Tissue Simulation Toolkit: PDE class Reference

Main Page | Namespace List | Class Hierarchy | Class List | File List | Namespace Members | Class Members | File Members

# PDE Class Reference

`#include <pde.h>`

List of all members.

|  |
| --- |
|  |
| Public Member Functions | |
|  | PDE (const int layers, const int sizex, const int sizey) |
|  | Constructor for PDE object containing arbitrary number of planes. |
| virtual | ~PDE () |
| void | Plot (Graphics \*g, const int layer=0) |
|  | Plots one layer of the PDE plane to a Graphics window. |
| void | Plot (Graphics \*g, CellularPotts \*cpm, const int layer=0) |
|  | Plots one layer of the PDE to a Graphics window, but not over the cells. |
| void | ContourPlot (Graphics \*g, int layer=0, int colour=1) |
|  | Plots the PDE field using contour lines. |
| int | SizeX () const |
|  | Returns the horizontal size of the PDE planes. |
| int | SizeY () const |
|  | Returns the vertical size of the PDE planes. |
| int | Layers () const |
|  | Returns the number of PDE layers in the PDE object. |
| double | Sigma (const int layer, const int x, const int y) const |
|  | Returns the value of grid point x,y of PDE plane "layer". |
| void | setValue (const int layer, const int x, const int y, const double value) |
|  | Sets grid point x,y of PDE plane "layer" to value "value". |
| void | addtoValue (const int layer, const int x, const int y, const double value) |
|  | Adds a number to a PDE grid point. |
| double | Max (int l) |
|  | Gets the maximum value of PDE layer l. |
| double | Min (int l) |
|  | Returns the minimum value of PDE layer l. |
| void | Diffuse (int repeat) |
|  | Carry out $n$ diffusion steps for all PDE planes. |
| void | NoFluxBoundaries (void) |
|  | Implementation of no-flux boundaries. |
| void | AbsorbingBoundaries (void) |
|  | Implementation of absorbing boundaries. |
| void | PeriodicBoundaries (void) |
|  | Implementation of periodic boundaries. |
| void | Secrete (CellularPotts \*cpm) |
|  | Reaction and interaction of CPM plane with PDE planes. |
| double | TheTime (void) const |
|  | Returns cumulative "simulated" time, i.e. number of time steps \* dt. |
| double | GetChemAmount (const int layer=-1) |
|  | Returns summed amount of chemical in PDE plane "layer". |
| void | GradC (int layer=0, int first\_grad\_layer=1) |
| void | PlotVectorField (Graphics &g, int stride, int linelength, int first\_grad\_layer=1) |
| Protected Member Functions | |
| virtual int | MapColour (double val) |
|  | Used in Plot. Takes a color and turns it into a grey value. |
|  | PDE (void) |
|  | empty constructor (necessary for derivation) |
| virtual double \*\*\* | AllocateSigma (const int layers, const int sx, const int sy) |
|  | Allocates a PDE plane (internal use). |
| Protected Attributes | |
| double \*\*\* | sigma |
| double \*\*\* | alt\_sigma |
| int | sizex |
| int | sizey |
| int | layers |
| Friends | |
| class | Info |

---

## Constructor & Destructor Documentation

|  |  |  |  |  |  |  |  |  |  |  |  |  |  |  |  |  |
| --- | --- | --- | --- | --- | --- | --- | --- | --- | --- | --- | --- | --- | --- | --- | --- | --- |
| |  |  |  |  | | --- | --- | --- | --- | | PDE::PDE | ( | const int | *layers*, | |  |  | const int | *sizex*, | |  |  | const int | *sizey* | |  | ) |  | | |

|  |  |  |  |  |  |  |  |
| --- | --- | --- | --- | --- | --- | --- | --- |
|  | Constructor for PDE object containing arbitrary number of planes. **Parameters:**  |  |  | | --- | --- | | *layers:* | Number of PDE planes | | *sizex:* | horizontal size of PDE planes | | *sizey:* | vertical size of PDE planes | |

|  |  |  |  |  |  |
| --- | --- | --- | --- | --- | --- |
| |  |  |  |  |  | | --- | --- | --- | --- | --- | | PDE::~PDE | ( |  | ) | `[virtual]` | |

|  |  |
| --- | --- |
|  |  |

|  |  |  |  |  |  |  |
| --- | --- | --- | --- | --- | --- | --- |
| |  |  |  |  |  |  | | --- | --- | --- | --- | --- | --- | | PDE::PDE | ( | void |  | ) | `[protected]` | |

|  |  |
| --- | --- |
|  | empty constructor (necessary for derivation) |

---

## Member Function Documentation

|  |  |  |  |  |  |  |
| --- | --- | --- | --- | --- | --- | --- |
| |  |  |  |  |  |  | | --- | --- | --- | --- | --- | --- | | void PDE::AbsorbingBoundaries | ( | void |  | ) |  | |

|  |  |
| --- | --- |
|  | Implementation of absorbing boundaries. Called internally (optionally) by Diffuse(). |

|  |  |  |  |  |  |  |  |  |  |  |  |  |  |  |  |  |  |  |  |  |
| --- | --- | --- | --- | --- | --- | --- | --- | --- | --- | --- | --- | --- | --- | --- | --- | --- | --- | --- | --- | --- |
| |  |  |  |  | | --- | --- | --- | --- | | void PDE::addtoValue | ( | const int | *layer*, | |  |  | const int | *x*, | |  |  | const int | *y*, | |  |  | const double | *value* | |  | ) | `[inline]` | | |

|  |  |  |  |  |  |  |  |
| --- | --- | --- | --- | --- | --- | --- | --- |
|  | Adds a number to a PDE grid point. **Parameters:**  |  |  | | --- | --- | | *layer:* | PDE plane. | | *x,y:* | grid point | | *value:* | value to add | |

|  |  |  |  |  |  |  |  |  |  |  |  |  |  |  |  |  |
| --- | --- | --- | --- | --- | --- | --- | --- | --- | --- | --- | --- | --- | --- | --- | --- | --- |
| |  |  |  |  | | --- | --- | --- | --- | | double \*\*\* PDE::AllocateSigma | ( | const int | *layers*, | |  |  | const int | *sx*, | |  |  | const int | *sy* | |  | ) | `[protected, virtual]` | | |

|  |  |
| --- | --- |
|  | Allocates a PDE plane (internal use). For internal use, can be reimplemented in derived class to change method of memory allocation. |

|  |  |  |  |  |  |  |  |  |  |  |  |  |  |  |  |  |
| --- | --- | --- | --- | --- | --- | --- | --- | --- | --- | --- | --- | --- | --- | --- | --- | --- |
| |  |  |  |  | | --- | --- | --- | --- | | void PDE::ContourPlot | ( | Graphics \* | *g*, | |  |  | int | *layer* = 0, | |  |  | int | *colour* = 1 | |  | ) |  | | |

|  |  |  |  |  |  |  |  |
| --- | --- | --- | --- | --- | --- | --- | --- |
|  | Plots the PDE field using contour lines. **Parameters:**  |  |  | | --- | --- | | *g:* | Graphics window. | | *layer:* | The PDE plane to be plotted. Default layer 0. | | *colour:* | Color to use for the contour lines, as defined in the "default.ctb" color map file, which should be in the same directory as the executable. Default color 1 (black in the default color map). | |

|  |  |  |  |  |  |  |
| --- | --- | --- | --- | --- | --- | --- |
| |  |  |  |  |  |  | | --- | --- | --- | --- | --- | --- | | void PDE::Diffuse | ( | int | *repeat* | ) |  | |

|  |  |  |  |
| --- | --- | --- | --- |
|  | Carry out $n$ diffusion steps for all PDE planes. We use a forward Euler method here. Can be replaced for better algorithm. **Parameters:**  |  |  | | --- | --- | | *repeat:* | Number of steps. | Time step dt, space step dx, diffusion coefficient diff\_coeff and boundary conditions (bool periodic\_boundary) are set as global parameters in a parameter file using class Parameter. |

|  |  |  |  |  |  |  |
| --- | --- | --- | --- | --- | --- | --- |
| |  |  |  |  |  |  | | --- | --- | --- | --- | --- | --- | | double PDE::GetChemAmount | ( | const int | *layer* = -1 | ) |  | |

|  |  |  |  |
| --- | --- | --- | --- |
|  | Returns summed amount of chemical in PDE plane "layer". **Parameters:**  |  |  | | --- | --- | | *layer:* | The PDE plane of which to sum the chemicals. layer=-1 (default) returns the summed amount of chemical in all planes. | |

|  |  |  |  |  |  |  |  |  |  |  |  |  |
| --- | --- | --- | --- | --- | --- | --- | --- | --- | --- | --- | --- | --- |
| |  |  |  |  | | --- | --- | --- | --- | | void PDE::GradC | ( | int | *layer* = 0, | |  |  | int | *first\_grad\_layer* = 1 | |  | ) |  | | |

|  |  |  |  |  |  |
| --- | --- | --- | --- | --- | --- |
|  | Calculates the first and second order gradients, i.e. gradx, grady, gradxx, gradxy and gradyy and puts them in the next three chemical fields. Not currently used and might need some redoing. Make sure you have allocated sufficient fields (this method generates five planes). **Parameters:**  |  |  | | --- | --- | | *layer:* | PDE plane of which to calculate the gradients (default 0) | | *first\_grad\_layer:* | first plane of five in which to write the results (default 1). | |

|  |  |  |  |  |  |
| --- | --- | --- | --- | --- | --- |
| |  |  |  |  |  | | --- | --- | --- | --- | --- | | int PDE::Layers | ( |  | ) | const `[inline]` | |

|  |  |
| --- | --- |
|  | Returns the number of PDE layers in the PDE object. |

|  |  |  |  |  |  |  |
| --- | --- | --- | --- | --- | --- | --- |
| |  |  |  |  |  |  | | --- | --- | --- | --- | --- | --- | | virtual int PDE::MapColour | ( | double | *val* | ) | `[protected, virtual]` | |

|  |  |  |  |
| --- | --- | --- | --- |
|  | Used in Plot. Takes a color and turns it into a grey value. **Parameters:**  |  |  | | --- | --- | | *val:* | Value from PDE plane. | Implement this function in you main simulation code. See e.g. vessel.cpp. |

|  |  |  |  |  |  |  |
| --- | --- | --- | --- | --- | --- | --- |
| |  |  |  |  |  |  | | --- | --- | --- | --- | --- | --- | | double PDE::Max | ( | int | *l* | ) | `[inline]` | |

|  |  |  |  |
| --- | --- | --- | --- |
|  | Gets the maximum value of PDE layer l. **Parameters:**  |  |  | | --- | --- | | *l:* | layer |  **Returns:**  Maximum value in layer l. |

|  |  |  |  |  |  |  |
| --- | --- | --- | --- | --- | --- | --- |
| |  |  |  |  |  |  | | --- | --- | --- | --- | --- | --- | | double PDE::Min | ( | int | *l* | ) | `[inline]` | |

|  |  |  |  |
| --- | --- | --- | --- |
|  | Returns the minimum value of PDE layer l. **Parameters:**  |  |  | | --- | --- | | *l:* | layer |  **Returns:**  Minimum value in layer l. |

|  |  |  |  |  |  |  |
| --- | --- | --- | --- | --- | --- | --- |
| |  |  |  |  |  |  | | --- | --- | --- | --- | --- | --- | | void PDE::NoFluxBoundaries | ( | void |  | ) |  | |

|  |  |
| --- | --- |
|  | Implementation of no-flux boundaries. Called internally (optionally) by Diffuse(). |

|  |  |  |  |  |  |  |
| --- | --- | --- | --- | --- | --- | --- |
| |  |  |  |  |  |  | | --- | --- | --- | --- | --- | --- | | void PDE::PeriodicBoundaries | ( | void |  | ) |  | |

|  |  |
| --- | --- |
|  | Implementation of periodic boundaries. Called internally (optionally) by Diffuse(). |

|  |  |  |  |  |  |  |  |  |  |  |  |  |  |  |  |  |
| --- | --- | --- | --- | --- | --- | --- | --- | --- | --- | --- | --- | --- | --- | --- | --- | --- |
| |  |  |  |  | | --- | --- | --- | --- | | void PDE::Plot | ( | Graphics \* | *g*, | |  |  | CellularPotts \* | *cpm*, | |  |  | const int | *layer* = 0 | |  | ) |  | | |

|  |  |  |  |  |  |  |  |
| --- | --- | --- | --- | --- | --- | --- | --- |
|  | Plots one layer of the PDE to a Graphics window, but not over the cells. **Parameters:**  |  |  | | --- | --- | | *g:* | Graphics window. | | *cpm:* | CellularPotts object containing the cells. | | *layer:* | The PDE plane to be plotted. Default layer 0. | |

|  |  |  |  |  |  |  |  |  |  |  |  |  |
| --- | --- | --- | --- | --- | --- | --- | --- | --- | --- | --- | --- | --- |
| |  |  |  |  | | --- | --- | --- | --- | | void PDE::Plot | ( | Graphics \* | *g*, | |  |  | const int | *layer* = 0 | |  | ) |  | | |

|  |  |  |  |  |  |
| --- | --- | --- | --- | --- | --- |
|  | Plots one layer of the PDE plane to a Graphics window. **Parameters:**  |  |  | | --- | --- | | *g:* | Graphics window. | | *layer:* | The PDE plane to be plotted. Default layer 0. | |

|  |  |  |  |  |  |  |  |  |  |  |  |  |  |  |  |  |  |  |  |  |
| --- | --- | --- | --- | --- | --- | --- | --- | --- | --- | --- | --- | --- | --- | --- | --- | --- | --- | --- | --- | --- |
| |  |  |  |  | | --- | --- | --- | --- | | void PDE::PlotVectorField | ( | Graphics & | *g*, | |  |  | int | *stride*, | |  |  | int | *linelength*, | |  |  | int | *first\_grad\_layer* = 1 | |  | ) |  | | |

|  |  |  |  |  |  |  |  |  |  |
| --- | --- | --- | --- | --- | --- | --- | --- | --- | --- |
|  | Plots a field of the first order gradients, i.e. gradx and grady; assumes you have called GradC before. Not currently used and might need some redoing. **Parameters:**  |  |  | | --- | --- | | *g:* | Graphics window | | *stride:* | Number of grid points between vectors (drawn as lines, currently. | | *linelength:* | Length of vector lines, in pixels. | | *first\_grad\_layer:* | first plane of two which contain the calculated gradients (default 1). | |

|  |  |  |  |  |  |  |
| --- | --- | --- | --- | --- | --- | --- |
| |  |  |  |  |  |  | | --- | --- | --- | --- | --- | --- | | void PDE::Secrete | ( | CellularPotts \* | *cpm* | ) |  | |

|  |  |  |  |
| --- | --- | --- | --- |
|  | Reaction and interaction of CPM plane with PDE planes. **Parameters:**  |  |  | | --- | --- | | *cpm:* | CellularPotts plane the PDE plane interacts with | You should implement this member function as part of your main simulation code. See for an example vessel.cpp. |

|  |  |  |  |  |  |  |  |  |  |  |  |  |  |  |  |  |  |  |  |  |
| --- | --- | --- | --- | --- | --- | --- | --- | --- | --- | --- | --- | --- | --- | --- | --- | --- | --- | --- | --- | --- |
| |  |  |  |  | | --- | --- | --- | --- | | void PDE::setValue | ( | const int | *layer*, | |  |  | const int | *x*, | |  |  | const int | *y*, | |  |  | const double | *value* | |  | ) | `[inline]` | | |

|  |  |  |  |  |  |  |  |
| --- | --- | --- | --- | --- | --- | --- | --- |
|  | Sets grid point x,y of PDE plane "layer" to value "value". **Parameters:**  |  |  | | --- | --- | | *layer:* | PDE plane. | | *x,y:* | grid point | | *value:* | new contents | |

|  |  |  |  |  |  |  |  |  |  |  |  |  |  |  |  |  |
| --- | --- | --- | --- | --- | --- | --- | --- | --- | --- | --- | --- | --- | --- | --- | --- | --- |
| |  |  |  |  | | --- | --- | --- | --- | | double PDE::Sigma | ( | const int | *layer*, | |  |  | const int | *x*, | |  |  | const int | *y* | |  | ) | const `[inline]` | | |

|  |  |  |  |  |  |
| --- | --- | --- | --- | --- | --- |
|  | Returns the value of grid point x,y of PDE plane "layer". Warning, no range checking done. **Parameters:**  |  |  | | --- | --- | | *layer:* | the PDE plane to probe. | | *x,y:* | grid point to probe. | |

|  |  |  |  |  |  |  |
| --- | --- | --- | --- | --- | --- | --- |
| |  |  |  |  |  |  | | --- | --- | --- | --- | --- | --- | | int PDE::SizeX | ( | void |  | ) | const `[inline]` | |

|  |  |
| --- | --- |
|  | Returns the horizontal size of the PDE planes. |

|  |  |  |  |  |  |  |
| --- | --- | --- | --- | --- | --- | --- |
| |  |  |  |  |  |  | | --- | --- | --- | --- | --- | --- | | int PDE::SizeY | ( | void |  | ) | const `[inline]` | |

|  |  |
| --- | --- |
|  | Returns the vertical size of the PDE planes. |

|  |  |  |  |  |  |  |
| --- | --- | --- | --- | --- | --- | --- |
| |  |  |  |  |  |  | | --- | --- | --- | --- | --- | --- | | double PDE::TheTime | ( | void |  | ) | const `[inline]` | |

|  |  |
| --- | --- |
|  | Returns cumulative "simulated" time, i.e. number of time steps \* dt. |

---

## Friends And Related Function Documentation

|  |  |
| --- | --- |
| |  | | --- | | friend class Info `[friend]` | |

|  |  |
| --- | --- |
|  |  |

---

## Member Data Documentation

|  |  |
| --- | --- |
| |  | | --- | | double\*\*\* PDE::alt\_sigma `[protected]` | |

|  |  |
| --- | --- |
|  |  |

|  |  |
| --- | --- |
| |  | | --- | | int PDE::layers `[protected]` | |

|  |  |
| --- | --- |
|  |  |

|  |  |
| --- | --- |
| |  | | --- | | double\*\*\* PDE::sigma `[protected]` | |

|  |  |
| --- | --- |
|  |  |

|  |  |
| --- | --- |
| |  | | --- | | int PDE::sizex `[protected]` | |

|  |  |
| --- | --- |
|  |  |

|  |  |
| --- | --- |
| |  | | --- | | int PDE::sizey `[protected]` | |

|  |  |
| --- | --- |
|  |  |

---

The documentation for this class was generated from the following files:

- /home/romer/TST0.1.3/pde.h- /home/romer/TST0.1.3/pde.cpp

---

Generated on Tue Dec 12 16:32:41 2006 for Tissue Simulation Toolkit by

1.3.5
