# Supplementary material for: Contact-Inhibited Chemotaxis in De Novo and Sprouting Blood-Vessel Growth
Source: PLoS Comput Biol. 2008 Sep 19;4(9):e1000163. doi: 10.1371/journal.pcbi.1000163 (PMC2528254; doi:10.1371/journal.pcbi.1000163)
Supplement: Protocol S1 — Tissue Simulation Toolkit v0.1.3. The source code for the software used for the simulations presented in this paper is also available from http://sourceforge.net/projects/tst. Installation: Unpack and compile according to the instructions given in the INSTALL file The code is written in C++ using the cross-platform (Windows, Mac, or Unix/Linux) library Qt (available from www.trolltech.com). (332 KB ZIP) [file pcbi.1000163.s002.zip › TST0.1.3/html/classQtGraphics-members.html]

Tissue Simulation Toolkit: Member List

Main Page | Namespace List | Class Hierarchy | Class List | File List | Namespace Members | Class Members | File Members

# QtGraphics Member List

This is the complete list of members for QtGraphics, including all inherited members.

|  |  |  |
| --- | --- | --- |
| BeginScene(void) | QtGraphics | `[virtual]` |
| ClearImage(void) | QtGraphics | `[inline]` |
| EndScene(void) | QtGraphics | `[virtual]` |
| Field(const int \*\*f, int mag=1) | Graphics | `[inline, virtual]` |
| GetXYCoo(int \*X, int \*Y) | QtGraphics | `[virtual]` |
| Line(int x1, int y1, int x2, int y2, int colour) | QtGraphics | `[virtual]` |
| Point(int colour, int i, int j) | QtGraphics | `[virtual]` |
| QtGraphics(int xfield, int yfield, const char \*movie\_file=0) | QtGraphics |  |
| QtGraphics(QWidget \*parent, const char \*name, int xfield, int yfield, const char \*movie\_file=0) | QtGraphics | `[inline]` |
| SimulationDone(void) | QtGraphics | `[signal]` |
| TimeStep(void) | QtGraphics | `[virtual]` |
| TimeStepWrap(void) | QtGraphics | `[slot]` |
| Write(char \*fname, int quality=-1) | QtGraphics | `[virtual]` |
| XField(void) const | QtGraphics | `[inline, virtual]` |
| YField(void) const | QtGraphics | `[inline, virtual]` |
| ~Graphics(void) | Graphics | `[inline, virtual]` |
| ~QtGraphics(void) | QtGraphics | `[virtual]` |

---

Generated on Tue Dec 12 16:32:41 2006 for Tissue Simulation Toolkit by

1.3.5 
