# Supplementary material for: Contact-Inhibited Chemotaxis in De Novo and Sprouting Blood-Vessel Growth
Source: PLoS Comput Biol. 2008 Sep 19;4(9):e1000163. doi: 10.1371/journal.pcbi.1000163 (PMC2528254; doi:10.1371/journal.pcbi.1000163)
Supplement: Protocol S1 — Tissue Simulation Toolkit v0.1.3. The source code for the software used for the simulations presented in this paper is also available from http://sourceforge.net/projects/tst. Installation: Unpack and compile according to the instructions given in the INSTALL file The code is written in C++ using the cross-platform (Windows, Mac, or Unix/Linux) library Qt (available from www.trolltech.com). (332 KB ZIP) [file pcbi.1000163.s002.zip › TST0.1.3/html/classQtGraphics.html]

Tissue Simulation Toolkit: QtGraphics class Reference

Main Page | Namespace List | Class Hierarchy | Class List | File List | Namespace Members | Class Members | File Members

# QtGraphics Class Reference

`#include <qtgraph.h>`

Inheritance diagram for QtGraphics:

List of all members.

|  |
| --- |
|  |
| Public Slots | |
| void | TimeStepWrap (void) |
| Signals | |
| void | SimulationDone (void) |
| Public Member Functions | |
|  | QtGraphics (int xfield, int yfield, const char \*movie\_file=0) |
|  | QtGraphics (QWidget \*parent, const char \*name, int xfield, int yfield, const char \*movie\_file=0) |
| virtual | ~QtGraphics (void) |
| virtual void | BeginScene (void) |
|  | BeginScene() must be called before calling drawing functions. |
| virtual void | EndScene (void) |
|  | EndScene() must be called to flush the drawing buffer and display the scene. |
| virtual void | Point (int colour, int i, int j) |
|  | Plot a point in the Graphics window. |
| virtual void | Line (int x1, int y1, int x2, int y2, int colour) |
|  | Draws a line (obviously... :-). |
| virtual int | GetXYCoo (int \*X, int \*Y) |
|  | Probes the Window for user interaction, with mouse or keyboard. |
| virtual int | XField (void) const |
|  | Returns the width of the Graphics window, in pixels. |
| virtual int | YField (void) const |
|  | Returns the height of the Graphics window, in pixels. |
| virtual void | Write (char \*fname, int quality=-1) |
|  | Writes the Image to a file. |
| void | ClearImage (void) |
| virtual void | TimeStep (void) |
|  | Implement this member function in your simulation code. |

---

## Constructor & Destructor Documentation

|  |  |  |  |  |  |  |  |  |  |  |  |  |  |  |  |  |
| --- | --- | --- | --- | --- | --- | --- | --- | --- | --- | --- | --- | --- | --- | --- | --- | --- |
| |  |  |  |  | | --- | --- | --- | --- | | QtGraphics::QtGraphics | ( | int | *xfield*, | |  |  | int | *yfield*, | |  |  | const char \* | *movie\_file* = 0 | |  | ) |  | | |

|  |  |
| --- | --- |
|  |  |

|  |  |  |  |  |  |  |  |  |  |  |  |  |  |  |  |  |  |  |  |  |  |  |  |  |
| --- | --- | --- | --- | --- | --- | --- | --- | --- | --- | --- | --- | --- | --- | --- | --- | --- | --- | --- | --- | --- | --- | --- | --- | --- |
| |  |  |  |  | | --- | --- | --- | --- | | QtGraphics::QtGraphics | ( | QWidget \* | *parent*, | |  |  | const char \* | *name*, | |  |  | int | *xfield*, | |  |  | int | *yfield*, | |  |  | const char \* | *movie\_file* = 0 | |  | ) | `[inline]` | | |

|  |  |
| --- | --- |
|  |  |

|  |  |  |  |  |  |  |
| --- | --- | --- | --- | --- | --- | --- |
| |  |  |  |  |  |  | | --- | --- | --- | --- | --- | --- | | QtGraphics::~QtGraphics | ( | void |  | ) | `[virtual]` | |

|  |  |
| --- | --- |
|  |  |

---

## Member Function Documentation

|  |  |  |  |  |  |  |
| --- | --- | --- | --- | --- | --- | --- |
| |  |  |  |  |  |  | | --- | --- | --- | --- | --- | --- | | void QtGraphics::BeginScene | ( | void |  | ) | `[virtual]` | |

|  |  |
| --- | --- |
|  | BeginScene() must be called before calling drawing functions. Reimplemented from Graphics. |

|  |  |  |  |  |  |  |
| --- | --- | --- | --- | --- | --- | --- |
| |  |  |  |  |  |  | | --- | --- | --- | --- | --- | --- | | void QtGraphics::ClearImage | ( | void |  | ) | `[inline]` | |

|  |  |
| --- | --- |
|  |  |

|  |  |  |  |  |  |  |
| --- | --- | --- | --- | --- | --- | --- |
| |  |  |  |  |  |  | | --- | --- | --- | --- | --- | --- | | void QtGraphics::EndScene | ( | void |  | ) | `[virtual]` | |

|  |  |
| --- | --- |
|  | EndScene() must be called to flush the drawing buffer and display the scene. Reimplemented from Graphics. |

|  |  |  |  |  |  |  |  |  |  |  |  |  |
| --- | --- | --- | --- | --- | --- | --- | --- | --- | --- | --- | --- | --- |
| |  |  |  |  | | --- | --- | --- | --- | | int QtGraphics::GetXYCoo | ( | int \* | *X*, | |  |  | int \* | *Y* | |  | ) | `[virtual]` | | |

|  |  |  |  |
| --- | --- | --- | --- |
|  | Probes the Window for user interaction, with mouse or keyboard. This function should return immediately, and return 0 if there was no user interaction. **Parameters:**  |  |  | | --- | --- | | *\*X,\*Y:* | Pointer where the clicked coordinate will be stored. |  Implements Graphics. |

|  |  |  |  |  |  |  |  |  |  |  |  |  |  |  |  |  |  |  |  |  |  |  |  |  |
| --- | --- | --- | --- | --- | --- | --- | --- | --- | --- | --- | --- | --- | --- | --- | --- | --- | --- | --- | --- | --- | --- | --- | --- | --- |
| |  |  |  |  | | --- | --- | --- | --- | | void QtGraphics::Line | ( | int | *x1*, | |  |  | int | *y1*, | |  |  | int | *x2*, | |  |  | int | *y2*, | |  |  | int | *colour* | |  | ) | `[virtual]` | | |

|  |  |  |  |  |  |  |  |
| --- | --- | --- | --- | --- | --- | --- | --- |
|  | Draws a line (obviously... :-). **Parameters:**  |  |  | | --- | --- | | *x1,y1:* | First coordinate pair. | | *x2,y2:* | Second coordinate pair. | | *color:* | Color of the line, as given in the colormap file "default.ctb". |  Implements Graphics. |

|  |  |  |  |  |  |  |  |  |  |  |  |  |  |  |  |  |
| --- | --- | --- | --- | --- | --- | --- | --- | --- | --- | --- | --- | --- | --- | --- | --- | --- |
| |  |  |  |  | | --- | --- | --- | --- | | void QtGraphics::Point | ( | int | *colour*, | |  |  | int | *i*, | |  |  | int | *j* | |  | ) | `[virtual]` | | |

|  |  |  |  |  |  |
| --- | --- | --- | --- | --- | --- |
|  | Plot a point in the Graphics window. **Parameters:**  |  |  | | --- | --- | | *color:* | Color index, as defined in colormap file "default.ctb", which should be in the same directory as the executable. | | *x,y:* | Coordinate of point, in Graphics coordinates (typically twice as large as the cellular automata coordinates). |  Implements Graphics. |

|  |  |  |  |  |  |  |
| --- | --- | --- | --- | --- | --- | --- |
| |  |  |  |  |  |  | | --- | --- | --- | --- | --- | --- | | void QtGraphics::SimulationDone | ( | void |  | ) | `[signal]` | |

|  |  |
| --- | --- |
|  |  |

|  |  |  |  |  |  |  |
| --- | --- | --- | --- | --- | --- | --- |
| |  |  |  |  |  |  | | --- | --- | --- | --- | --- | --- | | virtual void QtGraphics::TimeStep | ( | void |  | ) | `[virtual]` | |

|  |  |
| --- | --- |
|  | Implement this member function in your simulation code. Include all actions that should be carried out during a simulation step, including PDE and CPM simulation steps. See the included examples (vessel.cpp, sorting.cpp) for more information. Reimplemented from Graphics. |

|  |  |  |  |  |  |  |
| --- | --- | --- | --- | --- | --- | --- |
| |  |  |  |  |  |  | | --- | --- | --- | --- | --- | --- | | void QtGraphics::TimeStepWrap | ( | void |  | ) | `[slot]` | |

|  |  |
| --- | --- |
|  |  |

|  |  |  |  |  |  |  |  |  |  |  |  |  |
| --- | --- | --- | --- | --- | --- | --- | --- | --- | --- | --- | --- | --- |
| |  |  |  |  | | --- | --- | --- | --- | | void QtGraphics::Write | ( | char \* | *fname*, | |  |  | int | *quality* = -1 | |  | ) | `[virtual]` | | |

|  |  |  |  |  |  |
| --- | --- | --- | --- | --- | --- |
|  | Writes the Image to a file. File format is inferred from file extension. Currently only PNG is supported by the X-Windows implementation; the Qt-implentation supports all formats supported by Qt. **Parameters:**  |  |  | | --- | --- | | *fname:* | File name with standard image file extension (e.g. png). | | *quality:* | Quality of JPEG images, defaults to -1 (no value provided). |  Implements Graphics. |

|  |  |  |  |  |  |  |
| --- | --- | --- | --- | --- | --- | --- |
| |  |  |  |  |  |  | | --- | --- | --- | --- | --- | --- | | virtual int QtGraphics::XField | ( | void |  | ) | const `[inline, virtual]` | |

|  |  |
| --- | --- |
|  | Returns the width of the Graphics window, in pixels. Reimplemented from Graphics. |

|  |  |  |  |  |  |  |
| --- | --- | --- | --- | --- | --- | --- |
| |  |  |  |  |  |  | | --- | --- | --- | --- | --- | --- | | virtual int QtGraphics::YField | ( | void |  | ) | const `[inline, virtual]` | |

|  |  |
| --- | --- |
|  | Returns the height of the Graphics window, in pixels. Reimplemented from Graphics. |

---

The documentation for this class was generated from the following files:

- /home/romer/TST0.1.3/qtgraph.h- /home/romer/TST0.1.3/qt3graph.cpp- /home/romer/TST0.1.3/qtgraph.cpp

---

Generated on Tue Dec 12 16:32:41 2006 for Tissue Simulation Toolkit by

1.3.5
