# Supplementary material for: Contact-Inhibited Chemotaxis in De Novo and Sprouting Blood-Vessel Growth
Source: PLoS Comput Biol. 2008 Sep 19;4(9):e1000163. doi: 10.1371/journal.pcbi.1000163 (PMC2528254; doi:10.1371/journal.pcbi.1000163)
Supplement: Protocol S1 — Tissue Simulation Toolkit v0.1.3. The source code for the software used for the simulations presented in this paper is also available from http://sourceforge.net/projects/tst. Installation: Unpack and compile according to the instructions given in the INSTALL file The code is written in C++ using the cross-platform (Windows, Mac, or Unix/Linux) library Qt (available from www.trolltech.com). (332 KB ZIP) [file pcbi.1000163.s002.zip › TST0.1.3/html/classX11Graphics.html]

Tissue Simulation Toolkit: X11Graphics class Reference

Main Page | Namespace List | Class Hierarchy | Class List | File List | Namespace Members | Class Members | File Members

# X11Graphics Class Reference

X-Windows implementation of Graphics interface.
More...

`#include <x11graph.h>`

Inheritance diagram for X11Graphics:

List of all members.

|  |
| --- |
|  |
| Public Member Functions | |
|  | X11Graphics (int xfield, int yfield, const char \*movie\_file=0) |
| virtual | ~X11Graphics (void) |
| virtual void | BeginScene (void) |
|  | BeginScene() must be called before calling drawing functions. |
| virtual void | EndScene (void) |
|  | EndScene() must be called to flush the drawing buffer and display the scene. |
| void | Flush (void) |
|  | Flushes scene to window. Normally called by EndScene(). |
| virtual void | Point (int color, int x, int y) |
|  | Plot a point in the Graphics window. |
| virtual void | Line (int x1, int y1, int x2, int y2, int colour) |
|  | Draws a line (obviously... :-). |
| void | Field (const int \*\*r, int mag=1) |
|  | Plots a field of values given by \*\*f, using color coding given by colormap file. |
| virtual int | GetXYCoo (int \*X, int \*Y) |
|  | Probes the Window for user interaction, with mouse or keyboard. |
| char \* | ChangeTitle (const char \*message) |
|  | Changes the title bar of the Graphics window. |
| void | RecoverTitle (void) |
|  | Recovers the title prior to the last call of ChangeTitle(). |
| LineType | CropSize (void) |
|  | Returns the upper left and lower right coordinates of the area occupied by cells. |
| Coordinate | ReplaceBeast (Coordinate old\_size, Coordinate new\_size) |
|  | This member function was part of functionality that enables interactive resizing of the Window and CPM field, and followed by interactive replacement of the Dish's contents. The current version of CPM does not contain such functionality. |
| virtual int | XField (void) const |
|  | Returns the width of the Graphics window, in pixels. |
| virtual int | YField (void) const |
|  | Returns the height of the Graphics window, in pixels. |
| virtual void | Write (char \*fname, int quality=-1) |
|  | Writes the Image to a file. |
| void | ClearImage (void) |
|  | Clears all pixels in the Image. |
| virtual void | TimeStep (void) |
|  | Implement this member function in your simulation code. |

---

## Detailed Description

X-Windows implementation of Graphics interface.

For API see documentation of base class Graphics.

Has a number extra features: see below.

---

## Constructor & Destructor Documentation

|  |  |  |  |  |  |  |  |  |  |  |  |  |  |  |  |  |
| --- | --- | --- | --- | --- | --- | --- | --- | --- | --- | --- | --- | --- | --- | --- | --- | --- |
| |  |  |  |  | | --- | --- | --- | --- | | X11Graphics::X11Graphics | ( | int | *xfield*, | |  |  | int | *yfield*, | |  |  | const char \* | *movie\_file* = 0 | |  | ) |  | | |

|  |  |
| --- | --- |
|  |  |

|  |  |  |  |  |  |  |
| --- | --- | --- | --- | --- | --- | --- |
| |  |  |  |  |  |  | | --- | --- | --- | --- | --- | --- | | X11Graphics::~X11Graphics | ( | void |  | ) | `[virtual]` | |

|  |  |
| --- | --- |
|  |  |

---

## Member Function Documentation

|  |  |  |  |  |  |  |
| --- | --- | --- | --- | --- | --- | --- |
| |  |  |  |  |  |  | | --- | --- | --- | --- | --- | --- | | void X11Graphics::BeginScene | ( | void |  | ) | `[virtual]` | |

|  |  |
| --- | --- |
|  | BeginScene() must be called before calling drawing functions. Reimplemented from Graphics. |

|  |  |  |  |  |  |  |
| --- | --- | --- | --- | --- | --- | --- |
| |  |  |  |  |  |  | | --- | --- | --- | --- | --- | --- | | char \* X11Graphics::ChangeTitle | ( | const char \* | *message* | ) |  | |

|  |  |  |  |
| --- | --- | --- | --- |
|  | Changes the title bar of the Graphics window. **Parameters:**  |  |  | | --- | --- | | *message:* | Text to display in title bar. | |

|  |  |  |  |  |  |  |
| --- | --- | --- | --- | --- | --- | --- |
| |  |  |  |  |  |  | | --- | --- | --- | --- | --- | --- | | void X11Graphics::ClearImage | ( | void |  | ) | `[inline]` | |

|  |  |
| --- | --- |
|  | Clears all pixels in the Image. |

|  |  |  |  |  |  |  |
| --- | --- | --- | --- | --- | --- | --- |
| |  |  |  |  |  |  | | --- | --- | --- | --- | --- | --- | | LineType X11Graphics::CropSize | ( | void |  | ) |  | |

|  |  |
| --- | --- |
|  | Returns the upper left and lower right coordinates of the area occupied by cells. **Returns:**  Bounding box as a LineType structure {int x1,int y1,int x2,int y2}. Warning: Assumes the window only displays cells (i.e. no PDE fields etc.). If you need this, better implement it as a member function of class CellularPotts. |

|  |  |  |  |  |  |  |
| --- | --- | --- | --- | --- | --- | --- |
| |  |  |  |  |  |  | | --- | --- | --- | --- | --- | --- | | void X11Graphics::EndScene | ( | void |  | ) | `[virtual]` | |

|  |  |
| --- | --- |
|  | EndScene() must be called to flush the drawing buffer and display the scene. Reimplemented from Graphics. |

|  |  |  |  |  |  |  |  |  |  |  |  |  |
| --- | --- | --- | --- | --- | --- | --- | --- | --- | --- | --- | --- | --- |
| |  |  |  |  | | --- | --- | --- | --- | | void X11Graphics::Field | ( | const int \*\* | *r*, | |  |  | int | *mag* = 1 | |  | ) | `[virtual]` | | |

|  |  |  |  |  |  |
| --- | --- | --- | --- | --- | --- |
|  | Plots a field of values given by \*\*f, using color coding given by colormap file. Only implemented in X11Graphics. No checks. Usage not recommended. **Parameters:**  |  |  | | --- | --- | | *f:* | Double pointer to array of integers, giving color indices using standard colormap ('default.ctb'). | | *mag:* | magnification factor. |  Reimplemented from Graphics. |

|  |  |  |  |  |  |  |
| --- | --- | --- | --- | --- | --- | --- |
| |  |  |  |  |  |  | | --- | --- | --- | --- | --- | --- | | void X11Graphics::Flush | ( | void |  | ) | `[inline]` | |

|  |  |
| --- | --- |
|  | Flushes scene to window. Normally called by EndScene(). |

|  |  |  |  |  |  |  |  |  |  |  |  |  |
| --- | --- | --- | --- | --- | --- | --- | --- | --- | --- | --- | --- | --- |
| |  |  |  |  | | --- | --- | --- | --- | | int X11Graphics::GetXYCoo | ( | int \* | *X*, | |  |  | int \* | *Y* | |  | ) | `[virtual]` | | |

|  |  |  |  |
| --- | --- | --- | --- |
|  | Probes the Window for user interaction, with mouse or keyboard. This function should return immediately, and return 0 if there was no user interaction. **Parameters:**  |  |  | | --- | --- | | *\*X,\*Y:* | Pointer where the clicked coordinate will be stored. |  Implements Graphics. |

|  |  |  |  |  |  |  |  |  |  |  |  |  |  |  |  |  |  |  |  |  |  |  |  |  |
| --- | --- | --- | --- | --- | --- | --- | --- | --- | --- | --- | --- | --- | --- | --- | --- | --- | --- | --- | --- | --- | --- | --- | --- | --- |
| |  |  |  |  | | --- | --- | --- | --- | | void X11Graphics::Line | ( | int | *x1*, | |  |  | int | *y1*, | |  |  | int | *x2*, | |  |  | int | *y2*, | |  |  | int | *colour* | |  | ) | `[virtual]` | | |

|  |  |  |  |  |  |  |  |
| --- | --- | --- | --- | --- | --- | --- | --- |
|  | Draws a line (obviously... :-). **Parameters:**  |  |  | | --- | --- | | *x1,y1:* | First coordinate pair. | | *x2,y2:* | Second coordinate pair. | | *color:* | Color of the line, as given in the colormap file "default.ctb". |  Implements Graphics. |

|  |  |  |  |  |  |  |  |  |  |  |  |  |  |  |  |  |
| --- | --- | --- | --- | --- | --- | --- | --- | --- | --- | --- | --- | --- | --- | --- | --- | --- |
| |  |  |  |  | | --- | --- | --- | --- | | void X11Graphics::Point | ( | int | *color*, | |  |  | int | *x*, | |  |  | int | *y* | |  | ) | `[virtual]` | | |

|  |  |  |  |  |  |
| --- | --- | --- | --- | --- | --- |
|  | Plot a point in the Graphics window. **Parameters:**  |  |  | | --- | --- | | *color:* | Color index, as defined in colormap file "default.ctb", which should be in the same directory as the executable. | | *x,y:* | Coordinate of point, in Graphics coordinates (typically twice as large as the cellular automata coordinates). |  Implements Graphics. |

|  |  |  |  |  |  |  |
| --- | --- | --- | --- | --- | --- | --- |
| |  |  |  |  |  |  | | --- | --- | --- | --- | --- | --- | | void X11Graphics::RecoverTitle | ( | void |  | ) |  | |

|  |  |
| --- | --- |
|  | Recovers the title prior to the last call of ChangeTitle(). |

|  |  |  |  |  |  |  |  |  |  |  |  |  |
| --- | --- | --- | --- | --- | --- | --- | --- | --- | --- | --- | --- | --- |
| |  |  |  |  | | --- | --- | --- | --- | | Coordinate X11Graphics::ReplaceBeast | ( | Coordinate | *old\_size*, | |  |  | Coordinate | *new\_size* | |  | ) |  | | |

|  |  |
| --- | --- |
|  | This member function was part of functionality that enables interactive resizing of the Window and CPM field, and followed by interactive replacement of the Dish's contents. The current version of CPM does not contain such functionality. |

|  |  |  |  |  |  |  |
| --- | --- | --- | --- | --- | --- | --- |
| |  |  |  |  |  |  | | --- | --- | --- | --- | --- | --- | | virtual void X11Graphics::TimeStep | ( | void |  | ) | `[virtual]` | |

|  |  |
| --- | --- |
|  | Implement this member function in your simulation code. Include all actions that should be carried out during a simulation step, including PDE and CPM simulation steps. See the included examples (vessel.cpp, sorting.cpp) for more information. Reimplemented from Graphics. |

|  |  |  |  |  |  |  |  |  |  |  |  |  |
| --- | --- | --- | --- | --- | --- | --- | --- | --- | --- | --- | --- | --- |
| |  |  |  |  | | --- | --- | --- | --- | | void X11Graphics::Write | ( | char \* | *fname*, | |  |  | int | *quality* = -1 | |  | ) | `[virtual]` | | |

|  |  |  |  |  |  |
| --- | --- | --- | --- | --- | --- |
|  | Writes the Image to a file. File format is inferred from file extension. Currently only PNG is supported by the X-Windows implementation; the Qt-implentation supports all formats supported by Qt. **Parameters:**  |  |  | | --- | --- | | *fname:* | File name with standard image file extension (e.g. png). | | *quality:* | Quality of JPEG images, defaults to -1 (no value provided). |  Implements Graphics. |

|  |  |  |  |  |  |  |
| --- | --- | --- | --- | --- | --- | --- |
| |  |  |  |  |  |  | | --- | --- | --- | --- | --- | --- | | virtual int X11Graphics::XField | ( | void |  | ) | const `[inline, virtual]` | |

|  |  |
| --- | --- |
|  | Returns the width of the Graphics window, in pixels. Reimplemented from Graphics. |

|  |  |  |  |  |  |  |
| --- | --- | --- | --- | --- | --- | --- |
| |  |  |  |  |  |  | | --- | --- | --- | --- | --- | --- | | virtual int X11Graphics::YField | ( | void |  | ) | const `[inline, virtual]` | |

|  |  |
| --- | --- |
|  | Returns the height of the Graphics window, in pixels. Reimplemented from Graphics. |

---

The documentation for this class was generated from the following files:

- /home/romer/TST0.1.3/x11graph.h- /home/romer/TST0.1.3/x11graph.cpp

---

Generated on Tue Dec 12 16:32:41 2006 for Tissue Simulation Toolkit by

1.3.5
