# Supplementary material for: Contact-Inhibited Chemotaxis in De Novo and Sprouting Blood-Vessel Growth
Source: PLoS Comput Biol. 2008 Sep 19;4(9):e1000163. doi: 10.1371/journal.pcbi.1000163 (PMC2528254; doi:10.1371/journal.pcbi.1000163)
Supplement: Protocol S1 — Tissue Simulation Toolkit v0.1.3. The source code for the software used for the simulations presented in this paper is also available from http://sourceforge.net/projects/tst. Installation: Unpack and compile according to the instructions given in the INSTALL file The code is written in C++ using the cross-platform (Windows, Mac, or Unix/Linux) library Qt (available from www.trolltech.com). (332 KB ZIP) [file pcbi.1000163.s002.zip › TST0.1.3/html/conrec_8h-source.html]

Tissue Simulation Toolkit: /home/romer/TST0.1.3/conrec.h Source File

Main Page | Namespace List | Class Hierarchy | Class List | File List | Namespace Members | Class Members | File Members

# /home/romer/TST0.1.3/conrec.h

Go to the documentation of this file.

```
00001 /* 
00002 
00003 Copyright 1996-2006 Roeland Merks
00004 
00005 This file is part of Tissue Simulation Toolkit.
00006 
00007 Tissue Simulation Toolkit is free software; you can redistribute
00008 it and/or modify it under the terms of the GNU General Public
00009 License as published by the Free Software Foundation; either
00010 version 2 of the License, or (at your option) any later version.
00011 
00012 Tissue Simulation Toolkit is distributed in the hope that it will
00013 be useful, but WITHOUT ANY WARRANTY; without even the implied
00014 warranty of MERCHANTABILITY or FITNESS FOR A PARTICULAR PURPOSE.
00015 See the GNU General Public License for more details.
00016 
00017 You should have received a copy of the GNU General Public License
00018 along with Tissue Simulation Toolkit; if not, write to the Free
00019 Software Foundation, Inc., 51 Franklin St, Fifth Floor, Boston, MA
00020 02110-1301 USA
00021 
00022 */
00023 #ifndef _CONREC_H_
00024 #define _CONREC_H_
00025 /*
00026 Copyright (c) 1996-1997 Nicholas Yue
00027 
00028 This software is copyrighted by Nicholas Yue. This code is base on the work of
00029 Paul D. Bourke CONREC.F routine
00030 
00031 The authors hereby grant permission to use, copy, and distribute this
00032 software and its documentation for any purpose, provided that existing
00033 copyright notices are retained in all copies and that this notice is included
00034 verbatim in any distributions. Additionally, the authors grant permission to
00035 modify this software and its documentation for any purpose, provided that
00036 such modifications are not distributed without the explicit consent of the
00037 authors and that existing copyright notices are retained in all copies. Some
00038 of the algorithms implemented by this software are patented, observe all
00039 applicable patent law.
00040 
00041 IN NO EVENT SHALL THE AUTHORS OR DISTRIBUTORS BE LIABLE TO ANY PARTY FOR
00042 DIRECT, INDIRECT, SPECIAL, INCIDENTAL, OR CONSEQUENTIAL DAMAGES ARISING OUT
00043 OF THE USE OF THIS SOFTWARE, ITS DOCUMENTATION, OR ANY DERIVATIVES THEREOF,
00044 EVEN IF THE AUTHORS HAVE BEEN ADVISED OF THE POSSIBILITY OF SUCH DAMAGE.
00045 
00046 THE AUTHORS AND DISTRIBUTORS SPECIFICALLY DISCLAIM ANY WARRANTIES, INCLUDING,
00047 BUT NOT LIMITED TO, THE IMPLIED WARRANTIES OF MERCHANTABILITY, FITNESS FOR A
00048 PARTICULAR PURPOSE, AND NON-INFRINGEMENT.  THIS SOFTWARE IS PROVIDED ON AN
00049 "AS IS" BASIS, AND THE AUTHORS AND DISTRIBUTORS HAVE NO OBLIGATION TO PROVIDE
00050 MAINTENANCE, SUPPORT, UPDATES, ENHANCEMENTS, OR MODIFICATIONS.
00051 */
00052 
00053 //=============================================================================
00054 //
00055 //     CONREC is a contouring subroutine for rectangularily spaced data.
00056 //
00057 //     It emits calls to a line drawing subroutine supplied by the user
00058 //     which draws a contour map corresponding to real*4data on a randomly
00059 //     spaced rectangular grid. The coordinates emitted are in the same
00060 //     units given in the x() and y() arrays.
00061 //
00062 //     Any number of contour levels may be specified but they must be
00063 //     in order of increasing value.
00064 //
00065 //     As this code is ported from FORTRAN-77, please be very careful of the
00066 //     various indices like ilb,iub,jlb and jub, remeber that C/C++ indices
00067 //     starts from zero (0)
00068 //
00069 //=============================================================================
00070 
00071 class Graphics;
00072 
00073 
00078 int conrec(double **d,
00079            int ilb,
00080            int iub,
00081            int jlb,
00082            int jub,
00083            double *x,
00084            double *y,
00085            int nc,
00086            double *z,
00087            Graphics *g,
00088            int colour=1);
00089 // d               ! matrix of data to contour
00090 // ilb,iub,jlb,jub ! index bounds of data matrix
00091 // x               ! data matrix column coordinates
00092 // y               ! data matrix row coordinates
00093 // nc              ! number of contour levels
00094 // z               ! contour levels in increasing order
00095 
00096 #endif
```

---

Generated on Tue Dec 12 16:32:40 2006 for Tissue Simulation Toolkit by

1.3.5 
