# Supplementary material for: Contact-Inhibited Chemotaxis in De Novo and Sprouting Blood-Vessel Growth
Source: PLoS Comput Biol. 2008 Sep 19;4(9):e1000163. doi: 10.1371/journal.pcbi.1000163 (PMC2528254; doi:10.1371/journal.pcbi.1000163)
Supplement: Protocol S1 — Tissue Simulation Toolkit v0.1.3. The source code for the software used for the simulations presented in this paper is also available from http://sourceforge.net/projects/tst. Installation: Unpack and compile according to the instructions given in the INSTALL file The code is written in C++ using the cross-platform (Windows, Mac, or Unix/Linux) library Qt (available from www.trolltech.com). (332 KB ZIP) [file pcbi.1000163.s002.zip › TST0.1.3/html/conrec_8h.html]

Tissue Simulation Toolkit: conrec.h File Reference

Main Page | Namespace List | Class Hierarchy | Class List | File List | Namespace Members | Class Members | File Members

# /home/romer/TST0.1.3/conrec.h File Reference

Go to the source code of this file.

|  |
| --- |
|  |
| Functions | |
| int | conrec (double \*\*d, int ilb, int iub, int jlb, int jub, double \*x, double \*y, int nc, double \*z, Graphics \*g, int colour=1) |
|  | Paul Bourke's conrec algorithm to draw contour lines of PDE fields. |

---

## Function Documentation

|  |  |  |  |  |  |  |  |  |  |  |  |  |  |  |  |  |  |  |  |  |  |  |  |  |  |  |  |  |  |  |  |  |  |  |  |  |  |  |  |  |  |  |  |  |  |  |  |  |
| --- | --- | --- | --- | --- | --- | --- | --- | --- | --- | --- | --- | --- | --- | --- | --- | --- | --- | --- | --- | --- | --- | --- | --- | --- | --- | --- | --- | --- | --- | --- | --- | --- | --- | --- | --- | --- | --- | --- | --- | --- | --- | --- | --- | --- | --- | --- | --- | --- |
| |  |  |  |  | | --- | --- | --- | --- | | int conrec | ( | double \*\* | *d*, | |  |  | int | *ilb*, | |  |  | int | *iub*, | |  |  | int | *jlb*, | |  |  | int | *jub*, | |  |  | double \* | *x*, | |  |  | double \* | *y*, | |  |  | int | *nc*, | |  |  | double \* | *z*, | |  |  | Graphics \* | *g*, | |  |  | int | *colour* = 1 | |  | ) |  | | |

|  |  |
| --- | --- |
|  | Paul Bourke's conrec algorithm to draw contour lines of PDE fields. C-code Copyright (c) 1996-1997 Nicholas Yue. |

---

Generated on Tue Dec 12 16:32:41 2006 for Tissue Simulation Toolkit by

1.3.5
