# Supplementary material for: Contact-Inhibited Chemotaxis in De Novo and Sprouting Blood-Vessel Growth
Source: PLoS Comput Biol. 2008 Sep 19;4(9):e1000163. doi: 10.1371/journal.pcbi.1000163 (PMC2528254; doi:10.1371/journal.pcbi.1000163)
Supplement: Protocol S1 — Tissue Simulation Toolkit v0.1.3. The source code for the software used for the simulations presented in this paper is also available from http://sourceforge.net/projects/tst. Installation: Unpack and compile according to the instructions given in the INSTALL file The code is written in C++ using the cross-platform (Windows, Mac, or Unix/Linux) library Qt (available from www.trolltech.com). (332 KB ZIP) [file pcbi.1000163.s002.zip › TST0.1.3/html/crash_8h-source.html]

Tissue Simulation Toolkit: /home/romer/TST0.1.3/crash.h Source File

Main Page | Namespace List | Class Hierarchy | Class List | File List | Namespace Members | Class Members | File Members

# /home/romer/TST0.1.3/crash.h

Go to the documentation of this file.

```
00001 /* 
00002 
00003 Copyright 1996-2006 Roeland Merks
00004 
00005 This file is part of Tissue Simulation Toolkit.
00006 
00007 Tissue Simulation Toolkit is free software; you can redistribute
00008 it and/or modify it under the terms of the GNU General Public
00009 License as published by the Free Software Foundation; either
00010 version 2 of the License, or (at your option) any later version.
00011 
00012 Tissue Simulation Toolkit is distributed in the hope that it will
00013 be useful, but WITHOUT ANY WARRANTY; without even the implied
00014 warranty of MERCHANTABILITY or FITNESS FOR A PARTICULAR PURPOSE.
00015 See the GNU General Public License for more details.
00016 
00017 You should have received a copy of the GNU General Public License
00018 along with Tissue Simulation Toolkit; if not, write to the Free
00019 Software Foundation, Inc., 51 Franklin St, Fifth Floor, Boston, MA
00020 02110-1301 USA
00021 
00022 */
00023 void StartSIGINTHandling();
00024 void HandleSIGINT(int dummy);
00025 void StartSIGSEGVHandling();
00026 void HandleSIGSEGV(int dummy);
00027 void NiceMessage();
00028 void MemoryWarning(void);
00029 void Crash(char *message);
```

---

Generated on Tue Dec 12 16:32:40 2006 for Tissue Simulation Toolkit by

1.3.5 
