# Supplementary material for: Contact-Inhibited Chemotaxis in De Novo and Sprouting Blood-Vessel Growth
Source: PLoS Comput Biol. 2008 Sep 19;4(9):e1000163. doi: 10.1371/journal.pcbi.1000163 (PMC2528254; doi:10.1371/journal.pcbi.1000163)
Supplement: Protocol S1 — Tissue Simulation Toolkit v0.1.3. The source code for the software used for the simulations presented in this paper is also available from http://sourceforge.net/projects/tst. Installation: Unpack and compile according to the instructions given in the INSTALL file The code is written in C++ using the cross-platform (Windows, Mac, or Unix/Linux) library Qt (available from www.trolltech.com). (332 KB ZIP) [file pcbi.1000163.s002.zip › TST0.1.3/html/dish_8h-source.html]

Tissue Simulation Toolkit: /home/romer/TST0.1.3/dish.h Source File

Main Page | Namespace List | Class Hierarchy | Class List | File List | Namespace Members | Class Members | File Members

# /home/romer/TST0.1.3/dish.h

Go to the documentation of this file.

```
00001 /* 
00002 
00003 Copyright 1996-2006 Roeland Merks
00004 
00005 This file is part of Tissue Simulation Toolkit.
00006 
00007 Tissue Simulation Toolkit is free software; you can redistribute
00008 it and/or modify it under the terms of the GNU General Public
00009 License as published by the Free Software Foundation; either
00010 version 2 of the License, or (at your option) any later version.
00011 
00012 Tissue Simulation Toolkit is distributed in the hope that it will
00013 be useful, but WITHOUT ANY WARRANTY; without even the implied
00014 warranty of MERCHANTABILITY or FITNESS FOR A PARTICULAR PURPOSE.
00015 See the GNU General Public License for more details.
00016 
00017 You should have received a copy of the GNU General Public License
00018 along with Tissue Simulation Toolkit; if not, write to the Free
00019 Software Foundation, Inc., 51 Franklin St, Fifth Floor, Boston, MA
00020 02110-1301 USA
00021 
00022 */
00023 
00030 #ifndef CRITTER_H_
00031 #define CRITTER_H_
00032 #include <vector>
00033 #include "graph.h"
00034 #include "random.h"
00035 #include "pde.h"
00036 #include "cell.h"
00037 #include "ca.h"
00038 
00039 namespace ColourMode {
00040   enum { State,CellType,Sigma,Auxilliary };
00041 }
00042 
00043 class Dish {
00044 
00045   friend class Info;
00046 
00047 public:
00048   Dish(void);
00049   
00057   void Init(void);
00058 
00059   void ConstructorBody(void);
00060   
00061   virtual ~Dish();
00062  
00067   void Plot(Graphics *g);
00068 
00069   
00070   int ZygoteArea(void) const;
00071   
00073   int Time(void) const;
00074   
00076   int CountCells(void) const;
00077   
00084   void CellGrowthAndDivision(void);
00085   
00087   int Area(void) const;
00088 
00090   int TargetArea(void) const;
00091 
00093   int SizeX(void);
00094   
00096   int SizeY(void);
00097 
00099   inline Cell &getCell(int c) {
00100     return cell[c];
00101   }
00102   
00103   PDE *PDEfield;
00104   CellularPotts *CPM;
00105 
00106   // Was used for gradient measurements, not functional now.
00107   void ClearGrads(void);
00108 
00109   void MeasureChemConcentrations(void);
00110 protected:
00112   void SetCellOwner(Cell &which_cell);
00113 
00114 private:
00115   bool CellLonelyP(const Cell &c, int **neighbours) const;
00116 
00117 protected:
00119   std::vector<Cell> cell;
00120 };
00121 
00122 #define INIT void Dish::Init(void)
00123 
00124 #endif
```

---

Generated on Tue Dec 12 16:32:40 2006 for Tissue Simulation Toolkit by

1.3.5 
