# Supplementary material for: Contact-Inhibited Chemotaxis in De Novo and Sprouting Blood-Vessel Growth
Source: PLoS Comput Biol. 2008 Sep 19;4(9):e1000163. doi: 10.1371/journal.pcbi.1000163 (PMC2528254; doi:10.1371/journal.pcbi.1000163)
Supplement: Protocol S1 — Tissue Simulation Toolkit v0.1.3. The source code for the software used for the simulations presented in this paper is also available from http://sourceforge.net/projects/tst. Installation: Unpack and compile according to the instructions given in the INSTALL file The code is written in C++ using the cross-platform (Windows, Mac, or Unix/Linux) library Qt (available from www.trolltech.com). (332 KB ZIP) [file pcbi.1000163.s002.zip › TST0.1.3/html/files.html]

Tissue Simulation Toolkit: File Index

Main Page | Namespace List | Class Hierarchy | Class List | File List | Namespace Members | Class Members | File Members

# Tissue Simulation Toolkit File List

Here is a list of all files with brief descriptions:

|  |  |
| --- | --- |
| /home/romer/TST0.1.3/ca.cpp |  |
| /home/romer/TST0.1.3/ca.h [code] |  |
| /home/romer/TST0.1.3/cell.cpp |  |
| /home/romer/TST0.1.3/cell.h [code] |  |
| /home/romer/TST0.1.3/conrec.cpp |  |
| /home/romer/TST0.1.3/conrec.h [code] |  |
| /home/romer/TST0.1.3/crash.cpp |  |
| /home/romer/TST0.1.3/crash.h [code] |  |
| /home/romer/TST0.1.3/dish.cpp |  |
| /home/romer/TST0.1.3/dish.h [code] |  |
| /home/romer/TST0.1.3/engulfment.cpp |  |
| /home/romer/TST0.1.3/graph.h [code] |  |
| /home/romer/TST0.1.3/hull.cpp |  |
| /home/romer/TST0.1.3/hull.h [code] |  |
| /home/romer/TST0.1.3/info.cpp |  |
| /home/romer/TST0.1.3/info.h [code] |  |
| /home/romer/TST0.1.3/mainpage.h [code] |  |
| /home/romer/TST0.1.3/misc.cpp |  |
| /home/romer/TST0.1.3/misc.h [code] |  |
| /home/romer/TST0.1.3/output.cpp |  |
| /home/romer/TST0.1.3/output.h [code] |  |
| /home/romer/TST0.1.3/parameter.cpp |  |
| /home/romer/TST0.1.3/parameter.h [code] |  |
| /home/romer/TST0.1.3/parse.cpp |  |
| /home/romer/TST0.1.3/parse.h [code] |  |
| /home/romer/TST0.1.3/pde.cpp |  |
| /home/romer/TST0.1.3/pde.h [code] |  |
| /home/romer/TST0.1.3/pushing.cpp |  |
| /home/romer/TST0.1.3/qt3graph.cpp |  |
| /home/romer/TST0.1.3/qtgraph.cpp |  |
| /home/romer/TST0.1.3/qtgraph.h [code] |  |
| /home/romer/TST0.1.3/random.cpp |  |
| /home/romer/TST0.1.3/random.h [code] |  |
| /home/romer/TST0.1.3/sorting.cpp |  |
| /home/romer/TST0.1.3/sqr.h [code] |  |
| /home/romer/TST0.1.3/sticky.h [code] |  |
| /home/romer/TST0.1.3/tumor.cpp |  |
| /home/romer/TST0.1.3/vessel.cpp |  |
| /home/romer/TST0.1.3/warning.cpp |  |
| /home/romer/TST0.1.3/warning.h [code] |  |
| /home/romer/TST0.1.3/x11graph.cpp |  |
| /home/romer/TST0.1.3/x11graph.h [code] |  |

---

Generated on Tue Dec 12 16:32:40 2006 for Tissue Simulation Toolkit by

1.3.5 
