# Supplementary material for: Contact-Inhibited Chemotaxis in De Novo and Sprouting Blood-Vessel Growth
Source: PLoS Comput Biol. 2008 Sep 19;4(9):e1000163. doi: 10.1371/journal.pcbi.1000163 (PMC2528254; doi:10.1371/journal.pcbi.1000163)
Supplement: Protocol S1 — Tissue Simulation Toolkit v0.1.3. The source code for the software used for the simulations presented in this paper is also available from http://sourceforge.net/projects/tst. Installation: Unpack and compile according to the instructions given in the INSTALL file The code is written in C++ using the cross-platform (Windows, Mac, or Unix/Linux) library Qt (available from www.trolltech.com). (332 KB ZIP) [file pcbi.1000163.s002.zip › TST0.1.3/html/functions.html]

Tissue Simulation Toolkit: Compound Member Index

Main Page | Namespace List | Class Hierarchy | Class List | File List | Namespace Members | Class Members | File Members

All | Functions | Variables | Related Functions

a | b | c | d | e | f | g | i | j | l | m | n | o | p | q | r | s | t | v | w | x | y | z | ~

Here is a list of all class members with links to the classes they belong to:

### - a -

- aa1
  : Dir- aa2
    : Dir- AbsorbingBoundaries()
      : PDE- AddCell()
        : CellularPotts- AddToGrad()
          : Cell- addtoValue()
            : PDE- alive
              : Cell- AliveP()
                : Cell- AllocateSigma()
                  : PDE, CellularPotts- alt\_sigma
                    : PDE- AmoebaeMove()
                      : CellularPotts- amount
                        : Cell- Apoptose()
                          : Cell- area
                            : Cell- Area()
                              : Dish, Cell

### - b -

- BaseInitialisation()
  : CellularPotts- bb1
    : Dir- bb2
      : Dir- BeginScene()
        : X11Graphics, QtGraphics, Graphics- border\_energy
          : Parameter

### - c -

- capacity
  : Cell- cell
    : Dish- Cell()
      : Cell- CellDensity()
        : CellularPotts- CellGrowthAndDivision()
          : Dish- CellularPotts
            : CellularPotts, Cell, Dir- ChangeTitle()
              : X11Graphics- chem
                : Cell- chemotaxis
                  : Parameter- CleanUp()
                    : Parameter- ClearGrad()
                      : Cell- ClearGrads()
                        : Dish- ClearImage()
                          : X11Graphics, QtGraphics- ClearJ()
                            : Cell- ClickCell()
                              : Info- Colour()
                                : Cell- colour
                                  : Cell- colour\_of\_birth
                                    : Cell- ColourOfBirth()
                                      : Cell- Compactness()
                                        : CellularPotts- conn\_diss
                                          : Parameter- ConstructInitCells()
                                            : CellularPotts- ConstructorBody()
                                              : Dish- ContourPlot()
                                                : PDE- CountCells()
                                                  : Dish- CPM
                                                    : Dish- CropSize()
                                                      : X11Graphics

### - d -

- datadir
  : Parameter- date\_of\_birth
    : Cell- DateOfBirth()
      : Cell- daughter
        : Cell- Daughter()
          : Cell- decay\_rate
            : Parameter- DecrementTargetArea()
              : Cell- diff\_coeff
                : Parameter- Diffuse()
                  : PDE- Dir()
                    : Dir- Dish
                      : Dish, Cell- DivideCells()
                        : CellularPotts- divisions
                          : Parameter- DrawConvexHull()
                            : CellularPotts- dt
                              : Parameter- dx
                                : Parameter

### - e -

- EndScene()
  : X11Graphics, QtGraphics, Graphics- EnergyDifference()
    : Cell- extensiononly
      : Parameter

### - f -

- Field()
  : X11Graphics, Graphics- FindCellDirections()
    : CellularPotts- Flush()
      : X11Graphics

### - g -

- getCell()
  : Dish, CellularPotts- GetChemAmount()
    : PDE- GetGrad()
      : Cell- GetJ()
        : Cell- getTau()
          : Cell- GetXYCoo()
            : X11Graphics, QtGraphics, Graphics- grad
              : Cell- GradC()
                : PDE- GradX()
                  : Cell- GradY()
                    : Cell- graphics
                      : Parameter- GrowAndDivideCells()
                        : CellularPotts- GrowInCells()
                          : CellularPotts- growth\_threshold
                            : Cell

### - i -

- IncrementTargetArea()
  : Cell- Info
    : Info, PDE, Dish, Cell, CellularPotts- Init()
      : Dish

### - j -

- J
  : Cell- Jtable
    : Parameter

### - l -

- lambda
  : Parameter- lambda2
    : Parameter- Layers()
      : PDE- layers
        : PDE- lb1
          : Dir- lb2
            : Dir- Length()
              : Cell- length
                : Cell- Line()
                  : X11Graphics, QtGraphics, Graphics

### - m -

- MapColour()
  : PDE- Mass()
    : CellularPotts- Max()
      : PDE- maxsigma
        : Cell- MaxSigma()
          : Cell- maxtau
            : Cell- mcs
              : Parameter- MeanCellArea()
                : CellularPotts- MeasureCellSize()
                  : Cell- MeasureChemConcentrations()
                    : Dish- Menu()
                      : Info- Min()
                        : PDE- Morphometry
                          : CellularPotts- mother
                            : Cell- Mother()
                              : Cell

### - n -

- n\_chem
  : Parameter- n\_copies
    : Cell- n\_init\_cells
      : Parameter- neighbours
        : Parameter- NoFluxBoundaries()
          : PDE

### - o -

- operator=()
  : Cell- owner
    : Cell

### - p -

- Parameter()
  : Parameter- PDE()
    : PDE- pde\_its
      : Parameter- PDEfield
        : Dish- periodic\_boundaries
          : Parameter- PeriodicBoundaries()
            : PDE- Plot()
              : PDE, Dish, CellularPotts- PlotSigma()
                : CellularPotts- PlotVectorField()
                  : PDE- Point()
                    : X11Graphics, QtGraphics, Point, Graphics- polarvec
                      : Cell- PrintInertia()
                        : Cell

### - q -

- QtGraphics()
  : QtGraphics

### - r -

- Read()
  : Parameter- ReadZygotePicture()
    : CellularPotts- RecoverTitle()
      : X11Graphics- relaxation
        : Parameter- RenormPolarVec()
          : Cell- Replace()
            : CellularPotts- ReplaceBeast()
              : X11Graphics- ResetTargetLengths()
                : CellularPotts- rseed
                  : Parameter

### - s -

- saturation
  : Parameter- SearchNandPlot()
    : CellularPotts- SearchNeighbours()
      : CellularPotts- secr\_rate
        : Parameter- Secrete()
          : PDE- SetCellOwner()
            : Dish- SetColour()
              : Cell- SetGrad()
                : Cell- SetJ()
                  : Cell- SetRandomTypes()
                    : CellularPotts- SetTargetArea()
                      : Cell- SetTargetLength()
                        : Cell- setTau()
                          : Cell- setValue()
                            : PDE- ShowDirections()
                              : CellularPotts- Sigma()
                                : PDE, Cell, CellularPotts- sigma
                                  : PDE, Cell, CellularPotts- SimulationDone()
                                    : QtGraphics- size\_init\_cells
                                      : Parameter- SizeX()
                                        : PDE, Dish, CellularPotts- sizex
                                          : PDE, Parameter, CellularPotts- SizeY()
                                            : PDE, Dish, CellularPotts- sizey
                                              : PDE, Parameter, CellularPotts- spins\_converted
                                                : CellularPotts- storage\_stride
                                                  : Parameter- store
                                                    : Parameter- subfield
                                                      : Parameter- sum\_x
                                                        : Cell- sum\_xx
                                                          : Cell- sum\_xy
                                                            : Cell- sum\_y
                                                              : Cell- sum\_yy
                                                                : Cell

### - t -

- T
  : Parameter- target\_area
    : Parameter, Cell- target\_length
      : Parameter, Cell- TargetArea()
        : Dish, Cell- TargetLength()
          : Cell- tau
            : Cell- TheTime()
              : PDE- ThrowInCells()
                : CellularPotts- Time()
                  : Dish, CellularPotts- times\_divided
                    : Cell- TimesDivided()
                      : Cell- TimeStep()
                        : X11Graphics, QtGraphics, Graphics- TimeStepWrap()
                          : QtGraphics

### - v -

- v
  : Cell- vecadherinknockout
    : Parameter

### - w -

- Write()
  : X11Graphics, QtGraphics, Parameter, Graphics- WriteCOM()
    : Info

### - x -

- x
  : co, Point- x1
    : li- X11Graphics()
      : X11Graphics- x2
        : li- XField()
          : X11Graphics, QtGraphics, Graphics

### - y -

- y
  : co, Point- y1
    : li- y2
      : li- YField()
        : X11Graphics, QtGraphics, Graphics

### - z -

- ZygoteArea()
  : Dish, CellularPotts

### - ~ -

- ~Cell()
  : Cell- ~CellularPotts()
    : CellularPotts- ~Dish()
      : Dish- ~Graphics()
        : Graphics- ~Parameter()
          : Parameter- ~PDE()
            : PDE- ~QtGraphics()
              : QtGraphics- ~X11Graphics()
                : X11Graphics

---

Generated on Tue Dec 12 16:32:41 2006 for Tissue Simulation Toolkit by

1.3.5
