# Supplementary material for: Contact-Inhibited Chemotaxis in De Novo and Sprouting Blood-Vessel Growth
Source: PLoS Comput Biol. 2008 Sep 19;4(9):e1000163. doi: 10.1371/journal.pcbi.1000163 (PMC2528254; doi:10.1371/journal.pcbi.1000163)
Supplement: Protocol S1 — Tissue Simulation Toolkit v0.1.3. The source code for the software used for the simulations presented in this paper is also available from http://sourceforge.net/projects/tst. Installation: Unpack and compile according to the instructions given in the INSTALL file The code is written in C++ using the cross-platform (Windows, Mac, or Unix/Linux) library Qt (available from www.trolltech.com). (332 KB ZIP) [file pcbi.1000163.s002.zip › TST0.1.3/html/functions_func.html]

Tissue Simulation Toolkit: Compound Member Index

Main Page | Namespace List | Class Hierarchy | Class List | File List | Namespace Members | Class Members | File Members

All | Functions | Variables | Related Functions

a | b | c | d | e | f | g | i | l | m | n | o | p | q | r | s | t | w | x | y | z | ~

### - a -

- AbsorbingBoundaries()
  : PDE- AddCell()
    : CellularPotts- AddToGrad()
      : Cell- addtoValue()
        : PDE- AliveP()
          : Cell- AllocateSigma()
            : PDE, CellularPotts- AmoebaeMove()
              : CellularPotts- Apoptose()
                : Cell- Area()
                  : Dish, Cell

### - b -

- BaseInitialisation()
  : CellularPotts- BeginScene()
    : X11Graphics, QtGraphics, Graphics

### - c -

- Cell()
  : Cell- CellDensity()
    : CellularPotts- CellGrowthAndDivision()
      : Dish- CellularPotts()
        : CellularPotts, Cell, Dir- ChangeTitle()
          : X11Graphics- CleanUp()
            : Parameter- ClearGrad()
              : Cell- ClearGrads()
                : Dish- ClearImage()
                  : X11Graphics, QtGraphics- ClearJ()
                    : Cell- ClickCell()
                      : Info- Colour()
                        : Cell- ColourOfBirth()
                          : Cell- Compactness()
                            : CellularPotts- ConstructInitCells()
                              : CellularPotts- ConstructorBody()
                                : Dish- ContourPlot()
                                  : PDE- CountCells()
                                    : Dish- CropSize()
                                      : X11Graphics

### - d -

- DateOfBirth()
  : Cell- Daughter()
    : Cell- DecrementTargetArea()
      : Cell- Diffuse()
        : PDE- Dir()
          : Dir- Dish()
            : Dish, Cell- DivideCells()
              : CellularPotts- DrawConvexHull()
                : CellularPotts

### - e -

- EndScene()
  : X11Graphics, QtGraphics, Graphics- EnergyDifference()
    : Cell

### - f -

- Field()
  : X11Graphics, Graphics- FindCellDirections()
    : CellularPotts- Flush()
      : X11Graphics

### - g -

- getCell()
  : Dish, CellularPotts- GetChemAmount()
    : PDE- GetGrad()
      : Cell- GetJ()
        : Cell- getTau()
          : Cell- GetXYCoo()
            : X11Graphics, QtGraphics, Graphics- GradC()
              : PDE- GradX()
                : Cell- GradY()
                  : Cell- GrowAndDivideCells()
                    : CellularPotts- GrowInCells()
                      : CellularPotts

### - i -

- IncrementTargetArea()
  : Cell- Info()
    : Info, PDE, Dish, Cell, CellularPotts- Init()
      : Dish

### - l -

- Layers()
  : PDE- Length()
    : Cell- Line()
      : X11Graphics, QtGraphics, Graphics

### - m -

- MapColour()
  : PDE- Mass()
    : CellularPotts- Max()
      : PDE- MaxSigma()
        : Cell- MeanCellArea()
          : CellularPotts- MeasureCellSize()
            : Cell- MeasureChemConcentrations()
              : Dish- Menu()
                : Info- Min()
                  : PDE- Mother()
                    : Cell

### - n -

- NoFluxBoundaries()
  : PDE

### - o -

- operator=()
  : Cell

### - p -

- Parameter()
  : Parameter- PDE()
    : PDE- PeriodicBoundaries()
      : PDE- Plot()
        : PDE, Dish, CellularPotts- PlotSigma()
          : CellularPotts- PlotVectorField()
            : PDE- Point()
              : X11Graphics, QtGraphics, Point, Graphics- PrintInertia()
                : Cell

### - q -

- QtGraphics()
  : QtGraphics

### - r -

- Read()
  : Parameter- ReadZygotePicture()
    : CellularPotts- RecoverTitle()
      : X11Graphics- RenormPolarVec()
        : Cell- Replace()
          : CellularPotts- ReplaceBeast()
            : X11Graphics- ResetTargetLengths()
              : CellularPotts

### - s -

- SearchNandPlot()
  : CellularPotts- SearchNeighbours()
    : CellularPotts- Secrete()
      : PDE- SetCellOwner()
        : Dish- SetColour()
          : Cell- SetGrad()
            : Cell- SetJ()
              : Cell- SetRandomTypes()
                : CellularPotts- SetTargetArea()
                  : Cell- SetTargetLength()
                    : Cell- setTau()
                      : Cell- setValue()
                        : PDE- ShowDirections()
                          : CellularPotts- Sigma()
                            : PDE, Cell, CellularPotts- SimulationDone()
                              : QtGraphics- SizeX()
                                : PDE, Dish, CellularPotts- SizeY()
                                  : PDE, Dish, CellularPotts

### - t -

- TargetArea()
  : Dish, Cell- TargetLength()
    : Cell- TheTime()
      : PDE- ThrowInCells()
        : CellularPotts- Time()
          : Dish, CellularPotts- TimesDivided()
            : Cell- TimeStep()
              : X11Graphics, QtGraphics, Graphics- TimeStepWrap()
                : QtGraphics

### - w -

- Write()
  : X11Graphics, QtGraphics, Parameter, Graphics- WriteCOM()
    : Info

### - x -

- X11Graphics()
  : X11Graphics- XField()
    : X11Graphics, QtGraphics, Graphics

### - y -

- YField()
  : X11Graphics, QtGraphics, Graphics

### - z -

- ZygoteArea()
  : Dish, CellularPotts

### - ~ -

- ~Cell()
  : Cell- ~CellularPotts()
    : CellularPotts- ~Dish()
      : Dish- ~Graphics()
        : Graphics- ~Parameter()
          : Parameter- ~PDE()
            : PDE- ~QtGraphics()
              : QtGraphics- ~X11Graphics()
                : X11Graphics

---

Generated on Tue Dec 12 16:32:41 2006 for Tissue Simulation Toolkit by

1.3.5
