# Supplementary material for: Contact-Inhibited Chemotaxis in De Novo and Sprouting Blood-Vessel Growth
Source: PLoS Comput Biol. 2008 Sep 19;4(9):e1000163. doi: 10.1371/journal.pcbi.1000163 (PMC2528254; doi:10.1371/journal.pcbi.1000163)
Supplement: Protocol S1 — Tissue Simulation Toolkit v0.1.3. The source code for the software used for the simulations presented in this paper is also available from http://sourceforge.net/projects/tst. Installation: Unpack and compile according to the instructions given in the INSTALL file The code is written in C++ using the cross-platform (Windows, Mac, or Unix/Linux) library Qt (available from www.trolltech.com). (332 KB ZIP) [file pcbi.1000163.s002.zip › TST0.1.3/html/functions_vars.html]

Tissue Simulation Toolkit: Compound Member Index

Main Page | Namespace List | Class Hierarchy | Class List | File List | Namespace Members | Class Members | File Members

All | Functions | Variables | Related Functions

a | b | c | d | e | g | j | l | m | n | o | p | r | s | t | v | x | y

### - a -

- aa1
  : Dir- aa2
    : Dir- alive
      : Cell- alt\_sigma
        : PDE- amount
          : Cell- area
            : Cell

### - b -

- bb1
  : Dir- bb2
    : Dir- border\_energy
      : Parameter

### - c -

- capacity
  : Cell- cell
    : Dish- chem
      : Cell- chemotaxis
        : Parameter- colour
          : Cell- colour\_of\_birth
            : Cell- conn\_diss
              : Parameter- CPM
                : Dish

### - d -

- datadir
  : Parameter- date\_of\_birth
    : Cell- daughter
      : Cell- decay\_rate
        : Parameter- diff\_coeff
          : Parameter- divisions
            : Parameter- dt
              : Parameter- dx
                : Parameter

### - e -

- extensiononly
  : Parameter

### - g -

- grad
  : Cell- graphics
    : Parameter- growth\_threshold
      : Cell

### - j -

- J
  : Cell- Jtable
    : Parameter

### - l -

- lambda
  : Parameter- lambda2
    : Parameter- layers
      : PDE- lb1
        : Dir- lb2
          : Dir- length
            : Cell

### - m -

- maxsigma
  : Cell- maxtau
    : Cell- mcs
      : Parameter- mother
        : Cell

### - n -

- n\_chem
  : Parameter- n\_copies
    : Cell- n\_init\_cells
      : Parameter- neighbours
        : Parameter

### - o -

- owner
  : Cell

### - p -

- pde\_its
  : Parameter- PDEfield
    : Dish- periodic\_boundaries
      : Parameter- polarvec
        : Cell

### - r -

- relaxation
  : Parameter- rseed
    : Parameter

### - s -

- saturation
  : Parameter- secr\_rate
    : Parameter- sigma
      : PDE, Cell, CellularPotts- size\_init\_cells
        : Parameter- sizex
          : PDE, Parameter, CellularPotts- sizey
            : PDE, Parameter, CellularPotts- spins\_converted
              : CellularPotts- storage\_stride
                : Parameter- store
                  : Parameter- subfield
                    : Parameter- sum\_x
                      : Cell- sum\_xx
                        : Cell- sum\_xy
                          : Cell- sum\_y
                            : Cell- sum\_yy
                              : Cell

### - t -

- T
  : Parameter- target\_area
    : Parameter, Cell- target\_length
      : Parameter, Cell- tau
        : Cell- times\_divided
          : Cell

### - v -

- v
  : Cell- vecadherinknockout
    : Parameter

### - x -

- x
  : co, Point- x1
    : li- x2
      : li

### - y -

- y
  : co, Point- y1
    : li- y2
      : li

---

Generated on Tue Dec 12 16:32:41 2006 for Tissue Simulation Toolkit by

1.3.5
