# Supplementary material for: Contact-Inhibited Chemotaxis in De Novo and Sprouting Blood-Vessel Growth
Source: PLoS Comput Biol. 2008 Sep 19;4(9):e1000163. doi: 10.1371/journal.pcbi.1000163 (PMC2528254; doi:10.1371/journal.pcbi.1000163)
Supplement: Protocol S1 — Tissue Simulation Toolkit v0.1.3. The source code for the software used for the simulations presented in this paper is also available from http://sourceforge.net/projects/tst. Installation: Unpack and compile according to the instructions given in the INSTALL file The code is written in C++ using the cross-platform (Windows, Mac, or Unix/Linux) library Qt (available from www.trolltech.com). (332 KB ZIP) [file pcbi.1000163.s002.zip › TST0.1.3/html/globals.html]

Tissue Simulation Toolkit: File Member Index

Main Page | Namespace List | Class Hierarchy | Class List | File List | Namespace Members | Class Members | File Members

All | Functions | Variables | Typedefs | Defines

a | b | c | d | e | f | g | h | i | k | l | m | n | o | p | q | r | s | t | u | v | w | x | y | z

Here is a list of all file members with links to the files they belong to:

### - a -

- AskSeed()
  : random.h, random.cpp

### - b -

- bgetpar()
  : parse.h, parse.cpp- BLACK
    : sticky.h- BLUE
      : sticky.h- BOLTZMANN
        : sticky.h- bool\_str()
          : parse.h, parse.cpp

### - c -

- CanWeWriteP()
  : output.h- CFILE
    : x11graph.h- chainHull\_2D()
      : hull.h, hull.cpp- CheckFile()
        : output.h, output.cpp- Chext()
          : output.h, output.cpp- COMMUNICATION
            : sticky.h- conrec()
              : conrec.h, conrec.cpp- Coordinate
                : x11graph.h- copyprob
                  : ca.cpp- Crash()
                    : crash.h, crash.cpp

### - d -

- dgetparlist()
  : parse.h, parse.cpp- DIV
    : sticky.h- DMUT
      : sticky.h- DSQR
        : sqr.h

### - e -

- EMPTY
  : sticky.h- ENDOFSTATES
    : sticky.h- ENERGYOFFSET
      : sticky.h- errno
        : x11graph.cpp- error()
          : warning.h, warning.cpp- EXTERNAL\_OFF
            : dish.cpp

### - f -

- FAC
  : random.h- FALSE
    : sticky.h, output.h, misc.h- fgetpar()
      : parse.h, parse.cpp- FileExists()
        : misc.h, misc.cpp- FileExistsP()
          : output.h, output.cpp- FNAMESIZE
            : output.cpp

### - g -

- GetFileName()
  : misc.h, misc.cpp- GREEN
    : sticky.h- GRIDX
      : sticky.h- GRIDY
        : sticky.h

### - h -

- HandleSIGINT()
  : crash.h, crash.cpp- HandleSIGSEGV()
    : crash.h, crash.cpp- HASHCOLNUM
      : cell.cpp- HASHCOLPRIME
        : sticky.h- HOSTDEAD
          : sticky.h

### - i -

- igetpar()
  : parse.h, parse.cpp- INETSIZE
    : sticky.h- INIT
      : dish.h- INITIAL\_BUFSIZE
        : output.cpp- INP\_PROT
          : sticky.h- isLeft()
            : hull.cpp

### - k -

- KEYBUFSIZE
  : x11graph.cpp

### - l -

- LineType
  : x11graph.h

### - m -

- MakeDir()
  : output.h- max
    : conrec.cpp- MAXCELLS
      : sticky.h- MAXHIST
        : sticky.h- MAXNEIGH
          : sticky.h- MAXSEED
            : sticky.h- MAXTYPE
              : sticky.h- MBIG
                : random.h- MEDIUM
                  : sticky.h- MEMORYCHECK
                    : warning.h- MemoryWarning()
                      : crash.h, crash.cpp- MESS\_BUF\_SIZE
                        : output.h- min
                          : conrec.cpp- MOTION
                            : x11graph.h- MSEED
                              : random.h- MZ
                                : random.h

### - n -

- NETSIZE
  : sticky.h- NH\_TH
    : sticky.h- NHHIST
      : sticky.h- NiceMessage()
        : crash.h, crash.cpp- NOPVM
          : x11graph.cpp, crash.cpp- NULL\_BEAST
            : sticky.h

### - o -

- OK
  : sticky.h, misc.h- OK\_BEAST
    : sticky.h- OpenFileAndCheckExistance()
      : output.h, output.cpp- OpenGZippedWriteFile()
        : output.h- OpenReadFile()
          : output.h, output.cpp- OpenWriteFile()
            : output.h, output.cpp- operator<<()
              : parameter.h, parameter.cpp- OUTFILE
                : x11graph.h

### - p -

- par
  : x11graph.cpp, pde.cpp, parameter.cpp, info.cpp, dish.cpp, cell.h, cell.cpp, ca.cpp- ParsePar()
    : parse.h, parse.cpp- PCO
      : sticky.h- PLOTPERIODFREQUENCY
        : sticky.h- PMUT
          : sticky.h- PMUT2
            : sticky.h- POTPROT
              : sticky.h

### - q -

- Quiet
  : warning.cpp

### - r -

- RANDOM()
  : sticky.h, random.h, random.cpp- Randomize()
    : random.h, random.cpp- RandomNumber()
      : random.h, random.cpp- ReadDouble()
        : misc.h, misc.cpp- ReadLine()
          : output.h, output.cpp- ReadNumber()
            : misc.h, misc.cpp- RED
              : sticky.h- REMARK
                : sticky.h, misc.h- RESIZE
                  : x11graph.h

### - s -

- sat()
  : ca.cpp- sbool()
    : parameter.h, parameter.cpp- SearchToken()
      : parse.h, parse.cpp- Seed()
        : random.h, random.cpp- sgetpar()
          : parse.h, parse.cpp- SkipLine()
            : parse.h, parse.cpp- SkipToken()
              : parse.h, parse.cpp- SQR
                : sqr.h- StartSIGINTHandling()
                  : crash.h, crash.cpp- StartSIGSEGVHandling()
                    : crash.h, crash.cpp- SWAP
                      : x11graph.cpp

### - t -

- TESTCELLS
  : sticky.h- TIMESTEP
    : x11graph.h, qtgraph.h- TokenInLineP()
      : parse.h, parse.cpp- TRUE
        : sticky.h, output.h, misc.h

### - u -

- UNIDENTIFIED
  : warning.h

### - v -

- VERBOSE
  : x11graph.h

### - w -

- warning()
  : warning.h, warning.cpp- WHITE
    : sticky.h

### - x -

- XPM
  : ca.cpp- xsect
    : conrec.cpp

### - y -

- YesNoP()
  : output.h, output.cpp, misc.h, misc.cpp- ysect
    : conrec.cpp

### - z -

- ZYGFILE
  : ca.cpp- ZYGOTE
    : ca.cpp- ZYGXPM
      : ca.cpp

---

Generated on Tue Dec 12 16:32:41 2006 for Tissue Simulation Toolkit by

1.3.5
