# Supplementary material for: Contact-Inhibited Chemotaxis in De Novo and Sprouting Blood-Vessel Growth
Source: PLoS Comput Biol. 2008 Sep 19;4(9):e1000163. doi: 10.1371/journal.pcbi.1000163 (PMC2528254; doi:10.1371/journal.pcbi.1000163)
Supplement: Protocol S1 — Tissue Simulation Toolkit v0.1.3. The source code for the software used for the simulations presented in this paper is also available from http://sourceforge.net/projects/tst. Installation: Unpack and compile according to the instructions given in the INSTALL file The code is written in C++ using the cross-platform (Windows, Mac, or Unix/Linux) library Qt (available from www.trolltech.com). (332 KB ZIP) [file pcbi.1000163.s002.zip › TST0.1.3/html/globals_defs.html]

Tissue Simulation Toolkit: File Member Index

Main Page | Namespace List | Class Hierarchy | Class List | File List | Namespace Members | Class Members | File Members

All | Functions | Variables | Typedefs | Defines

b | c | d | e | f | g | h | i | k | m | n | o | p | r | s | t | u | v | w | x | y | z

### - b -

- BLACK
  : sticky.h- BLUE
    : sticky.h- BOLTZMANN
      : sticky.h

### - c -

- CFILE
  : x11graph.h- COMMUNICATION
    : sticky.h

### - d -

- DIV
  : sticky.h- DMUT
    : sticky.h- DSQR
      : sqr.h

### - e -

- EMPTY
  : sticky.h- ENDOFSTATES
    : sticky.h- ENERGYOFFSET
      : sticky.h- EXTERNAL\_OFF
        : dish.cpp

### - f -

- FAC
  : random.h- FALSE
    : sticky.h, output.h, misc.h- FNAMESIZE
      : output.cpp

### - g -

- GREEN
  : sticky.h- GRIDX
    : sticky.h- GRIDY
      : sticky.h

### - h -

- HASHCOLNUM
  : cell.cpp- HASHCOLPRIME
    : sticky.h- HOSTDEAD
      : sticky.h

### - i -

- INETSIZE
  : sticky.h- INIT
    : dish.h- INITIAL\_BUFSIZE
      : output.cpp- INP\_PROT
        : sticky.h

### - k -

- KEYBUFSIZE
  : x11graph.cpp

### - m -

- max
  : conrec.cpp- MAXCELLS
    : sticky.h- MAXHIST
      : sticky.h- MAXNEIGH
        : sticky.h- MAXSEED
          : sticky.h- MAXTYPE
            : sticky.h- MBIG
              : random.h- MEDIUM
                : sticky.h- MEMORYCHECK
                  : warning.h- MESS\_BUF\_SIZE
                    : output.h- min
                      : conrec.cpp- MOTION
                        : x11graph.h- MSEED
                          : random.h- MZ
                            : random.h

### - n -

- NETSIZE
  : sticky.h- NH\_TH
    : sticky.h- NHHIST
      : sticky.h- NOPVM
        : x11graph.cpp, crash.cpp- NULL\_BEAST
          : sticky.h

### - o -

- OK
  : sticky.h, misc.h- OK\_BEAST
    : sticky.h- OUTFILE
      : x11graph.h

### - p -

- PCO
  : sticky.h- PLOTPERIODFREQUENCY
    : sticky.h- PMUT
      : sticky.h- PMUT2
        : sticky.h- POTPROT
          : sticky.h

### - r -

- RED
  : sticky.h- REMARK
    : sticky.h, misc.h- RESIZE
      : x11graph.h

### - s -

- SQR
  : sqr.h- SWAP
    : x11graph.cpp

### - t -

- TESTCELLS
  : sticky.h- TIMESTEP
    : x11graph.h, qtgraph.h- TRUE
      : sticky.h, output.h, misc.h

### - u -

- UNIDENTIFIED
  : warning.h

### - v -

- VERBOSE
  : x11graph.h

### - w -

- WHITE
  : sticky.h

### - x -

- XPM
  : ca.cpp- xsect
    : conrec.cpp

### - y -

- ysect
  : conrec.cpp

### - z -

- ZYGFILE
  : ca.cpp- ZYGOTE
    : ca.cpp- ZYGXPM
      : ca.cpp

---

Generated on Tue Dec 12 16:32:41 2006 for Tissue Simulation Toolkit by

1.3.5
