# Supplementary material for: Contact-Inhibited Chemotaxis in De Novo and Sprouting Blood-Vessel Growth
Source: PLoS Comput Biol. 2008 Sep 19;4(9):e1000163. doi: 10.1371/journal.pcbi.1000163 (PMC2528254; doi:10.1371/journal.pcbi.1000163)
Supplement: Protocol S1 — Tissue Simulation Toolkit v0.1.3. The source code for the software used for the simulations presented in this paper is also available from http://sourceforge.net/projects/tst. Installation: Unpack and compile according to the instructions given in the INSTALL file The code is written in C++ using the cross-platform (Windows, Mac, or Unix/Linux) library Qt (available from www.trolltech.com). (332 KB ZIP) [file pcbi.1000163.s002.zip › TST0.1.3/html/globals_eval.html]

Tissue Simulation Toolkit: File Members - Enumeration values

Main Page | Namespace List | Class Hierarchy | Class List | File List | Namespace Members | Class Members | File Members

All | Functions | Variables | Typedefs | Enumeration values | Defines

- Auxilliary
  : dish.h- CellType
    : dish.h- Sigma
      : dish.h- State
        : dish.h

---

Generated on Mon Aug 28 17:27:08 2006 for Tissue Simulation Toolkit by 
 1.4.1
