# Supplementary material for: Contact-Inhibited Chemotaxis in De Novo and Sprouting Blood-Vessel Growth
Source: PLoS Comput Biol. 2008 Sep 19;4(9):e1000163. doi: 10.1371/journal.pcbi.1000163 (PMC2528254; doi:10.1371/journal.pcbi.1000163)
Supplement: Protocol S1 — Tissue Simulation Toolkit v0.1.3. The source code for the software used for the simulations presented in this paper is also available from http://sourceforge.net/projects/tst. Installation: Unpack and compile according to the instructions given in the INSTALL file The code is written in C++ using the cross-platform (Windows, Mac, or Unix/Linux) library Qt (available from www.trolltech.com). (332 KB ZIP) [file pcbi.1000163.s002.zip › TST0.1.3/html/globals_func.html]

Tissue Simulation Toolkit: File Member Index

Main Page | Namespace List | Class Hierarchy | Class List | File List | Namespace Members | Class Members | File Members

All | Functions | Variables | Typedefs | Defines

a | b | c | d | e | f | g | h | i | m | n | o | p | r | s | t | w | y

### - a -

- AskSeed()
  : random.h, random.cpp

### - b -

- bgetpar()
  : parse.h, parse.cpp- bool\_str()
    : parse.h, parse.cpp

### - c -

- CanWeWriteP()
  : output.h- chainHull\_2D()
    : hull.h, hull.cpp- CheckFile()
      : output.h, output.cpp- Chext()
        : output.h, output.cpp- conrec()
          : conrec.h, conrec.cpp- Crash()
            : crash.h, crash.cpp

### - d -

- dgetparlist()
  : parse.h, parse.cpp

### - e -

- error()
  : warning.h, warning.cpp

### - f -

- fgetpar()
  : parse.h, parse.cpp- FileExists()
    : misc.h, misc.cpp- FileExistsP()
      : output.h, output.cpp

### - g -

- GetFileName()
  : misc.h, misc.cpp

### - h -

- HandleSIGINT()
  : crash.h, crash.cpp- HandleSIGSEGV()
    : crash.h, crash.cpp

### - i -

- igetpar()
  : parse.h, parse.cpp- isLeft()
    : hull.cpp

### - m -

- MakeDir()
  : output.h- MemoryWarning()
    : crash.h, crash.cpp

### - n -

- NiceMessage()
  : crash.h, crash.cpp

### - o -

- OpenFileAndCheckExistance()
  : output.h, output.cpp- OpenGZippedWriteFile()
    : output.h- OpenReadFile()
      : output.h, output.cpp- OpenWriteFile()
        : output.h, output.cpp- operator<<()
          : parameter.h, parameter.cpp

### - p -

- ParsePar()
  : parse.h, parse.cpp

### - r -

- RANDOM()
  : sticky.h, random.h, random.cpp- Randomize()
    : random.h, random.cpp- RandomNumber()
      : random.h, random.cpp- ReadDouble()
        : misc.h, misc.cpp- ReadLine()
          : output.h, output.cpp- ReadNumber()
            : misc.h, misc.cpp

### - s -

- sat()
  : ca.cpp- sbool()
    : parameter.h, parameter.cpp- SearchToken()
      : parse.h, parse.cpp- Seed()
        : random.h, random.cpp- sgetpar()
          : parse.h, parse.cpp- SkipLine()
            : parse.h, parse.cpp- SkipToken()
              : parse.h, parse.cpp- StartSIGINTHandling()
                : crash.h, crash.cpp- StartSIGSEGVHandling()
                  : crash.h, crash.cpp

### - t -

- TokenInLineP()
  : parse.h, parse.cpp

### - w -

- warning()
  : warning.h, warning.cpp

### - y -

- YesNoP()
  : output.h, output.cpp, misc.h, misc.cpp

---

Generated on Tue Dec 12 16:32:41 2006 for Tissue Simulation Toolkit by

1.3.5
