# Supplementary material for: Contact-Inhibited Chemotaxis in De Novo and Sprouting Blood-Vessel Growth
Source: PLoS Comput Biol. 2008 Sep 19;4(9):e1000163. doi: 10.1371/journal.pcbi.1000163 (PMC2528254; doi:10.1371/journal.pcbi.1000163)
Supplement: Protocol S1 — Tissue Simulation Toolkit v0.1.3. The source code for the software used for the simulations presented in this paper is also available from http://sourceforge.net/projects/tst. Installation: Unpack and compile according to the instructions given in the INSTALL file The code is written in C++ using the cross-platform (Windows, Mac, or Unix/Linux) library Qt (available from www.trolltech.com). (332 KB ZIP) [file pcbi.1000163.s002.zip › TST0.1.3/html/graph_8h-source.html]

Tissue Simulation Toolkit: /home/romer/TST0.1.3/graph.h Source File

Main Page | Namespace List | Class Hierarchy | Class List | File List | Namespace Members | Class Members | File Members

# /home/romer/TST0.1.3/graph.h

Go to the documentation of this file.

```
00001 /* 
00002 
00003 Copyright 1996-2006 Roeland Merks
00004 
00005 This file is part of Tissue Simulation Toolkit.
00006 
00007 Tissue Simulation Toolkit is free software; you can redistribute
00008 it and/or modify it under the terms of the GNU General Public
00009 License as published by the Free Software Foundation; either
00010 version 2 of the License, or (at your option) any later version.
00011 
00012 Tissue Simulation Toolkit is distributed in the hope that it will
00013 be useful, but WITHOUT ANY WARRANTY; without even the implied
00014 warranty of MERCHANTABILITY or FITNESS FOR A PARTICULAR PURPOSE.
00015 See the GNU General Public License for more details.
00016 
00017 You should have received a copy of the GNU General Public License
00018 along with Tissue Simulation Toolkit; if not, write to the Free
00019 Software Foundation, Inc., 51 Franklin St, Fifth Floor, Boston, MA
00020 02110-1301 USA
00021 
00022 */
00023 
00031 #ifndef _GRAPH_H_
00032 #define _GRAPH_H_
00033 #include <iostream>
00034 // Base class for Graphics interface. No implementation
00035 
00036 class Graphics {
00037 
00038  public:
00039   //  Graphics(int xfield, int yfield, const char *movie_file=0);
00040   virtual ~Graphics(void) {};
00041   
00043   virtual void BeginScene(void) {
00044   };
00046   virtual void EndScene(void) {
00047   };
00053   virtual void Point( int color, int x, int y)=0;
00054   
00061   virtual void Line(int x1, int y1,int x2,int y2,int colour )=0;
00062   
00070   virtual int GetXYCoo(int *X,int *Y)=0;// {return 0;}
00071   
00073   virtual int XField(void) const {return 0;}
00074   
00076   virtual int YField(void) const {return 0;}
00077   
00087   virtual void Write(char *fname, int quality=-1)=0;
00088 
00094   virtual void TimeStep(void) {};
00095   
00105   virtual void Field(const int **f, int mag=1) {
00106     throw "Graphics::Field not implemented. Try X11 graphics.\n";
00107   }
00108   
00109 };
00110 
00111 
00112 #endif
00113
```

---

Generated on Tue Dec 12 16:32:40 2006 for Tissue Simulation Toolkit by

1.3.5 
