# Supplementary material for: Contact-Inhibited Chemotaxis in De Novo and Sprouting Blood-Vessel Growth
Source: PLoS Comput Biol. 2008 Sep 19;4(9):e1000163. doi: 10.1371/journal.pcbi.1000163 (PMC2528254; doi:10.1371/journal.pcbi.1000163)
Supplement: Protocol S1 — Tissue Simulation Toolkit v0.1.3. The source code for the software used for the simulations presented in this paper is also available from http://sourceforge.net/projects/tst. Installation: Unpack and compile according to the instructions given in the INSTALL file The code is written in C++ using the cross-platform (Windows, Mac, or Unix/Linux) library Qt (available from www.trolltech.com). (332 KB ZIP) [file pcbi.1000163.s002.zip › TST0.1.3/html/graph_8h.html]

Tissue Simulation Toolkit: graph.h File Reference

Main Page | Namespace List | Class Hierarchy | Class List | File List | Namespace Members | Class Members | File Members

# /home/romer/TST0.1.3/graph.h File Reference

`#include <iostream>`  

Go to the source code of this file.

|  |
| --- |
|  |
| Classes | |
| class | Graphics |
|  | API for Graphics windows.  More... |

---

Generated on Tue Dec 12 16:32:41 2006 for Tissue Simulation Toolkit by

1.3.5
