# Supplementary material for: Contact-Inhibited Chemotaxis in De Novo and Sprouting Blood-Vessel Growth
Source: PLoS Comput Biol. 2008 Sep 19;4(9):e1000163. doi: 10.1371/journal.pcbi.1000163 (PMC2528254; doi:10.1371/journal.pcbi.1000163)
Supplement: Protocol S1 — Tissue Simulation Toolkit v0.1.3. The source code for the software used for the simulations presented in this paper is also available from http://sourceforge.net/projects/tst. Installation: Unpack and compile according to the instructions given in the INSTALL file The code is written in C++ using the cross-platform (Windows, Mac, or Unix/Linux) library Qt (available from www.trolltech.com). (332 KB ZIP) [file pcbi.1000163.s002.zip › TST0.1.3/html/hull_8h-source.html]

Tissue Simulation Toolkit: /home/romer/TST0.1.3/hull.h Source File

Main Page | Namespace List | Class Hierarchy | Class List | File List | Namespace Members | Class Members | File Members

# /home/romer/TST0.1.3/hull.h

Go to the documentation of this file.

```
00001 // Class point needed by 2D convex hull code
00002 class Point {
00003 
00004 public:
00005   Point(float xx, float yy) {
00006     x=xx;
00007     y=yy;
00008   }
00009   Point(void) {
00010     x=0; y=0;
00011   }
00012   float x,y;
00013 
00014 };
00015 
00016 int chainHull_2D( Point* P, int n, Point* H );
00017
```

---

Generated on Tue Dec 12 16:32:40 2006 for Tissue Simulation Toolkit by

1.3.5 
