# Supplementary material for: Contact-Inhibited Chemotaxis in De Novo and Sprouting Blood-Vessel Growth
Source: PLoS Comput Biol. 2008 Sep 19;4(9):e1000163. doi: 10.1371/journal.pcbi.1000163 (PMC2528254; doi:10.1371/journal.pcbi.1000163)
Supplement: Protocol S1 — Tissue Simulation Toolkit v0.1.3. The source code for the software used for the simulations presented in this paper is also available from http://sourceforge.net/projects/tst. Installation: Unpack and compile according to the instructions given in the INSTALL file The code is written in C++ using the cross-platform (Windows, Mac, or Unix/Linux) library Qt (available from www.trolltech.com). (332 KB ZIP) [file pcbi.1000163.s002.zip › TST0.1.3/html/index.html]

Tissue Simulation Toolkit: Tissue Simulation Toolkit, v0.1.3

Main Page | Namespace List | Class Hierarchy | Class List | File List | Namespace Members | Class Members | File Members

# Tissue Simulation Toolkit, v0.1.3

### 0.1.3

Welcome to the Tissue Simulation Toolkit, a library for two-dimensional simulations of Glazier and Graner's Cellular Potts model (Glazier and Graner, 1993).

The TST aims to provide a simple set of computational tools to get you started with the Cellular Potts Model (CPM), also called Glazier-Graner-Hogeweg (GGH) model.

The current version of the TST includes example programs for the following published simulations:

- Differential adhesion driven cell sorting (Glazier and Graner, 1993)
- Cell elongation dependent vasculogenesis (blood vessel growth) (Merks et al., 2006)
- Vasulogenesis and angiogenic sprouting driven by contact-inhibited chemotaxis (Merks and Glazier, 2006; Merks et al, PLoS Comp. Biol.)

The TST provides many recent extensions to the CPM, including

- Infinite number of PDE layers (forward Euler)
- Interaction of CPM cells and PDE (secretion, absorption)
- Chemotaxis
- Length and connectivity constraints

and visualization of:

- Cells, according to cell type or anything you wish
- Chemical fields, using color ramps and contour lines (level sets)

## Installation

The TST has been compiled on Windows XP (minGW), and Linux and MacOSX systems (GNU C++). The following libraries are required:

Qt (version 4.3) libpng (url) and libz (url)

For Unix, we also have an X11-only version, although compilation is easiest with the qmake tool shipped with Qt (see http://www.trolltech.com)

With Qt, simply type:

qmake make

If you want to use a different "main" program (examples are given: vessel.cpp and sorting.cpp), just change "TARGET" in the file "CellularPotts2.pro".

## Programs

A small Cellular Potts tutorial making use of the example programs sorting.cpp and vessel.cpp is provided by the document "exercises.pdf" included in the source package.

## Documentation

An automatically generated class documentation is included in html/index.html.

## References

- Glazier, J. A. and Graner, F. 1993. Simulation of the differential adhesion driven rearrangement of biological cells. Phys. Rev. E 47, 2128-2145.

- Merks, R. M. H., Brodsky, S. V., Goligorsky, M. S., Newman, S. A. and Glazier, J. A., 2006. Cell elongation is key to in silico replication of in vitro vasculogenesis and subsequent remodelling. Dev. Biol. 289, 44-54.

- Merks, R. M. H. and Glazier, J. A., 2005. A cell-centered approach to developmental biology. Phys. A. 352, 113-130.

- Merks, R. M. H. and Glazier, J. A., 2006. Dynamic mechanisms of blood vessel growth. Nonlinearity 19, C1-C10.

- Merks, R. M. H., Perryn, E. D. and Glazier, J. A. Contact-inhibited chemotactic motility in de novo and sprouting blood vessel growth. PLoS Computational Biology

---

Generated on Tue Dec 12 16:32:40 2006 for Tissue Simulation Toolkit by

1.3.5
