# Supplementary material for: Contact-Inhibited Chemotaxis in De Novo and Sprouting Blood-Vessel Growth
Source: PLoS Comput Biol. 2008 Sep 19;4(9):e1000163. doi: 10.1371/journal.pcbi.1000163 (PMC2528254; doi:10.1371/journal.pcbi.1000163)
Supplement: Protocol S1 — Tissue Simulation Toolkit v0.1.3. The source code for the software used for the simulations presented in this paper is also available from http://sourceforge.net/projects/tst. Installation: Unpack and compile according to the instructions given in the INSTALL file The code is written in C++ using the cross-platform (Windows, Mac, or Unix/Linux) library Qt (available from www.trolltech.com). (332 KB ZIP) [file pcbi.1000163.s002.zip › TST0.1.3/html/misc_8h.html]

Tissue Simulation Toolkit: misc.h File Reference

Main Page | Namespace List | Class Hierarchy | Class List | File List | Namespace Members | Class Members | File Members

# /home/romer/TST0.1.3/misc.h File Reference

Go to the source code of this file.

|  |
| --- |
|  |
| Defines | |
| #define | FALSE   0 |
| #define | TRUE   1 |
| #define | OK   1 |
| #define | REMARK   56 |
| Functions | |
| int | ReadNumber (FILE \*file, int \*number) |
| int | YesNoP (const char \*message) |
| char \* | GetFileName (const char \*message, const char \*ftype) |
| int | FileExists (FILE \*\*fp, const char \*fname, const char \*ftype) |
| int | ReadDouble (FILE \*file, double \*number) |

---

## Define Documentation

|  |  |
| --- | --- |
| |  | | --- | | #define FALSE   0 | |

|  |  |
| --- | --- |
|  |  |

|  |  |
| --- | --- |
| |  | | --- | | #define OK   1 | |

|  |  |
| --- | --- |
|  |  |

|  |  |
| --- | --- |
| |  | | --- | | #define REMARK   56 | |

|  |  |
| --- | --- |
|  |  |

|  |  |
| --- | --- |
| |  | | --- | | #define TRUE   1 | |

|  |  |
| --- | --- |
|  |  |

---

## Function Documentation

|  |  |  |  |  |  |  |  |  |  |  |  |  |  |  |  |  |
| --- | --- | --- | --- | --- | --- | --- | --- | --- | --- | --- | --- | --- | --- | --- | --- | --- |
| |  |  |  |  | | --- | --- | --- | --- | | int FileExists | ( | FILE \*\* | *fp*, | |  |  | const char \* | *fname*, | |  |  | const char \* | *ftype* | |  | ) |  | | |

|  |  |
| --- | --- |
|  |  |

|  |  |  |  |  |  |  |  |  |  |  |  |  |
| --- | --- | --- | --- | --- | --- | --- | --- | --- | --- | --- | --- | --- |
| |  |  |  |  | | --- | --- | --- | --- | | char\* GetFileName | ( | const char \* | *message*, | |  |  | const char \* | *ftype* | |  | ) |  | | |

|  |  |
| --- | --- |
|  |  |

|  |  |  |  |  |  |  |  |  |  |  |  |  |
| --- | --- | --- | --- | --- | --- | --- | --- | --- | --- | --- | --- | --- |
| |  |  |  |  | | --- | --- | --- | --- | | int ReadDouble | ( | FILE \* | *file*, | |  |  | double \* | *number* | |  | ) |  | | |

|  |  |
| --- | --- |
|  |  |

|  |  |  |  |  |  |  |  |  |  |  |  |  |
| --- | --- | --- | --- | --- | --- | --- | --- | --- | --- | --- | --- | --- |
| |  |  |  |  | | --- | --- | --- | --- | | int ReadNumber | ( | FILE \* | *file*, | |  |  | int \* | *number* | |  | ) |  | | |

|  |  |
| --- | --- |
|  | PUBLIC \* |

|  |  |  |  |  |  |  |
| --- | --- | --- | --- | --- | --- | --- |
| |  |  |  |  |  |  | | --- | --- | --- | --- | --- | --- | | int YesNoP | ( | const char \* | *message* | ) |  | |

|  |  |
| --- | --- |
|  |  |

---

Generated on Tue Dec 12 16:32:41 2006 for Tissue Simulation Toolkit by

1.3.5
