# Supplementary material for: Contact-Inhibited Chemotaxis in De Novo and Sprouting Blood-Vessel Growth
Source: PLoS Comput Biol. 2008 Sep 19;4(9):e1000163. doi: 10.1371/journal.pcbi.1000163 (PMC2528254; doi:10.1371/journal.pcbi.1000163)
Supplement: Protocol S1 — Tissue Simulation Toolkit v0.1.3. The source code for the software used for the simulations presented in this paper is also available from http://sourceforge.net/projects/tst. Installation: Unpack and compile according to the instructions given in the INSTALL file The code is written in C++ using the cross-platform (Windows, Mac, or Unix/Linux) library Qt (available from www.trolltech.com). (332 KB ZIP) [file pcbi.1000163.s002.zip › TST0.1.3/html/output_8cpp.html]

Tissue Simulation Toolkit: output.cpp File Reference

Main Page | Namespace List | Class Hierarchy | Class List | File List | Namespace Members | Class Members | File Members

# /home/romer/TST0.1.3/output.cpp File Reference

`#include <stdio.h>`  
`#include <string.h>`  
`#include <stdlib.h>`  
`#include <errno.h>`  
`#include <sys/types.h>`  
`#include <sys/stat.h>`  
`#include "warning.h"`  
`#include "parameter.h"`  
`#include "output.h"`  

|  |
| --- |
|  |
| Defines | |
| #define | FNAMESIZE   100 |
| #define | INITIAL\_BUFSIZE   100 |
| Functions | |
| int | OpenFileAndCheckExistance (FILE \*\*fp, const char \*fname, char \*ftype) |
| int | FileExistsP (const char \*fname) |
| int | YesNoP (const char \*message) |
| FILE \* | OpenWriteFile (const char \*filename) |
| FILE \* | OpenReadFile (const char \*filename) |
| char \* | ReadLine (FILE \*fp) |
| void | CheckFile (FILE \*fp) |
| char \* | Chext (char \*filename) |

---

## Define Documentation

|  |  |
| --- | --- |
| |  | | --- | | #define FNAMESIZE   100 | |

|  |  |
| --- | --- |
|  |  |

|  |  |
| --- | --- |
| |  | | --- | | #define INITIAL\_BUFSIZE   100 | |

|  |  |
| --- | --- |
|  |  |

---

## Function Documentation

|  |  |  |  |  |  |  |
| --- | --- | --- | --- | --- | --- | --- |
| |  |  |  |  |  |  | | --- | --- | --- | --- | --- | --- | | void CheckFile | ( | FILE \* | *fp* | ) |  | |

|  |  |
| --- | --- |
|  |  |

|  |  |  |  |  |  |  |
| --- | --- | --- | --- | --- | --- | --- |
| |  |  |  |  |  |  | | --- | --- | --- | --- | --- | --- | | char\* Chext | ( | char \* | *filename* | ) |  | |

|  |  |
| --- | --- |
|  |  |

|  |  |  |  |  |  |  |
| --- | --- | --- | --- | --- | --- | --- |
| |  |  |  |  |  |  | | --- | --- | --- | --- | --- | --- | | int FileExistsP | ( | const char \* | *fname* | ) |  | |

|  |  |
| --- | --- |
|  |  |

|  |  |  |  |  |  |  |  |  |  |  |  |  |  |  |  |  |
| --- | --- | --- | --- | --- | --- | --- | --- | --- | --- | --- | --- | --- | --- | --- | --- | --- |
| |  |  |  |  | | --- | --- | --- | --- | | int OpenFileAndCheckExistance | ( | FILE \*\* | *fp*, | |  |  | const char \* | *fname*, | |  |  | char \* | *ftype* | |  | ) |  | | |

|  |  |
| --- | --- |
|  |  |

|  |  |  |  |  |  |  |
| --- | --- | --- | --- | --- | --- | --- |
| |  |  |  |  |  |  | | --- | --- | --- | --- | --- | --- | | FILE\* OpenReadFile | ( | const char \* | *filename* | ) |  | |

|  |  |
| --- | --- |
|  |  |

|  |  |  |  |  |  |  |
| --- | --- | --- | --- | --- | --- | --- |
| |  |  |  |  |  |  | | --- | --- | --- | --- | --- | --- | | FILE\* OpenWriteFile | ( | const char \* | *filename* | ) |  | |

|  |  |
| --- | --- |
|  |  |

|  |  |  |  |  |  |  |
| --- | --- | --- | --- | --- | --- | --- |
| |  |  |  |  |  |  | | --- | --- | --- | --- | --- | --- | | char\* ReadLine | ( | FILE \* | *fp* | ) |  | |

|  |  |
| --- | --- |
|  |  |

|  |  |  |  |  |  |  |
| --- | --- | --- | --- | --- | --- | --- |
| |  |  |  |  |  |  | | --- | --- | --- | --- | --- | --- | | int YesNoP | ( | const char \* | *message* | ) |  | |

|  |  |
| --- | --- |
|  |  |

---

Generated on Tue Dec 12 16:32:41 2006 for Tissue Simulation Toolkit by

1.3.5
