# Supplementary material for: Contact-Inhibited Chemotaxis in De Novo and Sprouting Blood-Vessel Growth
Source: PLoS Comput Biol. 2008 Sep 19;4(9):e1000163. doi: 10.1371/journal.pcbi.1000163 (PMC2528254; doi:10.1371/journal.pcbi.1000163)
Supplement: Protocol S1 — Tissue Simulation Toolkit v0.1.3. The source code for the software used for the simulations presented in this paper is also available from http://sourceforge.net/projects/tst. Installation: Unpack and compile according to the instructions given in the INSTALL file The code is written in C++ using the cross-platform (Windows, Mac, or Unix/Linux) library Qt (available from www.trolltech.com). (332 KB ZIP) [file pcbi.1000163.s002.zip › TST0.1.3/html/parameter_8cpp.html]

Tissue Simulation Toolkit: parameter.cpp File Reference

Main Page | Namespace List | Class Hierarchy | Class List | File List | Namespace Members | Class Members | File Members

# /home/romer/TST0.1.3/parameter.cpp File Reference

`#include "parameter.h"`  
`#include <cstdio>`  
`#include <cstring>`  
`#include <cstdlib>`  
`#include <cerrno>`  
`#include <iostream>`  
`#include "output.h"`  
`#include "parse.h"`  

|  |
| --- |
|  |
| Functions | |
| const char \* | sbool (const bool &p) |
| ostream & | operator<< (ostream &os, Parameter &p) |
| Variables | |
| Parameter | par |

---

## Function Documentation

|  |  |  |  |  |  |  |  |  |  |  |  |  |
| --- | --- | --- | --- | --- | --- | --- | --- | --- | --- | --- | --- | --- |
| |  |  |  |  | | --- | --- | --- | --- | | ostream& operator<< | ( | ostream & | *os*, | |  |  | Parameter & | *p* | |  | ) |  | | |

|  |  |
| --- | --- |
|  |  |

|  |  |  |  |  |  |  |
| --- | --- | --- | --- | --- | --- | --- |
| |  |  |  |  |  |  | | --- | --- | --- | --- | --- | --- | | const char\* sbool | ( | const bool & | *p* | ) |  | |

|  |  |
| --- | --- |
|  |  |

---

## Variable Documentation

|  |  |
| --- | --- |
| |  | | --- | | Parameter par | |

|  |  |
| --- | --- |
|  |  |

---

Generated on Tue Dec 12 16:32:41 2006 for Tissue Simulation Toolkit by

1.3.5
