# Supplementary material for: Contact-Inhibited Chemotaxis in De Novo and Sprouting Blood-Vessel Growth
Source: PLoS Comput Biol. 2008 Sep 19;4(9):e1000163. doi: 10.1371/journal.pcbi.1000163 (PMC2528254; doi:10.1371/journal.pcbi.1000163)
Supplement: Protocol S1 — Tissue Simulation Toolkit v0.1.3. The source code for the software used for the simulations presented in this paper is also available from http://sourceforge.net/projects/tst. Installation: Unpack and compile according to the instructions given in the INSTALL file The code is written in C++ using the cross-platform (Windows, Mac, or Unix/Linux) library Qt (available from www.trolltech.com). (332 KB ZIP) [file pcbi.1000163.s002.zip › TST0.1.3/html/parameter_8h-source.html]

Tissue Simulation Toolkit: /home/romer/TST0.1.3/parameter.h Source File

Main Page | Namespace List | Class Hierarchy | Class List | File List | Namespace Members | Class Members | File Members

# /home/romer/TST0.1.3/parameter.h

Go to the documentation of this file.

```
00001 /* 
00002 
00003 Copyright 1996-2006 Roeland Merks
00004 
00005 This file is part of Tissue Simulation Toolkit.
00006 
00007 Tissue Simulation Toolkit is free software; you can redistribute
00008 it and/or modify it under the terms of the GNU General Public
00009 License as published by the Free Software Foundation; either
00010 version 2 of the License, or (at your option) any later version.
00011 
00012 Tissue Simulation Toolkit is distributed in the hope that it will
00013 be useful, but WITHOUT ANY WARRANTY; without even the implied
00014 warranty of MERCHANTABILITY or FITNESS FOR A PARTICULAR PURPOSE.
00015 See the GNU General Public License for more details.
00016 
00017 You should have received a copy of the GNU General Public License
00018 along with Tissue Simulation Toolkit; if not, write to the Free
00019 Software Foundation, Inc., 51 Franklin St, Fifth Floor, Boston, MA
00020 02110-1301 USA
00021 
00022 */
00023 #ifndef _PARAMETER_H_
00024 #define _PARAMETER_H_
00025 
00026 #include <iostream>
00027 using namespace std;
00028 class Parameter {
00029   
00030  public: 
00031   Parameter();
00032   ~Parameter();
00033   void CleanUp(void);
00034   void Read(const char *filename);
00035   void Write(ostream &os) const;
00036   double T;
00037   int target_area;
00038   int target_length;
00039   double lambda;
00040   double lambda2;
00041   char * Jtable;
00042   int conn_diss;
00043   bool vecadherinknockout;
00044   bool extensiononly;
00045   int chemotaxis;
00046   int border_energy;
00047   int neighbours;
00048   bool periodic_boundaries;
00049   int n_chem;
00050   double * diff_coeff;
00051   double * decay_rate;
00052   double * secr_rate;
00053   double saturation;
00054   double dt;
00055   double dx;
00056   int pde_its;
00057   int n_init_cells;
00058   int size_init_cells;
00059   int sizex;
00060   int sizey;
00061   int divisions;
00062   int mcs;
00063   int rseed;
00064   double subfield;
00065   int relaxation;
00066   int storage_stride;
00067   bool graphics;
00068   bool store;
00069   char * datadir;
00070  private:
00071 };
00072 
00073 ostream &operator<<(ostream &os, Parameter &p);
00074 const char *sbool(const bool &p);
00075 
00076 
00077 #endif
```

---

Generated on Tue Dec 12 16:32:40 2006 for Tissue Simulation Toolkit by

1.3.5 
