# Supplementary material for: Contact-Inhibited Chemotaxis in De Novo and Sprouting Blood-Vessel Growth
Source: PLoS Comput Biol. 2008 Sep 19;4(9):e1000163. doi: 10.1371/journal.pcbi.1000163 (PMC2528254; doi:10.1371/journal.pcbi.1000163)
Supplement: Protocol S1 — Tissue Simulation Toolkit v0.1.3. The source code for the software used for the simulations presented in this paper is also available from http://sourceforge.net/projects/tst. Installation: Unpack and compile according to the instructions given in the INSTALL file The code is written in C++ using the cross-platform (Windows, Mac, or Unix/Linux) library Qt (available from www.trolltech.com). (332 KB ZIP) [file pcbi.1000163.s002.zip › TST0.1.3/html/parse_8h.html]

Tissue Simulation Toolkit: parse.h File Reference

Main Page | Namespace List | Class Hierarchy | Class List | File List | Namespace Members | Class Members | File Members

# /home/romer/TST0.1.3/parse.h File Reference

Go to the source code of this file.

|  |
| --- |
|  |
| Functions | |
| char \* | ParsePar (FILE \*fp, char \*parameter, bool wrapflag) |
| int | igetpar (FILE \*fp, char \*parameter, bool wrapflag) |
| int | igetpar (FILE \*fp, char \*parameter, int default\_val, bool wrapflag) |
| float | fgetpar (FILE \*fp, char \*parameter, bool wrapflag) |
| float | fgetpar (FILE \*fp, char \*parameter, double default\_val, bool wrapflag) |
| double \* | dgetparlist (FILE \*fp, char \*parameter, int n, bool wrapflag) |
| char \* | sgetpar (FILE \*fp, char \*parameter, bool wrapflag) |
| char \* | sgetpar (FILE \*fp, char \*parameter, const char \*default\_val, bool wrapflag) |
| bool | bgetpar (FILE \*fp, char \*parameter, bool wrapflag) |
| bool | bgetpar (FILE \*fp, char \*parameter, int default\_val, bool wrapflag) |
| char \* | SearchToken (FILE \*fp, char \*token, bool wrapflag) |
| int | TokenInLineP (char \*line, char \*token) |
| void | SkipToken (FILE \*fp, char \*token, bool wrapflag) |
| void | SkipLine (FILE \*fp) |
| char \* | bool\_str (bool bool\_var) |

---

## Function Documentation

|  |  |  |  |  |  |  |  |  |  |  |  |  |  |  |  |  |  |  |  |  |
| --- | --- | --- | --- | --- | --- | --- | --- | --- | --- | --- | --- | --- | --- | --- | --- | --- | --- | --- | --- | --- |
| |  |  |  |  | | --- | --- | --- | --- | | bool bgetpar | ( | FILE \* | *fp*, | |  |  | char \* | *parameter*, | |  |  | int | *default\_val*, | |  |  | bool | *wrapflag* | |  | ) |  | | |

|  |  |
| --- | --- |
|  |  |

|  |  |  |  |  |  |  |  |  |  |  |  |  |  |  |  |  |
| --- | --- | --- | --- | --- | --- | --- | --- | --- | --- | --- | --- | --- | --- | --- | --- | --- |
| |  |  |  |  | | --- | --- | --- | --- | | bool bgetpar | ( | FILE \* | *fp*, | |  |  | char \* | *parameter*, | |  |  | bool | *wrapflag* | |  | ) |  | | |

|  |  |
| --- | --- |
|  |  |

|  |  |  |  |  |  |  |
| --- | --- | --- | --- | --- | --- | --- |
| |  |  |  |  |  |  | | --- | --- | --- | --- | --- | --- | | char\* bool\_str | ( | bool | *bool\_var* | ) |  | |

|  |  |
| --- | --- |
|  |  |

|  |  |  |  |  |  |  |  |  |  |  |  |  |  |  |  |  |  |  |  |  |
| --- | --- | --- | --- | --- | --- | --- | --- | --- | --- | --- | --- | --- | --- | --- | --- | --- | --- | --- | --- | --- |
| |  |  |  |  | | --- | --- | --- | --- | | double\* dgetparlist | ( | FILE \* | *fp*, | |  |  | char \* | *parameter*, | |  |  | int | *n*, | |  |  | bool | *wrapflag* | |  | ) |  | | |

|  |  |
| --- | --- |
|  |  |

|  |  |  |  |  |  |  |  |  |  |  |  |  |  |  |  |  |  |  |  |  |
| --- | --- | --- | --- | --- | --- | --- | --- | --- | --- | --- | --- | --- | --- | --- | --- | --- | --- | --- | --- | --- |
| |  |  |  |  | | --- | --- | --- | --- | | float fgetpar | ( | FILE \* | *fp*, | |  |  | char \* | *parameter*, | |  |  | double | *default\_val*, | |  |  | bool | *wrapflag* | |  | ) |  | | |

|  |  |
| --- | --- |
|  |  |

|  |  |  |  |  |  |  |  |  |  |  |  |  |  |  |  |  |
| --- | --- | --- | --- | --- | --- | --- | --- | --- | --- | --- | --- | --- | --- | --- | --- | --- |
| |  |  |  |  | | --- | --- | --- | --- | | float fgetpar | ( | FILE \* | *fp*, | |  |  | char \* | *parameter*, | |  |  | bool | *wrapflag* | |  | ) |  | | |

|  |  |
| --- | --- |
|  |  |

|  |  |  |  |  |  |  |  |  |  |  |  |  |  |  |  |  |  |  |  |  |
| --- | --- | --- | --- | --- | --- | --- | --- | --- | --- | --- | --- | --- | --- | --- | --- | --- | --- | --- | --- | --- |
| |  |  |  |  | | --- | --- | --- | --- | | int igetpar | ( | FILE \* | *fp*, | |  |  | char \* | *parameter*, | |  |  | int | *default\_val*, | |  |  | bool | *wrapflag* | |  | ) |  | | |

|  |  |
| --- | --- |
|  |  |

|  |  |  |  |  |  |  |  |  |  |  |  |  |  |  |  |  |
| --- | --- | --- | --- | --- | --- | --- | --- | --- | --- | --- | --- | --- | --- | --- | --- | --- |
| |  |  |  |  | | --- | --- | --- | --- | | int igetpar | ( | FILE \* | *fp*, | |  |  | char \* | *parameter*, | |  |  | bool | *wrapflag* | |  | ) |  | | |

|  |  |
| --- | --- |
|  |  |

|  |  |  |  |  |  |  |  |  |  |  |  |  |  |  |  |  |
| --- | --- | --- | --- | --- | --- | --- | --- | --- | --- | --- | --- | --- | --- | --- | --- | --- |
| |  |  |  |  | | --- | --- | --- | --- | | char\* ParsePar | ( | FILE \* | *fp*, | |  |  | char \* | *parameter*, | |  |  | bool | *wrapflag* | |  | ) |  | | |

|  |  |
| --- | --- |
|  |  |

|  |  |  |  |  |  |  |  |  |  |  |  |  |  |  |  |  |
| --- | --- | --- | --- | --- | --- | --- | --- | --- | --- | --- | --- | --- | --- | --- | --- | --- |
| |  |  |  |  | | --- | --- | --- | --- | | char\* SearchToken | ( | FILE \* | *fp*, | |  |  | char \* | *token*, | |  |  | bool | *wrapflag* | |  | ) |  | | |

|  |  |
| --- | --- |
|  |  |

|  |  |  |  |  |  |  |  |  |  |  |  |  |  |  |  |  |  |  |  |  |
| --- | --- | --- | --- | --- | --- | --- | --- | --- | --- | --- | --- | --- | --- | --- | --- | --- | --- | --- | --- | --- |
| |  |  |  |  | | --- | --- | --- | --- | | char\* sgetpar | ( | FILE \* | *fp*, | |  |  | char \* | *parameter*, | |  |  | const char \* | *default\_val*, | |  |  | bool | *wrapflag* | |  | ) |  | | |

|  |  |
| --- | --- |
|  |  |

|  |  |  |  |  |  |  |  |  |  |  |  |  |  |  |  |  |
| --- | --- | --- | --- | --- | --- | --- | --- | --- | --- | --- | --- | --- | --- | --- | --- | --- |
| |  |  |  |  | | --- | --- | --- | --- | | char\* sgetpar | ( | FILE \* | *fp*, | |  |  | char \* | *parameter*, | |  |  | bool | *wrapflag* | |  | ) |  | | |

|  |  |
| --- | --- |
|  |  |

|  |  |  |  |  |  |  |
| --- | --- | --- | --- | --- | --- | --- |
| |  |  |  |  |  |  | | --- | --- | --- | --- | --- | --- | | void SkipLine | ( | FILE \* | *fp* | ) |  | |

|  |  |
| --- | --- |
|  |  |

|  |  |  |  |  |  |  |  |  |  |  |  |  |  |  |  |  |
| --- | --- | --- | --- | --- | --- | --- | --- | --- | --- | --- | --- | --- | --- | --- | --- | --- |
| |  |  |  |  | | --- | --- | --- | --- | | void SkipToken | ( | FILE \* | *fp*, | |  |  | char \* | *token*, | |  |  | bool | *wrapflag* | |  | ) |  | | |

|  |  |
| --- | --- |
|  |  |

|  |  |  |  |  |  |  |  |  |  |  |  |  |
| --- | --- | --- | --- | --- | --- | --- | --- | --- | --- | --- | --- | --- |
| |  |  |  |  | | --- | --- | --- | --- | | int TokenInLineP | ( | char \* | *line*, | |  |  | char \* | *token* | |  | ) |  | | |

|  |  |
| --- | --- |
|  |  |

---

Generated on Tue Dec 12 16:32:41 2006 for Tissue Simulation Toolkit by

1.3.5
