# Supplementary material for: Contact-Inhibited Chemotaxis in De Novo and Sprouting Blood-Vessel Growth
Source: PLoS Comput Biol. 2008 Sep 19;4(9):e1000163. doi: 10.1371/journal.pcbi.1000163 (PMC2528254; doi:10.1371/journal.pcbi.1000163)
Supplement: Protocol S1 — Tissue Simulation Toolkit v0.1.3. The source code for the software used for the simulations presented in this paper is also available from http://sourceforge.net/projects/tst. Installation: Unpack and compile according to the instructions given in the INSTALL file The code is written in C++ using the cross-platform (Windows, Mac, or Unix/Linux) library Qt (available from www.trolltech.com). (332 KB ZIP) [file pcbi.1000163.s002.zip › TST0.1.3/html/pde_8h-source.html]

Tissue Simulation Toolkit: /home/romer/TST0.1.3/pde.h Source File

Main Page | Namespace List | Class Hierarchy | Class List | File List | Namespace Members | Class Members | File Members

# /home/romer/TST0.1.3/pde.h

Go to the documentation of this file.

```
00001 /* 
00002 
00003 Copyright 1996-2006 Roeland Merks
00004 
00005 This file is part of Tissue Simulation Toolkit.
00006 
00007 Tissue Simulation Toolkit is free software; you can redistribute
00008 it and/or modify it under the terms of the GNU General Public
00009 License as published by the Free Software Foundation; either
00010 version 2 of the License, or (at your option) any later version.
00011 
00012 Tissue Simulation Toolkit is distributed in the hope that it will
00013 be useful, but WITHOUT ANY WARRANTY; without even the implied
00014 warranty of MERCHANTABILITY or FITNESS FOR A PARTICULAR PURPOSE.
00015 See the GNU General Public License for more details.
00016 
00017 You should have received a copy of the GNU General Public License
00018 along with Tissue Simulation Toolkit; if not, write to the Free
00019 Software Foundation, Inc., 51 Franklin St, Fifth Floor, Boston, MA
00020 02110-1301 USA
00021 
00022 */
00023 
00024 #ifndef _PDE_HH_
00025 #define _PDE_HH_
00026 #include <stdio.h>
00027 #include <float.h>
00028 #include "graph.h"
00029 
00030 class CellularPotts;
00031 class PDE {
00032 
00033   friend class Info;
00034 
00035  public:
00036 
00044   PDE(const int layers, const int sizex, 
00045       const int sizey);
00046       
00047     
00048   // destructor must also be virtual
00049   virtual ~PDE();
00050 
00056   void Plot(Graphics *g, const int layer=0);
00057   
00063   void Plot(Graphics *g, CellularPotts *cpm, const int layer=0);
00064   
00071   void ContourPlot(Graphics *g, int layer=0, int colour=1);
00072     
00074   inline int SizeX() const {
00075     return sizex;
00076   }
00077   
00079   inline int SizeY() const {
00080     return sizey;
00081   }
00082   
00084   inline int Layers() const {
00085     return layers;
00086   }
00087 
00095   inline double Sigma(const int layer, const int x, const int y) const {
00096     return sigma[layer][x][y];
00097   }
00098   
00106   inline void setValue(const int layer, const int x, const int y, const double value) {
00107     sigma[layer][x][y]=value;
00108   }
00109   
00116   inline void addtoValue(const int layer, const int x, const int y, const double value) {
00117     sigma[layer][x][y]+=value;
00118   }
00119 
00125   inline double Max(int l) {
00126     double max=sigma[l][0][0];
00127     int loop=sizex*sizey;
00128     for (int i=1;i<loop;i++)
00129       if (sigma[l][0][i]>max) {
00130         max=sigma[l][0][i];
00131       }
00132     return max;
00133   }
00139   inline double Min(int l) {
00140     double min=sigma[l][0][0];
00141     int loop=sizex*sizey;
00142     for (int i=1;i<loop;i++)
00143       if (sigma[l][0][i]<min) {
00144         min=sigma[l][0][i];
00145       }
00146     return min;
00147   }
00148   
00160   void Diffuse(int repeat);
00161 
00165   void NoFluxBoundaries(void);
00166   
00170   void AbsorbingBoundaries(void);
00171 
00175   void PeriodicBoundaries(void);
00176 
00185   void Secrete(CellularPotts *cpm);
00186 
00189   inline double TheTime(void) const {
00190     return thetime;
00191   }
00192   
00198   double GetChemAmount(const int layer=-1);
00199 
00210   void GradC(int layer=0, int first_grad_layer=1); 
00211 
00225   void PlotVectorField(Graphics &g, int stride, int linelength, int first_grad_layer=1);
00226 
00227  protected:
00228 
00229   double ***sigma;
00230   
00231   // Used as temporary memory in the diffusion step
00232   // (addresses will be swapped for every time step, so
00233   // never directly use them!!! Access is guaranteed to be correct
00234   // through user interface)
00235 
00236   double ***alt_sigma;
00237  
00238   int sizex;
00239   int sizey;
00240   int layers;
00241  
00242  
00243   // Protected member functions
00244 
00251   virtual int MapColour(double val);
00252 
00254   PDE(void);
00255   
00261   virtual double ***AllocateSigma(const int layers, const int sx, const int sy);
00262  
00263  private:
00264   static const int nx[9], ny[9];
00265   double thetime;
00266 
00267 };
00268 
00269 
00270 #endif
```

---

Generated on Tue Dec 12 16:32:40 2006 for Tissue Simulation Toolkit by

1.3.5 
