# Supplementary material for: Contact-Inhibited Chemotaxis in De Novo and Sprouting Blood-Vessel Growth
Source: PLoS Comput Biol. 2008 Sep 19;4(9):e1000163. doi: 10.1371/journal.pcbi.1000163 (PMC2528254; doi:10.1371/journal.pcbi.1000163)
Supplement: Protocol S1 — Tissue Simulation Toolkit v0.1.3. The source code for the software used for the simulations presented in this paper is also available from http://sourceforge.net/projects/tst. Installation: Unpack and compile according to the instructions given in the INSTALL file The code is written in C++ using the cross-platform (Windows, Mac, or Unix/Linux) library Qt (available from www.trolltech.com). (332 KB ZIP) [file pcbi.1000163.s002.zip › TST0.1.3/html/qtgraph_8h-source.html]

Tissue Simulation Toolkit: /home/romer/TST0.1.3/qtgraph.h Source File

Main Page | Namespace List | Class Hierarchy | Class List | File List | Namespace Members | Class Members | File Members

# /home/romer/TST0.1.3/qtgraph.h

Go to the documentation of this file.

```
00001 /* 
00002 
00003 Copyright 1996-2006 Roeland Merks
00004 
00005 This file is part of Tissue Simulation Toolkit.
00006 
00007 Tissue Simulation Toolkit is free software; you can redistribute
00008 it and/or modify it under the terms of the GNU General Public
00009 License as published by the Free Software Foundation; either
00010 version 2 of the License, or (at your option) any later version.
00011 
00012 Tissue Simulation Toolkit is distributed in the hope that it will
00013 be useful, but WITHOUT ANY WARRANTY; without even the implied
00014 warranty of MERCHANTABILITY or FITNESS FOR A PARTICULAR PURPOSE.
00015 See the GNU General Public License for more details.
00016 
00017 You should have received a copy of the GNU General Public License
00018 along with Tissue Simulation Toolkit; if not, write to the Free
00019 Software Foundation, Inc., 51 Franklin St, Fifth Floor, Boston, MA
00020 02110-1301 USA
00021 
00022 */
00023 #ifndef _QTGRAPH_H_
00024 #define _QTGRAPH_H_
00025 #include <qwidget.h>
00026 #include <qlabel.h>
00027 #include <qpainter.h>
00028 #include <q3picture.h>
00029 #include <qpixmap.h>
00030 //Added by qt3to4:
00031 #include <QMouseEvent>
00032 #include <QPaintEvent>
00033 #include "graph.h"
00034 
00035 
00036 
00037 class QtGraphics : public QWidget, public Graphics {
00038 
00039   Q_OBJECT
00040 
00041     public:
00042   QtGraphics(int xfield, int yfield, const char *movie_file=0);
00043   QtGraphics(QWidget *parent, const char *name, int xfield, int yfield, const char *movie_file=0) : QWidget(parent, name) {
00044     QtGraphics(xfield, yfield, movie_file);
00045   }
00046   
00047   virtual ~QtGraphics(void);
00048   virtual void BeginScene(void);
00049   virtual void EndScene(void);
00050   /*inline void Flush(void) {
00051     XFlush(display);
00052     } */
00053   virtual void Point( int colour, int i, int j);
00054   virtual void Line ( int x1, int y1,int x2,int y2,int colour );
00055   /*void Field (const int **r, int mag=1);
00056     void PlotNumber(int number, int x, int y);*/
00057   
00058   virtual int GetXYCoo(int *X,int *Y);
00059   /*char *ChangeTitle (const char *message);
00060   void RecoverTitle(void);*/
00061   //LineType CropSize(void);
00062   //Coordinate ReplaceBeast(Coordinate old_size,Coordinate new_size);
00063   virtual int XField(void) const {return width();}
00064   virtual int YField(void) const {return height();}
00065 
00066   virtual void Write(char *fname, int quality=-1);
00067   inline void ClearImage(void) {
00068 
00069     pixmap->fill();
00070   }
00071   
00072   virtual void TimeStep(void);
00073 
00074   public slots:
00075     void TimeStepWrap(void);
00076 
00077   signals:
00078     void SimulationDone(void);
00079 
00080  private:
00081   void paintEvent( QPaintEvent* );
00082   void mousePressEvent( QMouseEvent *e);
00083   void mouseReleaseEvent( QMouseEvent *e);
00084   QPainter *paint;
00085   QPainter *paint2;
00086   QLabel *label;
00087   Q3Picture pic;
00088   QPen *pens;
00089   QTimer *timer;
00090   QPixmap *pixmap;
00091   QLabel *image;
00092 
00093   int mouse_x;
00094   int mouse_y;
00095   Qt::ButtonState mouse_button;
00096 
00097   // private methods
00098   void ReadColorTable(QPen *pens);
00099   QTimer *t;
00100   
00101   
00102   
00103 };
00104 
00105 #include <qapplication.h>
00106 //#include <qsignal.h>
00107 
00108 #define TIMESTEP void QtGraphics::TimeStep(void)
00109 #endif
```

---

Generated on Tue Dec 12 16:32:40 2006 for Tissue Simulation Toolkit by

1.3.5 
