# Supplementary material for: Contact-Inhibited Chemotaxis in De Novo and Sprouting Blood-Vessel Growth
Source: PLoS Comput Biol. 2008 Sep 19;4(9):e1000163. doi: 10.1371/journal.pcbi.1000163 (PMC2528254; doi:10.1371/journal.pcbi.1000163)
Supplement: Protocol S1 — Tissue Simulation Toolkit v0.1.3. The source code for the software used for the simulations presented in this paper is also available from http://sourceforge.net/projects/tst. Installation: Unpack and compile according to the instructions given in the INSTALL file The code is written in C++ using the cross-platform (Windows, Mac, or Unix/Linux) library Qt (available from www.trolltech.com). (332 KB ZIP) [file pcbi.1000163.s002.zip › TST0.1.3/html/random_8cpp.html]

Tissue Simulation Toolkit: random.cpp File Reference

Main Page | Namespace List | Class Hierarchy | Class List | File List | Namespace Members | Class Members | File Members

# /home/romer/TST0.1.3/random.cpp File Reference

`#include <stdio.h>`  
`#include <stdlib.h>`  
`#include <sys/timeb.h>`  
`#include <iostream>`  
`#include "random.h"`  

|  |
| --- |
|  |
| Functions | |
| double | RANDOM (void) |
| int | Seed (int seed) |
| long | RandomNumber (long max) |
| void | AskSeed (void) |
| int | Randomize (void) |

---

## Function Documentation

|  |  |  |  |  |  |  |
| --- | --- | --- | --- | --- | --- | --- |
| |  |  |  |  |  |  | | --- | --- | --- | --- | --- | --- | | void AskSeed | ( | void |  | ) |  | |

|  |  |  |  |
| --- | --- | --- | --- |
|  | Interactively ask for the seed **Parameters:**  |  |  | | --- | --- | | *void* |  |  **Returns:**  void |

|  |  |  |  |  |  |  |
| --- | --- | --- | --- | --- | --- | --- |
| |  |  |  |  |  |  | | --- | --- | --- | --- | --- | --- | | double RANDOM | ( | void |  | ) |  | |

|  |  |
| --- | --- |
|  | **Returns:**  A random double between 0 and 1 |

|  |  |  |  |  |  |  |
| --- | --- | --- | --- | --- | --- | --- |
| |  |  |  |  |  |  | | --- | --- | --- | --- | --- | --- | | int Randomize | ( | void |  | ) |  | |

|  |  |  |  |
| --- | --- | --- | --- |
|  | Make a random seed based on the local time **Parameters:**  |  |  | | --- | --- | | *void* |  |  **Returns:**  void |

|  |  |  |  |  |  |  |
| --- | --- | --- | --- | --- | --- | --- |
| |  |  |  |  |  |  | | --- | --- | --- | --- | --- | --- | | long RandomNumber | ( | long | *max* | ) |  | |

|  |  |  |  |
| --- | --- | --- | --- |
|  | Returns a random integer value between 1 and 'max' **Parameters:**  |  |  | | --- | --- | | *The* | maximum value (long) |  **Returns:**  A random integer (long) |

|  |  |  |  |  |  |  |
| --- | --- | --- | --- | --- | --- | --- |
| |  |  |  |  |  |  | | --- | --- | --- | --- | --- | --- | | int Seed | ( | int | *seed* | ) |  | |

|  |  |  |  |
| --- | --- | --- | --- |
|  | **Parameters:**  |  |  | | --- | --- | | *An* | integer random seed |  **Returns:**  the random seed |

---

Generated on Tue Dec 12 16:32:41 2006 for Tissue Simulation Toolkit by

1.3.5
