# Supplementary material for: Contact-Inhibited Chemotaxis in De Novo and Sprouting Blood-Vessel Growth
Source: PLoS Comput Biol. 2008 Sep 19;4(9):e1000163. doi: 10.1371/journal.pcbi.1000163 (PMC2528254; doi:10.1371/journal.pcbi.1000163)
Supplement: Protocol S1 — Tissue Simulation Toolkit v0.1.3. The source code for the software used for the simulations presented in this paper is also available from http://sourceforge.net/projects/tst. Installation: Unpack and compile according to the instructions given in the INSTALL file The code is written in C++ using the cross-platform (Windows, Mac, or Unix/Linux) library Qt (available from www.trolltech.com). (332 KB ZIP) [file pcbi.1000163.s002.zip › TST0.1.3/html/sticky_8h-source.html]

Tissue Simulation Toolkit: /home/romer/TST0.1.3/sticky.h Source File

Main Page | Namespace List | Class Hierarchy | Class List | File List | Namespace Members | Class Members | File Members

# /home/romer/TST0.1.3/sticky.h

Go to the documentation of this file.

```
00001 /* 
00002 
00003 Copyright 1996-2006 Roeland Merks
00004 
00005 This file is part of Tissue Simulation Toolkit.
00006 
00007 Tissue Simulation Toolkit is free software; you can redistribute
00008 it and/or modify it under the terms of the GNU General Public
00009 License as published by the Free Software Foundation; either
00010 version 2 of the License, or (at your option) any later version.
00011 
00012 Tissue Simulation Toolkit is distributed in the hope that it will
00013 be useful, but WITHOUT ANY WARRANTY; without even the implied
00014 warranty of MERCHANTABILITY or FITNESS FOR A PARTICULAR PURPOSE.
00015 See the GNU General Public License for more details.
00016 
00017 You should have received a copy of the GNU General Public License
00018 along with Tissue Simulation Toolkit; if not, write to the Free
00019 Software Foundation, Inc., 51 Franklin St, Fifth Floor, Boston, MA
00020 02110-1301 USA
00021 
00022 */
00023 #define GRIDX 100
00024 #define GRIDY 100
00025 #define MAXCELLS 1024 /*  beware  */
00026 #define MAXNEIGH  2054
00027 #define FALSE    0
00028 #define TRUE     1
00029 #define OK 1
00030 #define TESTCELLS 1
00031 #define BOLTZMANN 1024 
00032 
00033 
00034 #define MAXSEED 65531349
00035 
00036 /* #define SAMPLE POPSIZE/2 */
00037 
00038 //#define DEBUG 0
00039 #define MAXHIST 4096
00040 #define MAXTYPE 256
00041 #define WHITE 0
00042 #define BLACK 1
00043 #define RED 2
00044 #define BLUE 3
00045 #define GREEN 4
00046 #define MEDIUM 0
00047 #define EMPTY -1
00048 #define DIV 1   /* DIV 2^(# of divbit) */
00049 #define REMARK 56
00050 #define HASHCOLPRIME 255
00051 #define PLOTPERIODFREQUENCY 25
00052 #define HOSTDEAD -20
00053 #define NULL_BEAST -10
00054 #define OK_BEAST -5
00055 
00056 /* the number of external connections per genome
00057    of random DNA's */
00058 /* "parameters" */
00059 
00060 #define PMUT 0.009
00061 #define PMUT2 .1
00062 #define DMUT 0.0021
00063 #define PCO 0.
00064 #define NETSIZE 29 
00065 #define INETSIZE 16 
00066 #define INP_PROT 16 /* number of negative inputs (connectivity) */
00067 #define POTPROT 16 /* number of potential proteins */
00068 //#define EXTCONNECTIONS 0
00069 #define COMMUNICATION 2
00070 #define ENERGYOFFSET 8
00071 #define NHHIST 10
00072 #define NH_TH 0.5
00073 #define ENDOFSTATES 1<<(NETSIZE+2)
00074 /* function definitions */
00075 
00076 double RANDOM();
00077 
00078 
00079 
00080 
00081 
00082 
00083 
00084 
00085 
00086 
00087 
00088
```

---

Generated on Tue Dec 12 16:32:40 2006 for Tissue Simulation Toolkit by

1.3.5 
