# Supplementary material for: Contact-Inhibited Chemotaxis in De Novo and Sprouting Blood-Vessel Growth
Source: PLoS Comput Biol. 2008 Sep 19;4(9):e1000163. doi: 10.1371/journal.pcbi.1000163 (PMC2528254; doi:10.1371/journal.pcbi.1000163)
Supplement: Protocol S1 — Tissue Simulation Toolkit v0.1.3. The source code for the software used for the simulations presented in this paper is also available from http://sourceforge.net/projects/tst. Installation: Unpack and compile according to the instructions given in the INSTALL file The code is written in C++ using the cross-platform (Windows, Mac, or Unix/Linux) library Qt (available from www.trolltech.com). (332 KB ZIP) [file pcbi.1000163.s002.zip › TST0.1.3/html/sticky_8h.html]

Tissue Simulation Toolkit: sticky.h File Reference

Main Page | Namespace List | Class Hierarchy | Class List | File List | Namespace Members | Class Members | File Members

# /home/romer/TST0.1.3/sticky.h File Reference

Go to the source code of this file.

|  |
| --- |
|  |
| Defines | |
| #define | GRIDX   100 |
| #define | GRIDY   100 |
| #define | MAXCELLS   1024 |
| #define | MAXNEIGH   2054 |
| #define | FALSE   0 |
| #define | TRUE   1 |
| #define | OK   1 |
| #define | TESTCELLS   1 |
| #define | BOLTZMANN   1024 |
| #define | MAXSEED   65531349 |
| #define | MAXHIST   4096 |
| #define | MAXTYPE   256 |
| #define | WHITE   0 |
| #define | BLACK   1 |
| #define | RED   2 |
| #define | BLUE   3 |
| #define | GREEN   4 |
| #define | MEDIUM   0 |
| #define | EMPTY   -1 |
| #define | DIV   1 |
| #define | REMARK   56 |
| #define | HASHCOLPRIME   255 |
| #define | PLOTPERIODFREQUENCY   25 |
| #define | HOSTDEAD   -20 |
| #define | NULL\_BEAST   -10 |
| #define | OK\_BEAST   -5 |
| #define | PMUT   0.009 |
| #define | PMUT2   .1 |
| #define | DMUT   0.0021 |
| #define | PCO   0. |
| #define | NETSIZE   29 |
| #define | INETSIZE   16 |
| #define | INP\_PROT   16 |
| #define | POTPROT   16 |
| #define | COMMUNICATION   2 |
| #define | ENERGYOFFSET   8 |
| #define | NHHIST   10 |
| #define | NH\_TH   0.5 |
| #define | ENDOFSTATES   1<<(NETSIZE+2) |
| Functions | |
| double | RANDOM () |

---

## Define Documentation

|  |  |
| --- | --- |
| |  | | --- | | #define BLACK   1 | |

|  |  |
| --- | --- |
|  |  |

|  |  |
| --- | --- |
| |  | | --- | | #define BLUE   3 | |

|  |  |
| --- | --- |
|  |  |

|  |  |
| --- | --- |
| |  | | --- | | #define BOLTZMANN   1024 | |

|  |  |
| --- | --- |
|  |  |

|  |  |
| --- | --- |
| |  | | --- | | #define COMMUNICATION   2 | |

|  |  |
| --- | --- |
|  |  |

|  |  |
| --- | --- |
| |  | | --- | | #define DIV   1 | |

|  |  |
| --- | --- |
|  |  |

|  |  |
| --- | --- |
| |  | | --- | | #define DMUT   0.0021 | |

|  |  |
| --- | --- |
|  |  |

|  |  |
| --- | --- |
| |  | | --- | | #define EMPTY   -1 | |

|  |  |
| --- | --- |
|  |  |

|  |  |
| --- | --- |
| |  | | --- | | #define ENDOFSTATES   1<<(NETSIZE+2) | |

|  |  |
| --- | --- |
|  |  |

|  |  |
| --- | --- |
| |  | | --- | | #define ENERGYOFFSET   8 | |

|  |  |
| --- | --- |
|  |  |

|  |  |
| --- | --- |
| |  | | --- | | #define FALSE   0 | |

|  |  |
| --- | --- |
|  |  |

|  |  |
| --- | --- |
| |  | | --- | | #define GREEN   4 | |

|  |  |
| --- | --- |
|  |  |

|  |  |
| --- | --- |
| |  | | --- | | #define GRIDX   100 | |

|  |  |
| --- | --- |
|  |  |

|  |  |
| --- | --- |
| |  | | --- | | #define GRIDY   100 | |

|  |  |
| --- | --- |
|  |  |

|  |  |
| --- | --- |
| |  | | --- | | #define HASHCOLPRIME   255 | |

|  |  |
| --- | --- |
|  |  |

|  |  |
| --- | --- |
| |  | | --- | | #define HOSTDEAD   -20 | |

|  |  |
| --- | --- |
|  |  |

|  |  |
| --- | --- |
| |  | | --- | | #define INETSIZE   16 | |

|  |  |
| --- | --- |
|  |  |

|  |  |
| --- | --- |
| |  | | --- | | #define INP\_PROT   16 | |

|  |  |
| --- | --- |
|  |  |

|  |  |
| --- | --- |
| |  | | --- | | #define MAXCELLS   1024 | |

|  |  |
| --- | --- |
|  |  |

|  |  |
| --- | --- |
| |  | | --- | | #define MAXHIST   4096 | |

|  |  |
| --- | --- |
|  |  |

|  |  |
| --- | --- |
| |  | | --- | | #define MAXNEIGH   2054 | |

|  |  |
| --- | --- |
|  |  |

|  |  |
| --- | --- |
| |  | | --- | | #define MAXSEED   65531349 | |

|  |  |
| --- | --- |
|  |  |

|  |  |
| --- | --- |
| |  | | --- | | #define MAXTYPE   256 | |

|  |  |
| --- | --- |
|  |  |

|  |  |
| --- | --- |
| |  | | --- | | #define MEDIUM   0 | |

|  |  |
| --- | --- |
|  |  |

|  |  |
| --- | --- |
| |  | | --- | | #define NETSIZE   29 | |

|  |  |
| --- | --- |
|  |  |

|  |  |
| --- | --- |
| |  | | --- | | #define NH\_TH   0.5 | |

|  |  |
| --- | --- |
|  |  |

|  |  |
| --- | --- |
| |  | | --- | | #define NHHIST   10 | |

|  |  |
| --- | --- |
|  |  |

|  |  |
| --- | --- |
| |  | | --- | | #define NULL\_BEAST   -10 | |

|  |  |
| --- | --- |
|  |  |

|  |  |
| --- | --- |
| |  | | --- | | #define OK   1 | |

|  |  |
| --- | --- |
|  |  |

|  |  |
| --- | --- |
| |  | | --- | | #define OK\_BEAST   -5 | |

|  |  |
| --- | --- |
|  |  |

|  |  |
| --- | --- |
| |  | | --- | | #define PCO   0. | |

|  |  |
| --- | --- |
|  |  |

|  |  |
| --- | --- |
| |  | | --- | | #define PLOTPERIODFREQUENCY   25 | |

|  |  |
| --- | --- |
|  |  |

|  |  |
| --- | --- |
| |  | | --- | | #define PMUT   0.009 | |

|  |  |
| --- | --- |
|  |  |

|  |  |
| --- | --- |
| |  | | --- | | #define PMUT2   .1 | |

|  |  |
| --- | --- |
|  |  |

|  |  |
| --- | --- |
| |  | | --- | | #define POTPROT   16 | |

|  |  |
| --- | --- |
|  |  |

|  |  |
| --- | --- |
| |  | | --- | | #define RED   2 | |

|  |  |
| --- | --- |
|  |  |

|  |  |
| --- | --- |
| |  | | --- | | #define REMARK   56 | |

|  |  |
| --- | --- |
|  |  |

|  |  |
| --- | --- |
| |  | | --- | | #define TESTCELLS   1 | |

|  |  |
| --- | --- |
|  |  |

|  |  |
| --- | --- |
| |  | | --- | | #define TRUE   1 | |

|  |  |
| --- | --- |
|  |  |

|  |  |
| --- | --- |
| |  | | --- | | #define WHITE   0 | |

|  |  |
| --- | --- |
|  |  |

---

## Function Documentation

|  |  |  |  |  |  |  |
| --- | --- | --- | --- | --- | --- | --- |
| |  |  |  |  |  |  | | --- | --- | --- | --- | --- | --- | | double RANDOM | ( | void |  | ) |  | |

|  |  |
| --- | --- |
|  | **Returns:**  A random double between 0 and 1 |

---

Generated on Tue Dec 12 16:32:41 2006 for Tissue Simulation Toolkit by

1.3.5
