# Supplementary material for: Contact-Inhibited Chemotaxis in De Novo and Sprouting Blood-Vessel Growth
Source: PLoS Comput Biol. 2008 Sep 19;4(9):e1000163. doi: 10.1371/journal.pcbi.1000163 (PMC2528254; doi:10.1371/journal.pcbi.1000163)
Supplement: Protocol S1 — Tissue Simulation Toolkit v0.1.3. The source code for the software used for the simulations presented in this paper is also available from http://sourceforge.net/projects/tst. Installation: Unpack and compile according to the instructions given in the INSTALL file The code is written in C++ using the cross-platform (Windows, Mac, or Unix/Linux) library Qt (available from www.trolltech.com). (332 KB ZIP) [file pcbi.1000163.s002.zip › TST0.1.3/html/structli.html]

Tissue Simulation Toolkit: li struct Reference

Main Page | Namespace List | Class Hierarchy | Class List | File List | Namespace Members | Class Members | File Members

# li Struct Reference

`#include <x11graph.h>`

List of all members.

|  |
| --- |
|  |
| Public Attributes | |
| int | x1 |
| int | y1 |
| int | x2 |
| int | y2 |

---

## Member Data Documentation

|  |  |
| --- | --- |
| |  | | --- | | int li::x1 | |

|  |  |
| --- | --- |
|  |  |

|  |  |
| --- | --- |
| |  | | --- | | int li::x2 | |

|  |  |
| --- | --- |
|  |  |

|  |  |
| --- | --- |
| |  | | --- | | int li::y1 | |

|  |  |
| --- | --- |
|  |  |

|  |  |
| --- | --- |
| |  | | --- | | int li::y2 | |

|  |  |
| --- | --- |
|  |  |

---

The documentation for this struct was generated from the following file:

- /home/romer/TST0.1.3/x11graph.h

---

Generated on Tue Dec 12 16:32:41 2006 for Tissue Simulation Toolkit by

1.3.5
