# Supplementary material for: Contact-Inhibited Chemotaxis in De Novo and Sprouting Blood-Vessel Growth
Source: PLoS Comput Biol. 2008 Sep 19;4(9):e1000163. doi: 10.1371/journal.pcbi.1000163 (PMC2528254; doi:10.1371/journal.pcbi.1000163)
Supplement: Protocol S1 — Tissue Simulation Toolkit v0.1.3. The source code for the software used for the simulations presented in this paper is also available from http://sourceforge.net/projects/tst. Installation: Unpack and compile according to the instructions given in the INSTALL file The code is written in C++ using the cross-platform (Windows, Mac, or Unix/Linux) library Qt (available from www.trolltech.com). (332 KB ZIP) [file pcbi.1000163.s002.zip › TST0.1.3/html/warning_8cpp.html]

Tissue Simulation Toolkit: warning.cpp File Reference

Main Page | Namespace List | Class Hierarchy | Class List | File List | Namespace Members | Class Members | File Members

# /home/romer/TST0.1.3/warning.cpp File Reference

`#include <stdarg.h>`  
`#include <stdio.h>`  
`#include <stdlib.h>`  
`#include "warning.h"`  

|  |
| --- |
|  |
| Functions | |
| void | error (char \*fmt,...) |
| void | warning (char \*fmt,...) |
| Variables | |
| int | Quiet = 0 |

---

## Function Documentation

|  |  |  |  |  |  |  |  |  |  |  |  |  |
| --- | --- | --- | --- | --- | --- | --- | --- | --- | --- | --- | --- | --- |
| |  |  |  |  | | --- | --- | --- | --- | | void error | ( | char \* | *fmt*, | |  |  | ... |  | |  | ) |  | | |

|  |  |
| --- | --- |
|  |  |

|  |  |  |  |  |  |  |  |  |  |  |  |  |
| --- | --- | --- | --- | --- | --- | --- | --- | --- | --- | --- | --- | --- |
| |  |  |  |  | | --- | --- | --- | --- | | void warning | ( | char \* | *fmt*, | |  |  | ... |  | |  | ) |  | | |

|  |  |
| --- | --- |
|  |  |

---

## Variable Documentation

|  |  |
| --- | --- |
| |  | | --- | | int Quiet = 0 | |

|  |  |
| --- | --- |
|  |  |

---

Generated on Tue Dec 12 16:32:41 2006 for Tissue Simulation Toolkit by

1.3.5
