# Supplementary material for: Contact-Inhibited Chemotaxis in De Novo and Sprouting Blood-Vessel Growth
Source: PLoS Comput Biol. 2008 Sep 19;4(9):e1000163. doi: 10.1371/journal.pcbi.1000163 (PMC2528254; doi:10.1371/journal.pcbi.1000163)
Supplement: Protocol S1 — Tissue Simulation Toolkit v0.1.3. The source code for the software used for the simulations presented in this paper is also available from http://sourceforge.net/projects/tst. Installation: Unpack and compile according to the instructions given in the INSTALL file The code is written in C++ using the cross-platform (Windows, Mac, or Unix/Linux) library Qt (available from www.trolltech.com). (332 KB ZIP) [file pcbi.1000163.s002.zip › TST0.1.3/html/x11graph_8cpp.html]

Tissue Simulation Toolkit: x11graph.cpp File Reference

Main Page | Namespace List | Class Hierarchy | Class List | File List | Namespace Members | Class Members | File Members

# /home/romer/TST0.1.3/x11graph.cpp File Reference

`#include <stdio.h>`  
`#include <unistd.h>`  
`#include <X11/Xlib.h>`  
`#include <X11/Xutil.h>`  
`#include <X11/keysym.h>`  
`#include <X11/cursorfont.h>`  
`#include <math.h>`  
`#include <malloc.h>`  
`#include <stdlib.h>`  
`#include <sys/types.h>`  
`#include <sys/stat.h>`  
`#include <fcntl.h>`  
`#include <cstring>`  
`#include <errno.h>`  
`#include <png.h>`  
`#include "sticky.h"`  
`#include "x11graph.h"`  
`#include "parameter.h"`  
`#include "output.h"`  
`#include <iostream>`  
`#include <string>`  

|  |
| --- |
|  |
| Defines | |
| #define | NOPVM |
| #define | KEYBUFSIZE   10 |
| #define | SWAP(a, b)   tmpswap = a; a = b; b = tmpswap; |
| Variables | |
| Parameter | par |
| int | errno |

---

## Define Documentation

|  |  |
| --- | --- |
| |  | | --- | | #define KEYBUFSIZE   10 | |

|  |  |
| --- | --- |
|  |  |

|  |  |
| --- | --- |
| |  | | --- | | #define NOPVM | |

|  |  |
| --- | --- |
|  |  |

|  |  |  |  |  |  |  |  |  |  |
| --- | --- | --- | --- | --- | --- | --- | --- | --- | --- |
| |  |  |  |  |  |  |  |  |  | | --- | --- | --- | --- | --- | --- | --- | --- | --- | | #define SWAP | ( | a,|  |  |  |  |  |  | | --- | --- | --- | --- | --- | --- | |  |  | b |  | ) | tmpswap = a; a = b; b = tmpswap; | | |

|  |  |
| --- | --- |
|  |  |

---

## Variable Documentation

|  |  |
| --- | --- |
| |  | | --- | | int errno | |

|  |  |
| --- | --- |
|  |  |

|  |  |
| --- | --- |
| |  | | --- | | Parameter par | |

|  |  |
| --- | --- |
|  |  |

---

Generated on Tue Dec 12 16:32:41 2006 for Tissue Simulation Toolkit by

1.3.5
