# Supplementary material for: Contact-Inhibited Chemotaxis in De Novo and Sprouting Blood-Vessel Growth
Source: PLoS Comput Biol. 2008 Sep 19;4(9):e1000163. doi: 10.1371/journal.pcbi.1000163 (PMC2528254; doi:10.1371/journal.pcbi.1000163)
Supplement: Protocol S1 — Tissue Simulation Toolkit v0.1.3. The source code for the software used for the simulations presented in this paper is also available from http://sourceforge.net/projects/tst. Installation: Unpack and compile according to the instructions given in the INSTALL file The code is written in C++ using the cross-platform (Windows, Mac, or Unix/Linux) library Qt (available from www.trolltech.com). (332 KB ZIP) [file pcbi.1000163.s002.zip › TST0.1.3/html/x11graph_8h-source.html]

Tissue Simulation Toolkit: /home/romer/TST0.1.3/x11graph.h Source File

Main Page | Namespace List | Class Hierarchy | Class List | File List | Namespace Members | Class Members | File Members

# /home/romer/TST0.1.3/x11graph.h

Go to the documentation of this file.

```
00001 /* 
00002 
00003 Copyright 1996-2006 Roeland Merks
00004 
00005 This file is part of Tissue Simulation Toolkit.
00006 
00007 Tissue Simulation Toolkit is free software; you can redistribute
00008 it and/or modify it under the terms of the GNU General Public
00009 License as published by the Free Software Foundation; either
00010 version 2 of the License, or (at your option) any later version.
00011 
00012 Tissue Simulation Toolkit is distributed in the hope that it will
00013 be useful, but WITHOUT ANY WARRANTY; without even the implied
00014 warranty of MERCHANTABILITY or FITNESS FOR A PARTICULAR PURPOSE.
00015 See the GNU General Public License for more details.
00016 
00017 You should have received a copy of the GNU General Public License
00018 along with Tissue Simulation Toolkit; if not, write to the Free
00019 Software Foundation, Inc., 51 Franklin St, Fifth Floor, Boston, MA
00020 02110-1301 USA
00021 
00022 */
00023 
00034 #ifndef _XGRAPH_H_
00035 #define _XGRAPH_H_
00036 
00037 #include <X11/Xlib.h>
00038 #include <X11/Xutil.h>
00039 #include <X11/keysym.h>
00040 #include <X11/cursorfont.h>
00041 #include "graph.h"
00042 
00043 // Shared memory extensions
00044 
00045 #ifdef USE_XSHM
00046 #include <sys/ipc.h>
00047 #include <sys/shm.h>
00048 #include <X11/extensions/XShm.h>
00049 #endif
00050 
00051 //
00052 
00053 
00054 #define OUTFILE "beestje.mov"
00055 #define CFILE "sticky.ctb"
00056 
00057 #define VERBOSE 1
00058 #define RESIZE -20
00059 #define MOTION -21
00060 
00061 
00062 typedef struct li {
00063   
00064   int x1;
00065   int y1;
00066   int x2;
00067   int y2;
00068 
00069 } LineType;
00070 
00071 typedef struct co {
00072   long x;
00073   long y;
00074 } Coordinate;
00075 
00076 
00077 class X11Graphics : public Graphics {
00078 
00079 public:
00080   X11Graphics(int xfield, int yfield, const char *movie_file=0);
00081   virtual ~X11Graphics(void);
00082   virtual void BeginScene(void);
00083   virtual void EndScene(void);
00084 
00086   inline void Flush(void) {
00087     XFlush(display);
00088   }
00089 
00090   virtual void Point( int color, int x, int y);
00091   virtual void Line ( int x1, int y1,int x2,int y2,int colour );
00092   void Field (const int **r, int mag=1);
00093 
00094   virtual int GetXYCoo(int *X,int *Y);
00095   
00100   char *ChangeTitle (const char *message);
00101 
00102   
00104   void RecoverTitle(void);
00105   
00114   LineType CropSize(void);
00115   
00122 Coordinate ReplaceBeast(Coordinate old_size,Coordinate new_size); 
00123 
00124 virtual inline int XField(void) const {return xfield;} 
00125 
00126 virtual inline int YField(void) const {return yfield;} 
00127 
00128 virtual void Write(char *fname, int quality=-1); 
00129 
00133 inline void ClearImage(void) {
00134 
00135     for (int x=0;x<xfield;x++) {
00136         for (int y=0;y<yfield;y++) {
00137           Point(0,x,y);
00138         }
00139     }
00140   }
00141   virtual void TimeStep(void); 
00142 private:
00143   void InitGraphics(int xfield, int yfield);
00144   void CloseGraphics();
00145   void InitStore(void);
00146   int DetectControl();
00147   void StoreCompPict(void);
00148   void SendScene();
00149   void receiveScene(int machineindex, int beastindex, int ndish);
00150   void receiveScene1(int machineindex, int beastindex, int ndish);
00151   void KillCell(struct creature *beast);
00152   int ResizeField(struct creature *beast);
00153   void ReceiveScene(int machineindex, int beastindex, int ndish);
00154   void ReceiveScene1(int machineindex, int beastindex, int ndish);
00155   void ReadColorTable(XColor *colors);
00156   void MakeColorMap();
00157   int XExposep();
00158   int GetHeight(Window w);
00159   // Data members
00160   int LineClearP(char direction, int pos, int cropcol=0);
00161   void Resize(void);
00162   
00163 private:
00164 
00165 #ifdef USE_XSHM
00166   int shm=0;
00167   XShmSegmentInfo shminfo;
00168 #endif
00169   unsigned char *film;
00170   long count;
00171   int xfield,yfield;
00172   int dosendscene;
00173   /* int divided=0; */
00174 
00175   int hsize,vsize;
00176   char *title;
00177   Display *display;
00178   char *server;
00179   Window window;
00180   XImage *image;
00181   GC windowGC;
00182   XEvent event;
00183   XSizeHints hint;
00184   XWindowAttributes attributes;
00185   XSetWindowAttributes setattributes;
00186   int screen;
00187   int colourclass,color_screen;
00188   int depth;
00189   Visual *visual;
00190   unsigned long white, black, foreground, background;
00191   XColor *colors;
00192   Colormap new_colormap;
00193   char *image_data;
00194   unsigned char *movie_data;
00195   char *movie_file_name;
00196   bool compressed_movie_p;
00197   FILE *movie_fp;
00198   
00199   bool store;
00200 
00201   char *old_window_name;
00202 
00203   int pseudoCol8;
00204   XVisualInfo visual_info;
00205   XVisualInfo *visual_list;
00206 
00207 
00208 };
00209 #define TIMESTEP void X11Graphics::TimeStep(void)
00210 #endif
```

---

Generated on Tue Dec 12 16:32:41 2006 for Tissue Simulation Toolkit by

1.3.5 
