# Supplementary material for: Contact-Inhibited Chemotaxis in De Novo and Sprouting Blood-Vessel Growth
Source: PLoS Comput Biol. 2008 Sep 19;4(9):e1000163. doi: 10.1371/journal.pcbi.1000163 (PMC2528254; doi:10.1371/journal.pcbi.1000163)
Supplement: Protocol S1 — Tissue Simulation Toolkit v0.1.3. The source code for the software used for the simulations presented in this paper is also available from http://sourceforge.net/projects/tst. Installation: Unpack and compile according to the instructions given in the INSTALL file The code is written in C++ using the cross-platform (Windows, Mac, or Unix/Linux) library Qt (available from www.trolltech.com). (332 KB ZIP) [file pcbi.1000163.s002.zip › TST0.1.3/html/x11graph_8h.html]

Tissue Simulation Toolkit: x11graph.h File Reference

Main Page | Namespace List | Class Hierarchy | Class List | File List | Namespace Members | Class Members | File Members

# /home/romer/TST0.1.3/x11graph.h File Reference

`#include <X11/Xlib.h>`  
`#include <X11/Xutil.h>`  
`#include <X11/keysym.h>`  
`#include <X11/cursorfont.h>`  
`#include "graph.h"`  

Go to the source code of this file.

|  |
| --- |
|  |
| Classes | |
| struct | co |
| struct | li |
| class | X11Graphics |
|  | X-Windows implementation of Graphics interface.  More... |
| Defines | |
| #define | OUTFILE   "beestje.mov" |
| #define | CFILE   "sticky.ctb" |
| #define | VERBOSE   1 |
| #define | RESIZE   -20 |
| #define | MOTION   -21 |
| #define | TIMESTEP   void X11Graphics::TimeStep(void) |
| Typedefs | |
| typedef li | LineType |
| typedef co | Coordinate |

---

## Define Documentation

|  |  |
| --- | --- |
| |  | | --- | | #define CFILE   "sticky.ctb" | |

|  |  |
| --- | --- |
|  |  |

|  |  |
| --- | --- |
| |  | | --- | | #define MOTION   -21 | |

|  |  |
| --- | --- |
|  |  |

|  |  |
| --- | --- |
| |  | | --- | | #define OUTFILE   "beestje.mov" | |

|  |  |
| --- | --- |
|  |  |

|  |  |
| --- | --- |
| |  | | --- | | #define RESIZE   -20 | |

|  |  |
| --- | --- |
|  |  |

|  |  |
| --- | --- |
| |  | | --- | | #define TIMESTEP   void X11Graphics::TimeStep(void) | |

|  |  |
| --- | --- |
|  |  |

|  |  |
| --- | --- |
| |  | | --- | | #define VERBOSE   1 | |

|  |  |
| --- | --- |
|  |  |

---

## Typedef Documentation

|  |  |
| --- | --- |
| |  | | --- | | typedef struct co Coordinate | |

|  |  |
| --- | --- |
|  |  |

|  |  |
| --- | --- |
| |  | | --- | | typedef struct li LineType | |

|  |  |
| --- | --- |
|  |  |

---

Generated on Tue Dec 12 16:32:41 2006 for Tissue Simulation Toolkit by

1.3.5
